# Synthesis and Antiviral Evaluation of (1,4-Disubstituted-1,2,3-Triazol)-(*E*)-2-Methyl-but-2-Enyl Nucleoside Phosphonate Prodrugs

**DOI:** 10.3390/molecules26051493

**Published:** 2021-03-09

**Authors:** Tuniyazi Abuduaini, Vincent Roy, Julien Marlet, Catherine Gaudy-Graffin, Denys Brand, Cécile Baronti, Franck Touret, Bruno Coutard, Tamara R. McBrayer, Raymond F. Schinazi, Luigi A. Agrofoglio

**Affiliations:** 1 Institute of Organic and Analytical Chemistry, CNRS UMR 7311, Universite d’Orléans, F-45067 Orléans, France; tuniyazi.abuduaini@univ-orleans.fr; 2 Inserm U1259, Université de Tours, 37032 Tours, France; julien.marlet@univ-tours.fr (J.M.); catherine.gaudy-graffin@univ-tours.fr (C.G.-G.); denys.brand@univ-tours.fr (D.B.); 3 Unité des Virus Émergents (UVE: Aix-Marseille Univ, IRD 190, Inserm 1207, IHU Méditerranée Infection), 13000 Marseille, France; cecile.baronti@univ-amu.fr (C.B.); franck.TOURET@univ-amu.fr (F.T.); bruno.coutard@univ-amu.fr (B.C.); 4 Center for AIDS Research, Laboratory of Biochemical Pharmacology, Department of Pediatrics, Emory University School of Medicine and Children’s Healthcare of Atlanta, Atlanta, GA 30222, USA; tamara.mcbraye@emory.edu (T.R.M.); rschin@emory.edu (R.F.S.)

**Keywords:** nucleosides, olefin cross metathesis, ultrasound, copper-catalyzed azide-alkyne cycloaddition (CuAAC), antiviral properties, HBV, HIV, SARS-CoV-2

## Abstract

A series of hitherto unknown (1,4-disubstituted-1,2,3-triazol)-(*E*)-2-methyl-but-2-enyl nucleosides phosphonate prodrugs bearing 4-substituted-1,2,3-triazoles were prepared in a straight approach through an olefin acyclic cross metathesis as the key synthetic step. All novel compounds were evaluated for their antiviral activities against HBV, HIV and SARS-CoV-2. Among these molecules, only compound **15j**, a hexadecyloxypropyl (HDP)/(*isopropyloxycarbonyl*-oxymethyl)-ester (POC) prodrug, showed activity against HBV in Huh7 cell cultures with 62% inhibition at 10 μM, without significant cytotoxicity (IC_50_ = 66.4 μM in HepG2 cells, IC_50_ = 43.1 μM in HepG2 cells) at 10 μM.

## 1. Introduction

**Figure 1:**
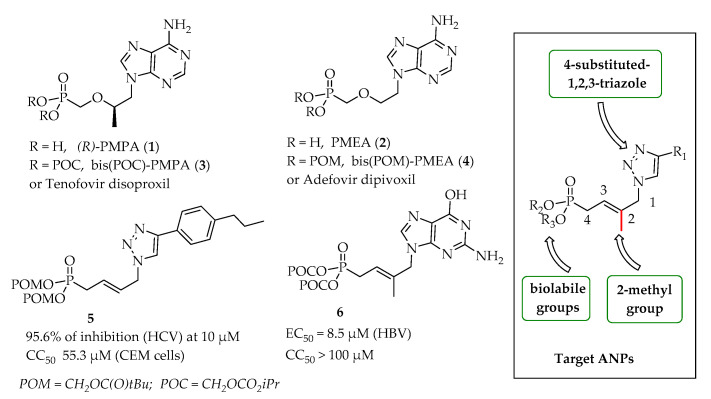
Structure of selected acyclic nucleoside phosphonates (ANPs) and target derivatives. PMPA: 2-(phosphonomethoxy)propyladenine; PMEA: 9-(2-(phosphonomethoxy)ethyl) adenine; POM: pivaloyloxymethyl; POC: (isopropyloxycarbonyl-oxymethyl)-ester.

Acyclic nucleoside phosphonates (ANPs), such as (*R*)-PMPA [9-[9(*R*)-2-(phosphonomethoxy)propyl]adenine, **1**] and PMEA [9-[2-(phosphonomethoxy)ethyl] adenine, **2**] discovered by A. Holý and E. De Clercq in 1986, led to a new family of nucleotide analogs which has attracted considerable attention, [1,2,3,4]. In order to improve the oral absorption of these phosphonate analogs, ANPs are delivered as prodrugs [*bis*(POC)-PMPA (**3**) or *bis*(POM)-PMEA (**4**)]; prodrug moiety [5] has previously focused on acyloxyalkyl-ester (pivaloyloxymethyl, POM) [6,7], or ((*isopropyloxycarbonyl*-oxymethyl)-ester, POC) [8], alkoxyalkyl groups (hexadecyloxypropyl, HDP) [9], and more recently on phosphonoamidates (ProTides) [10,11]. Currently, several ANP prodrugs (alone or in combination) are FDA-approved drugs against DNA and RNA viruses. In our search for antiviral compounds, we have discovered a new class of acyclic nucleoside phosphonates based on a 4-phosphono-but-2-en-1-yl skeleton, with the double bond having *trans* stereochemistry [12,13,14]. We have shown that this modification allows for the mimicry of the three-dimensional geometry provided by the backbone of PMEA, PMPA, and CDV ((*S*)-1-[3-hydroxy-2-(phosphonylmethoxy)propyl]cytosine) while maintaining an electronic contribution similar to that brought by the oxygen atom [12]. Our group has published a series of *bis*(POM)-(1,4-disubstituted-1,2,3-triazol)-(*E*)-but-2′-enyl nucleoside phosphonates [15]. Among them, the compound **5** exhibits significant potency against human hepatitis C virus (HCV) infections at 10 μM (95% of inhibition) meanwhile our synthesized *bis*(POC)-(E)-2-methyl-but-2-enylguanine **6** (unpublished data) showed antiviral activity against hepatitis B virus (HBV) with an EC_50_ of 8.5 μM without significant cytotoxicity (Figure 1).

For nucleosides, it appears that chemical alterations (such as a methyl group at 2′ position) at the sugar (or acyclic side-chain) moiety or at the heterocycle such as 1,2,3-triazoles [16] often lead to marked differences in antiviral activity. Based on these findings and on our data for compounds **5** and **6**, we were interested in the synthesis of hitherto unknown (1,4-disubstituted-1,2,3-triazol)-(*E*)-2-methyl-but-2-enyl nucleosides phosphonate prodrugs and we wish to compare the impact of bis(POC) with the mixed HDP/POC biolabile moiety on the antiviral activity. All compounds were evaluated against hepatitis B virus (HBV), human immunodeficiency virus (HIV-1) and the newly detected severe acute respiratory syndrome (SARS-CoV-2).

## 2. Results and Discussions

### 2.1. Chemistry

**Scheme 1:**
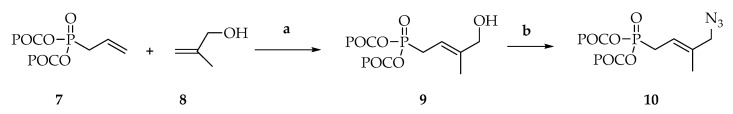
Reagents and conditions: (**a**) Hoveyda–Grubbs catalyst (15 mol%), dry CH_2_Cl_2_, 55 °C, 24 h, 94%; (**b**) (i) MsCl, Et_3_N, dry CH_2_Cl_2_, 0 °C to room temperature (rt), 40 min; (ii) NaN_3_, DMF, rt, 5 h, 93%.

First, we synthetized the *bis*(POC)allylphosphonate **7** from dimethylallylphosphonate according our previously reported method [14]. Then, optimized olefin cross-metathesis reaction between **7** and 2-methylprop-2-en-1-ol (**8**) was performed in dry dichloromethane with a Hoveyda–Grubbs catalyst (15 mol%) under ultrasonic irradiation at 55 °C during 24 h [17], (Scheme 1). The desired compound **9** was obtained in an excellent 94% yield with only *E*-isomer; no trace of *Z*-isomer was detected (NOESY NMR, see Supplementary Materials). Compound **9** was converted to the corresponding mesylate and the obtained sulfone was used directly in the next step without further purification. The introduction of the azido group on the mesylated compound was realized with sodium azide in DMF at room temperature for 5 h to afford compound **10** in excellent 93% yield.

Compound **10** was engaged in a regioselective copper-catalyzed azide-alkyne 1,3-dipolar cycloaddition (CuAAC) [18,19] with seventeen various terminal alkynes (dipolarophiles) selected to bear bulky, polar and apolar groups. The desired 1,4-disubstituted-1,2,3-triazoles **11a–q** were isolated in good yields ranging from 57% to 91% through adequate methods A or B (Table 1). Only compounds **11g** and **11i** were isolated in poor yields. For **11m**, the 2-nitrophenylboronic acid (method B) was added to the solution in order to avoid the decarboxylation of the desired compound [20].

**Scheme 2:**
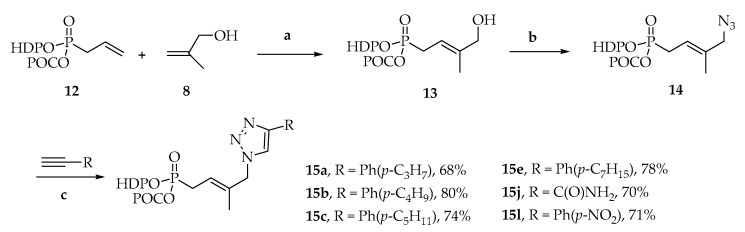
Reagents and conditions: (**a**) Hoveyda–Grubbs catalyst (15 mol%), dry CH_2_Cl_2_, 55 °C, 24 h, 88%; (**b**) (i) MsCl, Et_3_N, dry CH_2_Cl_2_, 0 °C to rt, 40 min; (ii) NaN_3_, DMF, rt, 5 h, 84%; (**c**) CuSO_4_.5H_2_O, sodium ascorbate, *t*BuOH/H_2_O (2:1), 40 °C, 2~18 h, 68~80%.

From the antiviral evaluation of **11a**–**q** (see hereafter), it appears that compounds **11a**, **11c**, **11e**, **11j**, and **11l** have a >40% inhibition of HBV, at 10 μM, with low cytotoxicity. Thus, we focused our attention only to the synthesis of their HDP/POC analogs **15a**, **15c**, **15e**, **15j**, **15l**, (Scheme 2). Starting from HDP/POC allylphosphonate **12**, obtained from dimethylallylphosphonate according to our previous method [14], a cross metathesis reaction of **12** and 2-methyl-2-propen-1-ol (**8**) in dry dichloromethane using the Hoveyda–Grubbs catalyst (15 mol%) under ultrasonic irradiation at 55 °C during 24 h afforded the desired compound **13** in 88% yield as only *E*-isomer.

**Figure 2:**
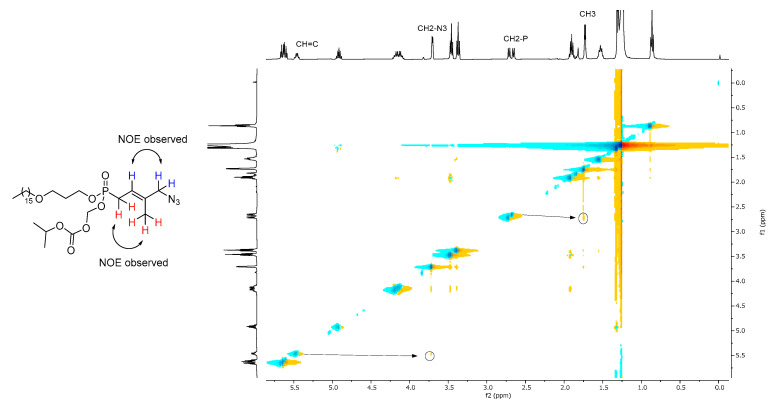
NOESY NMR spectra of **14**.

Compound **13** was then activated by mesylation from 0 °C to room temperature (rt) in dry dichloromethane in the presence of methanesulfonyl chloride during 40 min and the corresponding sulfone was directly engaged in the next step without further purification. Mesylated intermediate was then solubilized in DMF, in the presence of sodium azide at room temperature for 5 h to afford compound **14** in 84% yield. The structure of **14** was confirmed by NOESY NMR experiment (Figure 2).

Finally, the CuAAC reaction of **14** with various substituted phenylacetylenes and non-aromatic terminal alkynes afforded a series of HDP/POC-(1,4-disubstituted-1,2,3-triazol)-2′-methyl-but-2′-enylphosphonate **15a**, **15c**, **15e**, **15j**, **15l**, respectively, in good yields ranging from 68% to 80%.

### 2.2. Antiviral Evaluation

All synthesized compounds, the (1,4-disubstituted-1,2,3-triazol)-(*E*)-2-methyl-but-2-enyl nucleosides phosphonate prodrugs **11a**–**q**, **15a**, **15c**, **15e**, **15j**, **15l**, were tested for their antiviral activities in vitro against HIV, HBV and SARS-CoV-2 viruses; the results are summarized in Table 2 and represent means from triplicate wells. 

None of the compounds tested displayed anti-HIV activity compared to positive control AZT (EC_50_ of 0.008 μM (data not shown). None of the compounds displayed anti-SARS-CoV-2 activity compared to positive control remdesivir. Compounds **11a**,**c**–**e**, **11j** and **11l** were found to exhibit a moderate anti-HBV activity compared to entecavir (on Huh7 cells) and lamivudine (on HepAd38).

It is worth noting that the substituent on triazole ring has a dramatic effect on HBV activity. In fact, compounds with hydrophobic aryl groups (electron-donating **11a**–**e** and electron-withdrawing **11l**) exhibited good activity (except for **11b**), whereas triazolyl-ANPs with alkyl (**11f**,**g**) or polar substituents such as alcohol (**11h**), aldehyde (**11i**), formamide (**11j**) and amide (**11k**) and carboxylic derivatives (**11m**–**q**) have only low activity. We can speculate that the hydrophobic pocket (formed by A87, F88, P177, L180, and M2) found in the rear of the RT dNTP binding site of HBV could interact with hydrophobic aryl residues through donor atom-π interaction [21].

Compound **15j**, a HDP/POC prodrug, a ribavirin analog, is the most active drug against HBV in this serial, with 62% inhibition at 10 μM. However, generally speaking, the bis(POC) ester prodrugs are more active than their HDP/POC counterparts. The cytotoxicity of test compounds was performed in different cell systems. Positive control cycloheximide exhibited expected toxicity in the peripheral blood mononuclear (PBM) and HepG2 cells, with an IC_50_ (μM) of 1.0 and 1.5 μM, respectively (data not shown).

## 3. Materials and Methods

### 3.1. Chemistry General Section

Commercially available chemicals were provided as reagent grade and used as received. Some reactions requiring anhydrous conditions were carried out using oven-dried glassware and under an atmosphere of dry argon. All anhydrous solvents were provided from commercial sources as very dry reagents. The reactions were monitored by thin layer chromatography (TLC) analysis using silica gel precoated plates (Kieselgel 60F254, E. Merck). Compounds were visualized by UV irradiation and/or spraying with sulfuric acid (H_2_SO_4_ 5% in ethanol) stain followed by charring at average 150 °C. Flash column chromatography was performed on silica gel 60 M (0.040–0.063 mm, E. Merck). The infrared spectra were measured with the Perkin–Elmer Spectrometer. The ^1^H and ^13^C NMR spectra were recorded on the BrukerAvance DPX 250 or BrukerAvance 400 Spectrometer (Bruker, Champs sur Marne, France). Chemical shifts are given in ppm and are referenced to the deuterated solvent signal or to TMS as internal standard and multiplicities are reported as s (singlet), d (doublet), t (triplet), q (quartet) and m (multiplet). Carbon multiplicities were assigned by distortionless enhancement by polarization transfer (DEPT) experiments. ^1^H and ^13^C signals were attributed on the basis of H–H and H–C correlations. High resolution mass spectra were performed on a Bruker Q-TOF MaXis spectrometer (Bruker Daltonics, Bremen, Germany) by the “Fédération de Recherche” ICOA/CBM (FR2708) platform. LC-MS data were acquired on a Thermo-Fisher UHPLC-MSQ system equipped with an electron spray ionization source (ESI). The temperature of the source was maintained at 350 °C. Initially, the cone voltage was set at 35 V and after 5 min was increased to 75 V. In full scan mode, data were acquired between 100 and 1000 *m*/*z* in the positive mode with a 1.00 s scan time. In addition, a UV detection was performed with a diode array detector at three wavelengths 273, 254 and 290 nm, respectively. A water/methanol (70%/30%) solution mixture with 0.1% formic acid was used as the mobile phase. The composition of the mobile phase was increased to 100% methanol with 0.1% formic acid with a 7% ramp. The flow rate was set at 0.300 mL min^−1^. Samples diluted in the mobile phase were injected (3 μL) on a C18 column (X-terra, Waters), with a 2.1 mm internal diameter, and 100 mm length, and placed into an oven at 40 °C. The electronic extraction of ions was performed and the subsequent areas under the corresponding chromatographic peaks determined. The compounds’ name follows the IUPAC recommendations.

### 3.2. Antiviral Evaluation

#### 3.2.1. HBV Assay

In Huh7 cells: antiviral activity against HBV in cell culture was measured as previously described [22]. Briefly, Huh7 cells were transfected with plasmid pCI_HBVpg1820 containing 1.1 unit of HBV genome under the control of a human cytomegalovirus (CMV) promoter. The drugs were stored at 1000× in DMSO at −20 °C. For each experiment, the drugs were diluted in DMEM cell culture medium (Thermo Fisher Scientific, Artenay, France) to a final concentration of 10 μM. All drugs were tested in triplicate in two independent experiments. After 4 days of culture, intracapsid viral DNA was quantified by duplex real-time PCR, using primers described elsewhere, on a LightCycler 480 II apparatus (Roche Diagnostics France, Meylan, France) using TaqMan Universal PCR Mastermix II without UNG (Thermo Fisher Scientific, Artenay, France). Cells were quantified using primers by real-time PCR, using primers HPRT1_F (5′-TGCAGACTTTGCTTTCCTTGGTC) and HPRT1_R (5′-CAAGCTTGCGACCTTGACCATC), on a LightCycler 480 II apparatus (Roche) using a LightCycler^®^ 480 SYBR Green I Master (Roche Diagnostics France, Meylan, France). Cycling reactions were performed with the following parameters: 5 min at 95 °C, followed by 45 cycles of 10 s at 95 °C, 15 s at 60 °C, 10 s at 72 °C. The inhibition of HBV replication (%) and cell culture viability (%) were quantified using the following formulas.

$$Inhibition\;of\;HBV\;replication\;\left(\%\right)=1-\frac{{\;\mathrm{HBV}\;\mathrm{DNA}\;\mathrm{copy}\;\mathrm{number}}_{Drug}}{{\mathrm{HBV}\;\mathrm{DNA}\;\mathrm{copy}\;\mathrm{number}}_{Cell\;culture\;medium}\;}\;Cell\;viability\;\left(\%\right)=\;\frac{\;\mathrm{HPRT}1{\;\mathrm{DNA}\;\mathrm{copy}\;\mathrm{number}}_{Drug}}{\mathrm{HPRT}1{\;\mathrm{DNA}\;\mathrm{copy}\;\mathrm{number}}_{Cell\;culture\;medium}\;}$$

In HepAD38 cells [23] were seeded at 50,000 cells/well in collagen-coated 96-well plates. Test compounds or 2′,3′-didéoxy-3′-thiacytidin (3TC) (control) were added to HepAD38 cells to a final concentration of 10 μM.

Real-Time PCR for HBV DNA. The experiment lasted 7 days. On day 7, total DNA was purified from the supernatant using a commercially available kit (DNeasy 96 Blood & Tissue kit, Qiagen). The HBV DNA was amplified in a real-time PCR assay using a LightCycler 480 (Roche) as described elsewhere [24]. All samples were tested in duplicate in two to three independent experiments. Analysis: The concentration of compound that inhibited HBV DNA replication by 50% (EC_50_) was determined by linear regression.

#### 3.2.2. HIV Assay

The assay was performed as described by Schinazi et al. [25,26] with minor modifications [26]. Briefly, human PBM cells were stimulated with PHA/IL-2 prior to infection with HIV-1 LAI (MOI 0.1) for 5 days in the presence of test compounds with a final concentration of 10 μM (LA-338-366) or AZT (control). The supernatants were harvested, and the HIV-1 RT was quantified. The median effective concentrations (EC_50/90_) were determined using the method of Belen’kii and Schinazi [27].

#### 3.2.3. SARS-CoV-2 Assay

Vero E6 (ATCC CRL-1586) cells were grown in a minimal essential medium (MEM) (Life Technologies, Carlsbad, CA, USA) with 7.5% heat-inactivated fetal calf serum (FCS), at 37 °C with 5% CO_2_ with 1% penicillin/streptomycin (PS, 5000 U.mL^−1^ and 5000 μg.mL^−1^, respectively; Life Technologies) and supplemented with 1% non-essential amino acids (Life Technologies). SARS-CoV-2 strain BavPat1 was obtained from Pr Drosten through EVA GLOBAL (https://www.european-virus-archive.com, accessed on 23 February 2021). To prepare the virus working stock, a 25 cm^2^ culture flask of confluent Vero E6 cells growing with MEM medium with 2.5% FBS (Life Technologies) was inoculated at MOI 0.001. The cell supernatant medium was harvested at the peak of infection and supplemented with 25 mM HEPES (Sigma, St. Louis, MO, USA) before being stored frozen in small aliquots at −80 °C. All experiments were conducted in a BSL3 laboratory. One day prior to infection, for the antiviral screening 5 × 10^4^ Vero E6 cells were seeded in 100 of μL the assay medium (containing 2.5% FCS) in 96 well plates. The next day, four 2-fold serial dilutions of compounds (20 μM to 2.5 μM, in duplicate) were added to the cells (25 μL/well, in the assay medium) as well as two internal well controls of viral inhibition corresponding to the addition of 10 μM of Remdesivir (BLDpharm, Hyderabad, India). Three virus control wells were supplemented with 25 μL of assay medium (positive controls hereafter named vc) and three cell control wells were supplemented with 50 μL of the medium (negative controls, hereafter named nc). After 15 min, 25 μL of a virus mix diluted in 2.5% FCS-containing medium was added to the wells at MOI 0.002. Three days after infection, the cell supernatant media was discarded and CellTiter-Blue^®^ reagent (Promega, Madison, WI, USA) was added following the manufacturer’s instructions. Plates were incubated for 2 h prior to recording the fluorescence (560/590 nm) with a Tecan Infinite 200Pro machine. From the measured OD_590nm_, the inhibition percentage was calculated as follows: ((OD_590nm_ value- mean OD_590nm_ value of vc)/(mean OD_590nm_ of nc- mean OD_590nm_ value of vc))*100. All data obtained were analyzed using GraphPad Prism 7 software (Graph pad software).

#### 3.2.4. Cytotoxicity Assays

Assays were performed in human peripheral blood mononuclear (PBM) and human liver (HepG2) cells via MTS assay using the CellTiter 96^®^ Non-Radioactive Cell Proliferation (Promega) kit as previously described^3^. Cytotoxicity was expressed as the concentration of test compounds that inhibited cell proliferation by 50% (IC_50_) and calculated using the Chou and Talalay method [28].

### 3.3. Experimental Section

(*E*)-4-Hydroxy-3-methyl-but-2-enyl-bis(POC)phosphonate (**9**)

To a solution of *bis*(POC) allylphosphonate (**7**) (500 mg, 2.0 eq., 1.41 mmol) and 2-methyl-2-propen-1-ol (**8**) (59 μL, 1.0 eq., 0.71 mmol) in dry CH_2_Cl_2_ (5 mL), Hoveyda–Grubbs catalyst (66 mg, 15 mol%, 0.11 mmol) was added. The catalyst addition was performed in five equal portions of 3 mol% (13.20 mg, 0.021 mmol) at t = 0, 3, 6, 9 and 21 h over the course of the reaction. The solution was sonicated at 55 °C under nitrogen atmosphere for 24 h. Volatiles were evaporated and the residue was purified by silica gel column chromatography (EtOAc/PE, 7:3) to give the desired phosphonate derivative **9** (264 mg, 94%) as brown oil. ^1^H NMR (400 MHz, CDCl_3_) *δ* 5.65–5.57 (m, 4H, O-CH_2_-O), 5.41–5.37 (m, 1H, CH=C), 4.87 (sept., *J* = 6.2 Hz, 2H, CH(CH_3_)_2_), 3.98 (d, *J* = 5.3 Hz, 2H, CH_2_-OH), 2.69 (dd, *J* = 22.7, 7.6 Hz, 2H, CH_2_-P), 2.34 (s, 1H, OH), 1.65 (d, *J* = 4.5 Hz, 3H, CH_3_), 1.28 (d, *J* = 6.3 Hz, 12H, CH(CH_3_)_2_). ^13^C NMR (101 MHz, CDCl_3_) *δ* 153.2 (C=O), 141.6, 141.5 (CH=C), 111.6, 111.5 (CH=C), 84.1, 84.1 (O-CH_2_-O), 73.3 (CH(CH_3_)_2_), 68.0, 68.0 (CH_2_-OH), 27.3, 25.9 (CH_2_-P), 21.6 (CH(CH_3_)_2_), 13.9, 13.9 (CH_3_). ^31^P NMR (162 MHz, CDCl_3_) δ 28.80. HRMS (ESI): *m*/*z* [M+H]^+^ calcd for C_15_H_28_O_10_P: 399.14146, found: 399.14151.

(*E*)-4-Azido-3-methyl-but-2-enyl-bis(POC)phosphonate (**10**)

To a solution of (*E*)-4-hydroxy-3-methyl-but-2-enyl-*bis*(POC)phosphonate **9** (264 mg, 1.0 equiv., 0.66 mmol) in anhydrous CH_2_Cl_2_ (5 mL) were added dropwise methanesulfonyl chloride (56 μL, 1.1 equiv., 0.73 mmol) and anhydrous triethylamine (102 μL, 1.1 equiv., 0.73 mmol) at 0 °C under the argon. After 30 min stirring at room temperature, the mixture was diluted with CH_2_Cl_2_ and washed with brine solution (3 × 10 mL). The organic layers were dried over MgSO_4_, filtrated and concentrated in vacuo. The residue was directly used for the next step without further purification. Thus, it was dissolved in anhydrous DMF (8 mL) and sodium azide (216 mg, 5.0 equiv., 3.32 mmol) was added under nitrogen atmosphere. After 5 h stirring at room temperature, the mixture was diluted with EtOAc, washed with water and then extracted with EtOAc. The combined organic layers were washed with brine solution (5 × 10 mL), dried over MgSO_4_, and concentrated in vacuo. The residue was purified by silica gel column chromatography (EtOAc/PE, 4:6) to give **10** (261 mg, 93%) as a colorless oil. ^1^H NMR (400 MHz, CDCl_3_) *δ* 5.71–5.61 (m, 4H, O-CH_2_-O), 5.48–5.42 (m, 1H, CH=C), 4.92 (sept, *J* = 6.2 Hz, 2H, CH(CH_3_)_2_), 3.71 (d, *J* = 4.0 Hz, 2H, CH_2_-N_3_), 2.75 (dd, *J* = 23.1, 7.9, Hz, 2H, CH_2_-P), 1.73 (d, *J* = 4.5 Hz, 3H, CH_3_), 1.32 (d, *J* = 6.2 Hz, 12H, CH(CH_3_)_2_). ^13^C NMR (101 MHz, CDCl_3_) *δ* 153.2 (C=O), 136.2, 136.1 (C=CH), 116.4, 116.3 (CH=C), 84.2, 84.1 (O-CH_2_-O), 73.3 (CH(CH_3_)_2_), 58.6, 58.6 (CH_2_-N_3_), 27.6 (CH_2_-P), 26.2 (CH_2_-P), 21.6 (CH(CH_3_)_2_), 14.9, 14.9 (CH_3_). ^31^P NMR (162 MHz, CDCl_3_) δ 27.85. IR *ν*_max_ 2985.61, 2937.37, 2100.12, 1755.01, 1255.06, 1031.52, 984.34, 949.97, 904.26, 832.67, 788.47 cm^−1^. HRMS (ESI): *m*/*z* [M+Na]^+^ calcd for C_15_H_26_N_3_NaO_9_P: 446.12988, found: 446.12990.

General procedures A for CuAAC reaction

To a solution of (*E*)-4-Azido-3-methyl-but-2-enyl-*bis*(POC)phosphonate **10** (1.0 equiv.) and alkyne (1.3 equiv.) in *t*BuOH/H_2_O (2:1) were added sodium ascorbate (0.6 equiv.) and CuSO_4_.5H_2_O (0.1 equiv.). The resulting suspension was stirred at 40 °C until completion (followed by TLC), then the crude mixture was co-evaporated five times with methanol (10 mL). The residue was purified by silica gel column chromatography using elution gradient of petroleum ether/ethyl acetate to give the desired *bis*(POC) phosphonate derivatives.

[[(*E*)-4-[4-(4-Propylphenyl)triazol-1-yl]-3-methyl-but-2-enyl]-(isopropoxycarbonyloxymethoxy)phosphoryl]oxymethyl isopropyl carbonate (**11a**)

The title compound was prepared from **10** (101 mg, 0.24 mmol) and 1-ethynyl-4-propylbenzene (49 μL mg, 0.31 mmol) following the general procedure A; the resulting suspension was stirred for 2 h at 40 °C. After purification on a silica gel column chromatography (EtOAc/PE, 4:6), the desired pure compound **11a** (112 mg, 83%) was obtained as a colorless oil. ^1^H NMR (250 MHz, CDCl_3_) δ 7.76 (s, 1H, H^5^), 7.74 (d, *J* = 5.7 Hz, 2H, H^Ar^), 7.25–7.17 (d, *J* = 8.5 Hz, 2H, H^Ar^), 5.77–5.51 (m, 4H, O-CH_2_-O; 1H, CH=C), 4.98–4.80 (m, 2H, CH_2_-N; 2H, CH(CH_3_)_2_), 2.78 (dd, *J* = 23.1, 7.8 Hz, 2H, CH_2_-P), 2.59 (t, *J* = 7.6 Hz, 2H, Ar-CH_2_), 1.70–1.58 (m, 3H, CH_3_; 2H, Ar-CH_2_-CH_2_), 1.27 (d, *J* = 6.3 Hz, 12H, CH(CH_3_)_2_), 0.93 (t, *J* = 7.3 Hz, 3H, Ar-CH_2_-CH_2_-CH_3_). ^13^C NMR (101 MHz, CDCl_3_) *δ* 153.2 (C=O), 148.3 (C^q,Ar^), 142.7 (C^q,Ar^), 135.9, 135.7 (CH=C), 128.9 (CH^Ar^), 128.1 (C^q,Ar^), 125.6 (CH^Ar^), 119.0 (CH^Ar^), 118.1, 118.0 (CH=C), 84.1, 84.1 (O-CH_2_-O), 73.4 (CH(CH_3_)_2_), 57.8, 57.8 (CH_2_-N), 37.8 (Ar-CH_2_), 27.8, 26.4 (CH_2_-P), 24.5 (Ar-CH_2_-CH_2_), 21.6, 21.6 (CH(CH_3_)_2_), 14.2, 14.2 (CH_3_), 13.8 (Ar-CH_2_-CH_2_-CH_3_). ^31^P NMR (162 MHz, CDCl_3_) *δ* 27.47. HRMS (ESI): *m*/*z* [M+H]^+^ calcd for C_26_H_39_N_3_O_9_P: 568.24184, found: 568.24217.

[[(*E*)-4-[4-(4-Butylphenyl)triazol-1-yl]-3-methyl-but-2-enyl]-(isopropoxycarbonyloxymethoxy)phosphoryl]oxymethyl isopropyl carbonate (**11b**)

The title compound was prepared from **10** (102 mg, 0.24 mmol) and 1-butyl-4-ethynylbenzene (55 μL, 0.31 mmol) following the general procedure A; the resulting suspension was stirred for 2 h at 40 °C. After purification on a silica gel column chromatography (EtOAc/PE, 4:6), the desired pure compound **11b** (123 mg, 88%) was obtained as a colorless oil. ^1^H NMR (250 MHz, CDCl_3_) *δ* 7.76 (d, *J* = 7.8 Hz, 2H, H^Ar^), 7.75 (s, 1H, H^5^), 7.22 (d, *J* = 7.8 Hz, 2H, H^Ar^), 5.73–5.63 (m, 4H, O-CH_2_-O), 5.60–5.54 (m, 1H, CH=C), 4.94 (d, *J* = 5.0 Hz, 2H, CH_2_-N), 4.90 (sept., *J* = 6.3 Hz, 2H, CH(CH_3_)_2_), 2.80 (dd, *J* = 23.3, 7.9 Hz, 2H, CH_2_-P), 2.62 (t, *J* = 7.8 Hz, 2H, Ar-CH_2_-CH_2_-CH_2_-CH_3_), 1.64–1.58 (m, 3H, CH_3_; 2H, Ar-CH_2_-CH_2_-CH_2_-CH_3_), 1.40–1.34 (m, 2H, Ar-CH_2_-CH_2_-CH_2_-CH_3_), 1.28 (d, *J* = 6.3 Hz, 12H, CH(CH_3_)_2_), 0.92 (t, *J* = 7.3 Hz, 3H, Ar-CH_2_-CH_2_-CH_2_-CH_3_). ^13^C NMR (101 MHz, CDCl_3_) *δ* 153.2 (C=O), 148.4 (C^q,Ar^), 143.0 (C^q,Ar^), 135.9, 135.8 (CH=C), 128.8 (CH_Ar_), 128.0 (C^q,Ar^), 125.7 (CH^Ar^), 119.0 (CH^Ar^), 118.1, 118.0 (CH=C), 84.2, 84.1 (O-CH_2_-O), 73.4 (CH(CH_3_)_2_), 57.8, 57.8 (CH_2_-N), 35.4 (Ar-CH_2_-CH_2_-CH_2_-CH_3_), 33.5 (Ar-CH_2_-CH_2_-CH_2_-CH_3_), 27.8 (CH_2_-P), 26.4 (CH_2_-P), 22.3 (Ar-CH_2_-CH_2_-CH_2_-CH_3_), 21.6, 21.6 (CH(CH_3_)_2_), 14.2, 14.2 (CH_3_), 13.9 (Ar-CH_2_-CH_2_-CH_2_-CH_3_). ^31^P NMR (162 MHz, CDCl_3_) *δ* 27.46. HRMS (ESI): *m*/*z* [M+Na]^+^ calcd for C_27_H_40_N_3_NaO_9_P: 604.23943, found: 604.23935.

[[(*E*)-4-[4-(4-Pentylphenyl)triazol-1-yl]-3-methyl-but-2-enyl]-(isopropoxycarbonyloxymethoxy)phosphoryl]oxymethyl isopropyl carbonate (**11c**)

The title compound was prepared from **10** (96 mg, 0.23 mmol) and 1-ethynyl-4-penthylbenzene (57 μL, 0.29 mmol) following the general procedure A; the resulting suspension was stirred for 2 h at 40 °C. After purification on a silica gel column chromatography (EtOAc/PE, 4:6), the desired pure compound **11c** (115 mg, 85%) was obtained as a colorless oil. ^1^H NMR (400 MHz, CDCl_3_) *δ* 7.76 (s, 2H, H^Ar^), 7.74 (s, 1H, H^5^), 7.22 (d, *J* = 7.8 Hz, 2H, H^Ar^), 5.72–5.64 (m, 4H, O-CH_2_-O), 5.61–5.56 (m, 1H, CH=C), 4.94 (d, *J* = 5.1 Hz, 2H, CH_2_-N), 4.90 (sept., *J* = 6.2 Hz, 2H, CH(CH_3_)_2_), 2.80 (dd, *J* = 23.1, 7.8 Hz, 2H, CH_2_-P), 2.62 (t, *J* = 7.8 Hz, 2H, Ar-CH_2_-(CH_2_)_3_-CH_3_), 1.64–1.59 (m, 3H, CH_3_; 2H, Ar-CH_2_-CH_2_-CH_2_-CH_2_-CH_3_), 1.33–1.32 (m, 4H, Ar-CH_2_-CH_2_-CH_2_-CH_2_-CH_3_), 1.28 (d, *J* = 6.3 Hz, 12H, CH(CH_3_)_2_), 0.89 (t, *J* = 7.3 Hz, 3H, Ar-(CH_2_)_4_-CH_3_). ^13^C NMR (101 MHz, CDCl_3_) *δ* 153.2 (C=O), 148.4 (C^q,Ar^), 143.0 (C^q,Ar^), 135.9, 135.7 (CH=C), 128.8 (CH_Ar_), 128.0 (C^q,Ar^), 125.7 (CH^Ar^), 119.0 (CH^Ar^), 118.1, 118.0 (CH=C), 84.2, 84.1 (O-CH_2_-O), 73.4 (CH(CH_3_)_2_), 57.8, 57.8 (CH_2_-N), 35.7 (Ar-CH_2_-(CH_2_)_3_-CH_3_), 31.4, 31.1 (Ar-CH_2_-CH_2_-CH_2_-CH_2_-CH_3_), 27.8 (CH_2_-P), 26.4 (CH_2_-P), 23.9 (Ar-CH_2_-CH_2_-CH_2_-CH_2_-CH_3_), 22.5 (Ar-(CH_2_)_3_CH_2_-CH_3_), 21.6, 21.6 (CH(CH_3_)_2_), 14.2, 14.2 (CH_3_), 14.0 (Ar-(CH_2_)_4_-CH_3_). ^31^P NMR (162 MHz, CDCl_3_) *δ* 27.51. HRMS (ESI): *m*/*z* [M+Na]^+^ calcd for C_28_H_42_N_3_NaO_9_P: 618.25508, found: 618.25473.

[[(*E*)-4-[4-(4-Hexylphenyl)triazol-1-yl]-3-methyl-but-2-enyl]-(isopropoxycarbonyloxymethoxy)phosphoryl]oxymethyl isopropyl carbonate (**11d**)

The title compound was prepared from **10** (93 mg, 0.22 mmol) and 1-ethynyl-4-hexylbenzene (60 μL mg, 0.29 mmol) following the general procedure A; the resulting suspension was stirred for 2 h at 40 °C. After purification on a silica gel column chromatography (EtOAc/PE, 35:65), the desired pure compound **11d** (112 mg, 84%) was obtained as a yellow oil. ^1^H NMR (400 MHz, CDCl_3_) *δ* 7.75 (d, *J* = 5.4 Hz, 2H, H^Ar^), 7.72 (s, 1H, H^5^), 7.20 (d, *J* = 7.8 Hz, 2H, H^Ar^), 5.70–5.62 (m, 4H, O-CH_2_-O), 5.59–5.53 (m, 1H, CH=C), 4.92 (d, *J* = 3.8 Hz, 2H, CH_2_-N), 4.88 (sept., *J* = 6.2 Hz, 2H, CH(CH_3_)_2_), 2.78 (dd, *J* = 23.1, 7.8 Hz, 2H, CH_2_-P), 2.60 (t, *J* = 7.8 Hz, 2H, Ar-CH_2_-(CH_2_)_4_-CH_3_), 1.62–1.56 (m, 3H, CH_3_; 2H, Ar-CH_2_-CH_2_-(CH_2_)_3_CH_3_), 1.33–1.25 (m, 18H, Ar-CH_2_-CH_2_-(CH_2_)_3_CH_3,_ CH(CH_3_)_2_), 0.86 (t, *J* = 7.0 Hz, 3H, Ar-(CH_2_)_5_-CH_3_). ^13^C NMR (101 MHz, CDCl_3_) *δ* 153.2 (C=O), 148.3 (C^q,Ar^), 143.0 (C^q,Ar^), 135.9, 135.8 (CH=C), 128.8 (CH_Ar_), 128.0 (C^q,Ar^), 125.7 (CH^Ar^), 119.1 (CH^Ar^), 118.1, 117.9 (CH=C), 84.2, 84.1 (O-CH_2_-O), 73.4 (CH(CH_3_)_2_), 57.8, 57.7 (CH_2_-N), 35.7 (Ar-CH_2_-(CH_2_)_4_-CH_3_), 31.7, 31.3 (Ar-CH_2_-CH_2_-(CH_2_)_3_CH_3_), 28.9 (Ar-CH_2_-CH_2_-CH_2_-CH_2_-CH_2_-CH_3_), 27.8 (CH_2_-P), 26.4 (CH_2_-P), 23.9 (Ar-CH_2_-CH_2_-CH_2_-CH_2_-CH_2_-CH_3_), 22.6 (Ar-(CH_2_)_4_CH_2_-CH_3_), 21.6, 21.6 (CH(CH_3_)_2_), 14.2, 14.21 (CH_3_), 14.1 (Ar-(CH_2_)_5_-CH_3_). ^31^P NMR (162 MHz, CDCl_3_) *δ* 27.50. HRMS (ESI): *m*/*z* [M+Na]^+^ calcd for C_29_H_44_N_3_NaO_9_P: 632.27073, found: 632.27074.

[[(*E*)-4-[4-(4-Heptylphenyl)triazol-1-yl]-3-methyl-but-2-enyl]-(isopropoxycarbonyloxymethoxy)phosphoryl]oxymethyl isopropyl carbonate (**11e**)

The title compound was prepared from **10** (113 mg, 0.27 mmol) and 1-ethynyl-4-heptylbenzene (78 μL, 0.35 mmol) following the general procedure A; the resulting suspension was stirred for 2 h at 40 °C. After purification on a silica gel column chromatography (EtOAc/PE, 3:7), the desired pure compound **11e** (151 mg, 91%) was obtained as a yellow oil. ^1^H NMR (400 MHz, CDCl_3_) *δ* 7.76 (d, *J* = 2.4 Hz, 2H, H^Ar^), 7.74 (s, 1H, H^5^), 7.22 (d, *J* = 7.8 Hz, 2H, H^Ar^), 5.72–5.64 (m, 4H, O-CH_2_-O), 5.62–5.56 (m, 1H, CH=C), 4.94 (d, *J* = 3.8 Hz, 2H, CH_2_-N), 4.89 (sept., *J* = 6.3 Hz, 2H, CH(CH_3_)_2_), 2.79 (dd, *J* = 23.1, 7.8 Hz, 2H, CH_2_-P), 2.61 (t, *J* = 7.8 Hz, 2H, Ar-CH_2_-(CH_2_)_5_-CH_3_), 1.64–1.58 (m, 3H, CH_3_; 2H, Ar-CH_2_-CH_2_-(CH_2_)_4_CH_3_), 1.33–1.25 (m, 20H, Ar-CH_2_-CH_2_-(CH_2_)_4_CH_3,_ CH(CH_3_)_2_), 0.87 (t, *J* = 7.1 Hz, 3H, Ar-(CH_2_)_6_-CH_3_). ^13^C NMR (101 MHz, CDCl_3_) *δ* 153.2 (C=O), 148.4 (C^q,Ar^), 143.0 (C^q,Ar^), 135.9, 135.8 (CH=C), 128.8 (CH_Ar_), 128.0 (C^q,Ar^), 125.6 (CH^Ar^), 119.0 (CH^Ar^), 118.2, 118.1 (CH=C), 84.2, 84.1 (O-CH_2_-O), 73.4 (CH(CH_3_)_2_), 57.9, 57.8 (CH_2_-N), 35.7 (Ar-CH_2_-(CH_2_)_5_-CH_3_), 31.8, 31.4 (Ar-CH_2_-CH_2_-(CH_2_)_4_CH_3_), 29.7, 29.2, 29.1 (Ar-CH_2_-CH_2_-CH_2_-CH_2_-CH_2_-CH_2_-CH_3_), 27.8 (CH_2_-P), 26.4 (CH_2_-P), 22.6 (Ar-(CH_2_)_5_CH_2_-CH_3_), 21.6, 21.5 (CH(CH_3_)_2_), 14.2, 14.1 (CH_3_), 14.1 (Ar-(CH_2_)_6_-CH_3_). ^31^P NMR (162 MHz, CDCl_3_) *δ* 27.46. HRMS (ESI): *m*/*z* [M+H]^+^ calcd for C_30_H_47_N_3_O_9_P: 624.30444, found: 624.30373.

[[(*E*)-4-(4-Cyclopropyltriazol-1-yl)-3-methyl-but-2-enyl]-(isopropoxycarbonyloxymethoxy)phosphoryl]oxymethyl isopropyl carbonate (**11f**)

The title compound was prepared from **10** (94 mg, 0.22 mmol) and cyclopropylacetylene (24 μL, 0.29 mmol) following the general procedure A; the resulting suspension was stirred for 2 h at 40 °C. After purification on a silica gel column chromatography (EtOAc/PE, 4:6), the desired pure compound **11f** (86 mg, 79%) was obtained as a colorless oil. ^1^H NMR (250 MHz, CDCl_3_) *δ* 7.23 (s, 1H, H^5^), 5.72–5.62 (m, 4H, O-CH_2_-O), 5.54–5.45 (m, 1H, CH=C), 4.92 (sept., *J* = 6.2 Hz, 2H, CH(CH_3_)_2_), 4.83 (d, *J* = 4.1 Hz, 2H, CH_2_-N), 2.76 (dd, *J* = 23.1, 7.8 Hz, 2H, CH_2_-P), 2.00–1.89 (m, 1H, CH_2_CHCH_2_), 1.59 (d, *J* = 5.5 Hz, 3H, CH_3_), 1.31 (dd, *J* = 6.3, 1.3 Hz, 12H, CH(CH_3_)_2_), 0.95–0.82 (m, 4H, CH_2_CH_2_). ^13^C NMR (101 MHz, CDCl_3_) *δ* 153.2 (C=O), 150.7 (C^q,Ar^), 136.01, 135.9 (CH=C), 119.4 (CH^Ar^), 117.8, 117.7 (CH=C), 84.1, 84.1 (O-CH_2_-O), 73.4 (CH(CH_3_)_2_), 57.6, 57.6 (CH_2_-N), 27.7, 26.4 (CH_2_-P), 21.7, 21.6 (CH(CH_3_)_2_), 14.2, 14.1 (CH_3_), 7.8 (CH_2_CH_2_), 6.7 (CH_2_CHCH_2_). ^31^P NMR (162 MHz, CDCl_3_) *δ* 27.6. HRMS (ESI): *m*/*z* [M+H]^+^ calcd for C_20_H_33_N_3_O_9_P: 490.19489, found: 490.19492.

[[(*E*)-4-(4-tert-Butyltriazol-1-yl)-3-methyl-but-2-enyl]-(isopropoxycarbonyloxymethoxy)phosphoryl]oxymethyl isopropyl carbonate (**11g**)

The title compound was prepared from **10** (98 mg, 0.23 mmol) and 3,3-dimethyl-1-butyne (37 μL, 0.30 mmol) following the general procedure A; the resulting suspension was stirred for 24 h at 40 °C. After purification on a silica gel column chromatography (EtOAc/PE, 6:4), the desired pure compound **11g** (56 mg, 48%) was obtained as a colorless oil. ^1^H NMR (400 MHz, CDCl_3_) *δ* 7.25 (s, 1H, H^5^), 5.71–5.62 (m, 4H, O-CH_2_-O), 5.51–5.45 (m, 1H, CH=C), 4.92 (sept., *J* = 6.2 Hz, 2H, CH(CH_3_)_2_), 4.85 (d, *J* = 3.9 Hz, 2H, CH_2_-N), 2.76 (dd, *J* = 23.1, 7.8 Hz, 2H, CH_2_-P), 1.60 (d, *J* = 4.5 Hz, 3H, CH_3_), 1.33 (s, 9H, C(CH)_3_), 1.31 (d, *J* = 6.3, Hz, 12H, CH(CH_3_)_2_). ^13^C NMR (101 MHz, CDCl_3_) *δ* 158.2 (C^q,Ar^), 153.1 (C=O), 136.1, 136.0 (CH=C), 118.4 (CH^Ar^), 117.6, 117.5 (CH=C), 84.2, 84.1 (O-CH_2_-O), 73.4 (CH(CH_3_)_2_), 57.6, 57.5 (CH_2_-N), 30.8 (C(CH)_3_), 30.3 (C(CH)_3_), 27.7, 26.3 (CH_2_-P), 21.6, 21.5 (CH(CH_3_)_2_), 14.3, 14.2 (CH_3_). ^31^P NMR (162 MHz, CDCl_3_) *δ* 27.62. HRMS (ESI): *m*/*z* [M+H]^+^ calcd for C_21_H_37_N_3_O_9_P: 506.22619, found: 506.22601.

[[(*E*)-4-[4-(Hydroxymethyl)triazol-1-yl]-3-methyl-but-2-enyl]-(isopropoxycarbonyloxymethoxy)phosphoryl]oxymethyl isopropyl carbonate (**11h**)

The title compound was prepared from **10** (105 mg, 0.25 mmol) and propargyl alcohol (19 μL, 0.32 mmol) following the general procedure A; the resulting suspension was stirred for 24 h at 40 °C. After purification on a silica gel column chromatography (MeOH/CH_2_Cl_2_, 5/95), the desired pure compound **11h** (84 mg, 71%) was obtained as a yellow oil. ^1^H NMR (400 MHz, CDCl_3_) *δ* 7.55 (s, 1H, H^5^), 5.67–5.59 (m, 4H, O-CH_2_-O), 5.49–5.44 (m, 1H, CH=C), 4.92–4.86 (m, 4H, CH(CH_3_)_2_), CH_2_-N), 4.75 (s, 2H, CH_2_OH), 2.74 (dd, *J* = 23.1, 7.8 Hz, 2H, CH_2_-P), 1.57 (d, *J* = 4.6 Hz, 3H, CH_3_), 1.28 (d, *J* = 5.6, Hz, 12H, CH(CH_3_)_2_). ^13^C NMR (101 MHz, CDCl_3_) *δ* 153.2 (C=O), 135.7, 135.6 (CH=C), 121.5 (CH^Ar^), 118.1, 118.0 (CH=C), 84.1, 84.0 (O-CH_2_-O), 73.5 (CH(CH_3_)_2_), 57.7, 57.6 (CH_2_-N), 56.5 (CH_2_OH), 27.7, 26.3 (CH_2_-P), 21.6, 21.5 (CH(CH_3_)_2_), 14.3, 14.2 (CH_3_). ^31^P NMR (162 MHz, CDCl_3_) *δ* 27.40. HRMS (ESI): *m*/*z* [M+Na]^+^ calcd for C_18_H_30_N_3_NaO_10_P: 502.156102, found: 502.155885.

[[(*E*)-4-(4-Formyltriazol-1-yl)-3-methyl-but-2-enyl]-(isopropoxycarbonyloxymethoxy)phosphoryl]oxymethyl isopropyl carbonate (**11i**)

The title compound was prepared from **10** (82 mg, 0.19 mmol) and 3,3-diethoxy-1-propyne (36 μL, 0.25 mmol) following the general procedure A; the resulting suspension was stirred for 2 h at 40 °C. After purification on a silica gel column chromatography (EtOAc/PE, 6/4), the desired pure compound **11i** (19 mg, 21%) was obtained as a colorless oil. ^1^H NMR (400 MHz, CDCl_3_) *δ* 10.14 (s, 1H, CHO), 8.14 (s, 1H, H^5^), 5.71–5.63 (m, 4H, O-CH_2_-O), 5.60–5.57 (m, 1H, CH=C), 4.98 (d, *J* = 3.9 Hz, 2H, CH_2_-N), 4.92 (sept., *J* = 6.2 Hz, 2H, CH(CH_3_)_2_), 2.78 (dd, *J* = 23.3, 7.8 Hz, 2H, CH_2_-P), 1.63 (d, *J* = 4.8 Hz, 3H, CH_3_), 1.31 (d, *J* = 6.2 Hz, 12H, CH(CH_3_)_2_). ^13^C NMR (101 MHz, CDCl_3_) *δ* 184.9 (CHO), 153.1 (C=O), 148.0 (C^q,Ar^), 134.8, 134.6 (CH=C), 125.2 (CH^Ar^), 119.5, 119.4 (CH=C), 84.2, 84.1 (O-CH_2_-O), 73.5 (CH(CH_3_)_2_), 58.1, 58.0 (CH_2_-N), 27.8, 26.4 (CH_2_-P), 21.6, 21.5 (CH(CH_3_)_2_), 14.3, 14.2 (CH_3_). ^31^P NMR (162 MHz, CDCl_3_) *δ* 27.04. HRMS (ESI): *m*/*z* [M+H]^+^ calcd for C_18_H_29_N_3_O_10_P: 478.158507, found: 478.158468.

[[(*E*)-4-(4-Carbamoyltriazol-1-yl)-3-methyl-but-2-enyl]-(isopropoxycarbonyloxymethoxy)phosphoryl]oxymethyl isopropyl carbonate (**11j**)

The title compound was prepared from **10** (106 mg, 0.25 mmol) and propiolamide (22 mg, 0.33 mmol) following the general procedure A; the resulting suspension was stirred for 14 h at 40 °C. After purification on a silica gel column chromatography (MeOH/CH_2_Cl_2_, 3/97), the desired pure compound **11j** (96 mg, 78%) was obtained as a colorless oil. ^1^H NMR (400 MHz, Acetone-d_6_) *δ* 8.31 (s, 1H, H^5^), 7.38 (s, 1H, NH), 6.78 (s, 1H, NH), 5.70–5.63 (m, 4H, O-CH_2_-O), 5.59–5.57 (m, 1H, CH=C), 5.08 (d, *J* = 4.0 Hz, 2H, CH_2_-N), 4.91 (sept., *J* = 6.2 Hz, 2H, CH(CH_3_)_2_), 2.84 (dd, *J* = 22.4, 7.9 Hz, 2H, CH_2_-P), 1.66 (d, *J* = 3.9 Hz, 3H, CH_3_), 1.29 (d, *J* = 6.3 Hz, 12H, CH(CH_3_)_2_). ^13^C NMR (101 MHz, Acetone-d_6_) *δ* 161.6 (NH_2_C=O), 153.1 (C=O), 143.4 (C^q,Ar^), 135.4, 135.3 (CH=C), 126.0 (CH^Ar^), 118.4, 118.3 (CH=C), 84.2, 84.1 (O-CH_2_-O), 72.8 (CH(CH_3_)_2_), 57.2, 57.1 (CH_2_-N), 27.3, 25.9 (CH_2_-P), 20.9 (CH(CH_3_)_2_), 13.6, 13.5 (CH_3_). ^31^P NMR (162 MHz, CDCl_3_) *δ* 27.05. HRMS (ESI): *m*/*z* [M+H]^+^ calcd for C_18_H_30_N_4_O_10_P: 493.169406, found: 493.169337.

[[(*E*)-4-(4-Octanoylaminomethyltriazol-1-yl)-3-methyl-but-2-enyl]-(isopropoxycarbonyloxymethoxy)phosphoryl]oxymethyl isopropyl carbonate (**11k**)

The title compound was prepared from **10** (109 mg, 0.26 mmol) and *N*-*N*(prop-2-yn-1-yl)octanamide (56 mg, 0.33 mmol) following the general procedure A; the resulting suspension was stirred for 24 h at 40 °C. After purification on a silica gel column chromatography (MeOH/CH_2_Cl_2_, 3/97), the desired pure compound **11k** (147 mg, 94%) was obtained as a yellowish oil. ^1^H NMR (400 MHz, CDCl_3_) *δ* 7.51 (s, 1H, H^5^), 6.20 (s, 1H, NH), 5.70–5.62 (m, 4H, O-CH_2_-O), 5.52–5.47 (m, 1H, CH=C), 4.92 (sept., *J* = 6.3 Hz, 2H, CH(CH_3_)_2_), 4.87 (d, *J* = 4.0 Hz, 2H, CH_2_-N), 4.50 (d, *J* = 5.5 Hz, 2H, ArCH_2_NH), 2.76 (dd, *J* = 23.3, 7.9 Hz, 2H, CH_2_-P), 2.18 (t, *J* = 7.7 Hz, 2H, O=CCH_2_(CH_2_)_5_CH_3_), 1.65–1.59 (m, 5H, CH_3_; O=CCH_2_CH_2_(CH_2_)_4_CH_3_), 1.31 (d, *J* = 6.4 Hz, 12H, CH(CH_3_)_2_), 1.24–1.28 (m, 8H, O=CCH_2_CH_2_(CH_2_)_4_CH_3_), 0.86 (t, *J* = 6.9 Hz, 3H, O=C(CH_2_)_6_CH_3_). ^13^C NMR (101 MHz, CDCl_3_) *δ* 173.2 (NHC=O), 153.2 (C=O), 145.1 (C^q,Ar^), 135.6, 135.5 (CH=C), 121.8 (CH^Ar^), 118.2, 118.1 (CH=C), 84.6, 84.1 (O-CH_2_-O), 73.4 (CH(CH_3_)_2_), 57.8, 57.7 (CH_2_-N), 36.6 (ArCH_2_NH), 35.0 (O=CCH_2_(CH_2_)_5_CH_3_), 31.7 (O=CCH_2_CH_2_(CH_2_)_4_CH_3_, 29.3 (O=CCH_2_CH_2_CH_2_(CH_2_)_3_CH_3_), 29.0 (O=C(CH_2_)_3_CH_2_CH_2_CH_2_CH_3_), 27.8 (CH_2_-P), 26.4 (CH_2_-P), 25.6 (O=C(CH_2_)_4_CH_2_CH_2_CH_3_), 22.6 (O=C(CH_2_)_5_CH_2_CH_3_), 21.6, 21.6 (CH(CH_3_)_2_, 14.3, 14.2 (CH_3_), 14.1 (O=C(CH_2_)_6_CH_3_). ^31^P NMR (162 MHz, CDCl_3_) *δ* 27.31. HRMS (ESI): *m*/*z* [M+Na]^+^ calcd for C_26_H_45_N_4_NaO_10_P: 627.276551, found: 627.275980.

[[(*E*)-4-(4-Nitrophenyltriazol-1-yl)-3-methyl-but-2-enyl]-(isopropoxycarbonyloxymethoxy)phosphoryl]oxymethyl isopropyl carbonate (**11l**)

The title compound was prepared from **10** (100 mg, 0.24 mmol) and 1-ethynyl-4-nitrobenzene (45 mg, 0.31 mmol) following the general procedure A; the resulting suspension was stirred for 2 h at 40 °C. After purification on a silica gel column chromatography (EtOAc/PE, 5/5), the desired pure compound **11l** (93 mg, 69%) was obtained as a yellowish oil. ^1^H NMR (400 MHz, CDCl_3_) *δ* 8.28 (d, *J* = 8.5 Hz, 2H, H^Ar^), 8.04 (d, *J* = 8.5 Hz, 2H, H^Ar^), 8.00 (s, 1H, H^5^), 5.73–5.60 (m, 5H, O-CH_2_-O, CH=C), 4.98 (d, *J* = 3.7 Hz, 2H, CH_2_-N), 4.89 (sept., *J* = 6.4 Hz, 2H, CH(CH_3_)_2_), 2.81 (dd, *J* = 23.2, 7.9 Hz, 2H, CH_2_-P), 1.66 (d, *J* = 4.9 Hz, 3H, CH_3_), 1.28 (d, *J* = 6.2 Hz, 12H, CH(CH_3_)_2_). ^13^C NMR (101 MHz, CDCl_3_) *δ* 153.2 (C=O), 147.3 (C^q,Ar^), 146.1 (C^q,Ar^), 137.0 (C^q,Ar^), 135.3, 135.2 (CH=C), 126.2 (CH^Ar^), 124.2 (CH^Ar^), 121.0 (CH^Ar^), 119.1, 119.0 (CH=C), 84.2, 84.1 (O-CH_2_-O), 73.5 (CH(CH_3_)_2_), 58.2, 58.1 (CH_2_-N), 27.8, (CH_2_-P), 26.4 (CH_2_-P), 21.6, 21.5 (CH(CH_3_)_2_), 14.3, 14.3 (CH_3_). ^31^P NMR (162 MHz, CDCl_3_) *δ* 27.26. HRMS (ESI): *m*/*z* [M+H]^+^ calcd for C_23_H_32_N_4_O_11_P: 571.179971, found: 571.179797.

1-[(*E*)-4-[Bis(isopropoxycarbonyloxymethoxy)phosphoryl]-2-methyl-but-2-enyl]triazole-4-carboxylic acid (**11m**)

To a solution of propiolic acid (49 μL, 1.1 equiv., 0.80mmol) and (*E*)-4-Azido-3-methyl-but-2-enyl-*bis*(POC)phosphonate **10** (307 mg, 1.0 equiv., 0.73 mmol) in anhydrous CH_2_Cl_2_ (10 mL) was added 2-nitrophenylboronic acid (25 mg, 0.21 equiv., 0.15 mmol) at room temperature. The solution was stirred for 42 h at room temperature. After evaporation of the solvent, the residue was purified by silica gel chromatography (MeOH/EtOAc, 1/9) to give compound **11m** (203 mg, 57%) as a colorless oil. ^1^H NMR (400 MHz, CD_3_OD) *δ* 8.28 (s, 1H, H^5^), 5.72–5.64 (m, 4H, O-CH_2_-O), 5.57–5.51 (m, 1H, CH=C), 5.06 (d, *J* = 4.4 Hz, 2H, CH_2_-N), 4.94 (sept., *J* = 6.4 Hz, 2H, CH(CH_3_)_2_), 2.91 (dd, *J* = 23.2, 7.9 Hz, 2H, CH_2_-P), 1.67 (d, *J* = 4.8 Hz, 3H, CH_3_), 1.33 (d, *J* = 6.2 Hz, 12H, CH(CH_3_)_2_). ^13^C NMR (101 MHz, CD_3_OD) *δ* 153.2 (C=O), 136.0, 135.8 (CH=C), 126.7 (CH^Ar^), 117.5, 117.3 (CH=C), 84.4, 84.3 (O-CH_2_-O), 73.1 (CH(CH_3_)_2_), 57.2, 57.1 (CH_2_-N), 26.8, 25.4 (CH_2_-P), 20.5 (CH(CH_3_)_2_), 13.6, 13.1 (CH_3_). ^31^P NMR (162 MHz, CD_3_OD) *δ* 27.94. HRMS (ESI): *m*/*z* [M+H]^+^ calcd for C_18_H_29_N_3_O_11_P: 494.153422, found: 494.153404.

Methyl-1-[(*E*)-4-[bis(isopropoxycarbonyloxymethoxy)phosphoryl]-2-methyl-but-2-enyl]triazole-4-carboxylate (**11n**)

The title compound was prepared from **10** (124 mg, 0.29 mmol) and methyl propiolate (34 μL, 0.38 mmol) using the general procedure A; the resulting suspension was stirred for 2 h at 40 °C. After purification on a silica gel column chromatography (EtOAc/PE, 6/4), the desired pure compound **11n** (92 mg, 63%) was obtained as a colorless oil. ^1^H NMR (250 MHz, CD_3_OD) *δ* 8.57 (s, 1H, H^5^), 5.79–5.69 (m, 4H, O-CH_2_-O), 5.65–5.56 (m, 1H, CH=C), 5.14 (d, *J* = 4.2 Hz, 2H, CH_2_-N), 4.99 (sept., *J* = 6.4 Hz, 2H, CH(CH_3_)_2_), 4.00 (s, 3H, OCH_3_), 2.97 (dd, *J* = 23.2, 7.9 Hz, 2H, CH_2_-P), 1.74 (d, *J* = 4.7 Hz, 3H, CH_3_), 1.39 (d, *J* = 6.2 Hz, 12H, CH(CH_3_)_2_). ^13^C NMR (101 MHz, CDCl_3_) *δ* 161.0 (O=C-OMe), 153.1 (C=O), 140.3 (C^q,Ar^), 135.1, 134.9 (CH=C), 127.4 (CH^Ar^), 119.1, 118.9 (CH=C), 84.2, 84.1 (O-CH_2_-O), 73.4 (CH(CH_3_)_2_), 58.0, 57.9 (CH_2_-N), 52.1 (OCH_3_), 27.8, 26.4 (CH_2_-P), 21.6, 21.5 (CH(CH_3_)_2_), 14.3, 14.2 (CH_3_). ^31^P NMR (162 MHz, CDCl_3_) *δ* 27.19. HRMS (ESI): *m*/*z* [M+Na]^+^ calcd for C_19_H_30_N_3_NaO_11_P: 530.151016, found: 530.151002.

Ethyl-1-[(*E*)-4-[bis(isopropoxycarbonyloxymethoxy)phosphoryl]-2-methyl-but-2-enyl]triazole-4-carboxylate (**11o**)

The title compound was prepared from **10** (120 mg, 0.28 mmol) and methyl propiolate (37 μL, 0.37 mmol) using the general procedure A; the resulting suspension was stirred for 2 h at 40 °C. After purification on a silica gel column chromatography (EtOAc/PE, 5/5), the desired pure compound **11o** (94 mg, 64%) was obtained as a colorless oil. ^1^H NMR (400 MHz, CD_3_OD) *δ* 8.53 (s, 1H, H^5^), 5.74–5.66 (m, 4H, O-CH_2_-O), 5.59–5.54 (m, 1H, CH=C), 5.10 (d, *J* = 4.2 Hz, 2H, CH_2_-N), 4.95 (sept., *J* = 6.3 Hz, 2H, CH(CH_3_)_2_), 4.43 (q, *J* = 7.1 Hz, 2H, OCH_2_CH_3_), 2.93 (dd, *J* = 23.2, 7.9 Hz, 2H, CH_2_-P), 1.70 (d, *J* = 4.8 Hz, 3H, CH_3_), 1.42 (t, *J* = 7.1 Hz, 3H, OCH_2_CH_3_), 1.34 (d, *J* = 6.7 Hz, 12H, CH(CH_3_)_2_). ^13^C NMR (101 MHz, CD_3_OD) *δ* 161.5 (O=C-OEt), 153.2 (C=O), 139.7 (C^q,Ar^), 135.8, 135.6 (CH=C), 128.2 (CH^Ar^), 117.9, 117.8 (CH=C), 84.3, 84.2 (O-CH_2_-O), 73.1 (CH(CH_3_)_2_), 60.8 (OCH_2_CH_3_), 57.2, 57.1 (CH_2_-N), 26.8, 25.4 (CH_2_-P), 20.5 (CH(CH_3_)_2_), 13.2 (OCH_2_CH_3_), 13.1, 13.0 (CH_3_). ^31^P NMR (162 MHz, CD_3_OD) *δ* 27.92. HRMS (ESI): *m*/*z* [M+Na]^+^ calcd for C_20_H_32_N_3_NaO_11_P: 544.166666, found: 544.166837.

[[(*E*)-4-(4-Methylcarbamoyltriazol-1-yl)-3-methyl-but-2-enyl]-(isopropoxycarbonyloxymethoxy)phosphoryl]oxymethyl isopropyl carbonate (**11p**)

The title compound was prepared from **10** (121 mg, 0.29 mmol) and *N*-methylprop-2-ynamide (31 mg, 0.37 mmol) following the general procedure A; the resulting suspension was stirred for 2 h at 40 °C. After purification on a silica gel column chromatography (MeOH/CH_2_Cl_2_, 1/99), the desired pure compound **11p** (118 mg, 82%) was obtained as a colorless oil. ^1^H NMR (400 MHz, CD_3_OD) *δ* 8.34 (s, 1H, H^5^), 5.74–5.66 (m, 4H, O-CH_2_-O), 5.58–5.52 (m, 1H, CH=C), 5.09 (d, *J* = 4.0 Hz, 2H, CH_2_-N), 4.96 (sept., *J* = 6.2 Hz, 2H, CH(CH_3_)_2_), 2.98 (s, 3H, NHCH_3_), 2.92 (dd, *J* = 23.2, 7.9 Hz, 2H, CH_2_-P), 1.69 (d, *J* = 4.6 Hz, 3H, CH_3_), 1.35 (d, *J* = 6.3 Hz, 12H, CH(CH_3_)_2_). ^13^C NMR (101 MHz, CD_3_OD) *δ* 161.7 (O=C-NHCH_3_), 153.2 (C=O), 142.9 (C^q,Ar^), 136.0, 135.8 (CH=C), 125.7 (CH^Ar^), 117.5, 117.4 (CH=C), 84.4, 84.3 (O-CH_2_-O), 73.1 (CH(CH_3_)_2_), 57.1, 57.0 (CH_2_-N), 26.8, 25.4 (CH_2_-P), 24.7 (NHCH_3_), 20.5 (CH(CH_3_)_2_), 13.1, 13.1 (CH_3_). ^31^P NMR (162 MHz, CD_3_OD) *δ* 27.91. HRMS (ESI): *m*/*z* [M+Na]^+^ calcd for C_19_H_31_N_4_NaO_10_P: 529.167001, found: 529.166551.

[[(*E*)-4-[4-(Ethylcarbamoyl)triazol-1-yl]-3-methyl-but-2-enyl]-(isopropoxycarbonyloxymethoxy)phosphoryl]oxymethyl isopropyl carbonate (**11q**)

The title compound was prepared from **10** (115 mg, 0.27 mmol) and N-ethylprop-2-ynamide (34 mg, 0.35 mmol) following the general procedure A; the resulting suspension was stirred for 2 h at 40 °C. After purification on a silica gel column chromatography (MeOH/CH_2_Cl_2_, 1/99), the desired pure compound **11q** (128 mg, 91%) was obtained as a colorless oil. ^1^H NMR (400 MHz, CD_3_OD) *δ* 8.35 (s, 1H, H^5^), 5.75–5.67 (m, 4H, O-CH_2_-O), 5.59–5.53 (m, 1H, CH=C), 5.09 (d, *J* = 4.0 Hz, 2H, CH_2_-N), 4.96 (sept., *J* = 6.2 Hz, 2H, CH(CH_3_)_2_), 3.47 (q, *J* = 7.2 Hz, 2H, NHCH_2_CH_3_), 2.93 (dd, *J* = 23.1, 7.9 Hz, 2H, CH_2_-P), 1.70 (d, *J* = 4.7 Hz, 3H, CH_3_), 1.35 (d, *J* = 6.2 Hz, 12H, CH(CH_3_)_2_), 1.28 (t, *J* = 7.2 Hz, 3H, NHCH_2_CH_3_). ^13^C NMR (101 MHz, CD_3_OD) *δ* 160.8 (O=C-NH), 153.2 (C=O), 142.99 (C^q,Ar^), 136.0, 135.8 (CH=C), 125.7 (CH^Ar^), 117.5, 117.4 (CH=C), 84.4, 84.3 (O-CH_2_-O), 73.1 (CH(CH_3_)_2_), 57.1, 57.0 (CH_2_-N), 33.7 (NHCH_2_CH_3_), 26.8, 25.4 (CH_2_-P), 20.5 (CH(CH_3_)_2_), 13.6 (NHCH_2_CH_3_), 13.1, 13.0 (CH_3_). ^31^P NMR (162 MHz, CD_3_OD) *δ* 27.91. HRMS (ESI): *m*/*z* [M+Na]^+^ calcd for C_20_H_33_N_4_NaO_10_P: 543.182651, found: 543.182603.

(*E*)-4-Hydroxy-3-methyl-but-2-enyl-(HDP/POC) phosphonate (**13**)

To a solution of (HDP/POC) allylphosphonate (**12**) (1.00 g, 2.0 equiv., 1.94 mmol) and 2-methyl-2-propen-1-ol (**8**) (81 μL, 1.0 equiv., 0.97 mmol) in dry CH_2_Cl_2_ (10 mL), Hoveyda–Grubbs catalyst (91 mg, 15 mol%, 0.15 mmol) was added. The catalyst addition was performed in five equal portions of 3 mol% (18.20 mg, 0.029 mmol) at t = 0, 3, 6, 9 and 21 h over the course of the reaction. The solution was sonicated at 55 °C under nitrogen atmosphere for 24 h. Volatiles were evaporated and the residue was purified by silica gel column chromatography (EtOAc/PE, 5/5) to give the desired phosphonate derivative **13** (480 mg, 88 %) as brown oil. ^1^H NMR (400 MHz, CDCl_3_) *δ* 5.67–5.59 (m, 2H, O-CH_2_-O), 5.47–5.41 (m, 1H, CH=C), 4.92 (sept., *J* = 6.3 Hz, 1H, CH(CH_3_)_2_), 4.21–4.08 (m, 2H, H^a^), 4.02 (d, *J* = 5.0 Hz, 2H, CH_2_-OH), 3.47 (t, *J* = 6.2 Hz, 2H, H^c^), 3.38 (t, *J* = 6.7 Hz, 2H, CH_3_(CH_2_)_14_CH_2_-O), 2.67 (dd, *J* = 22.5, 7.8 Hz, 2H, CH_2_-P), 1.90 (quint., *J* = 6.3 Hz, 2H, H^b^), 1.69 (dd, *J* = 4.3, 1.3 Hz, 3H, CH_3_), 1.54 (quint., *J* = 7.0 Hz, 2H, CH_3_(CH_2_)_13_CH_2_CH_2_-O), 1.32–1.24 (m, 32H, CH_3_(CH_2_)_13_CH_2_CH_2_-O, CH(CH_3_)_2_), 0.87 (t, *J* = 6.7 Hz, 3H, CH_3_(CH_2_)_15_O). ^13^C NMR (101 MHz, CDCl_3_) *δ* 153.3 (C=O), 140.8, 140.7 (CH=C), 112.6, 112.5 (CH=C), 84.4, 84.3 (O-CH_2_-O), 73.1 (CH(CH_3_)_2_), 71.2 (CH_3_(CH_2_)_14_CH_2_-O), 68.1, 68.0 (CH_2_-OH), 66.5, 66.4 (C^c^), 63.3, 63.2 (C^a^), 31.9, 30.7, 30.6 (C^b^), 29.7, 29.5, 29.4, 27.1 (CH_2_-P), 26.2, 25.7 (CH_2_-P), 22.7, 21.6 (CH(CH_3_)_2_), 14.1 (CH_3_(CH_2_)_15_O), 13.9, 13.8 (CH_3_). ^31^P NMR (162 MHz, CDCl_3_) *δ* 26.71. HRMS (ESI): *m*/*z* [M+H]^+^ calcd for C_29_H_58_O_8_P: 565.386382, found: 565.385868.

(*E*)-4-Azido-3-methyl-but-2-enyl-(HDP/POC) phosphonate (**14**)

To a solution of (*E*)-4-hydroxy-3-methyl-but-2-enyl-(HDP/POC)phosphonate **13** (277 mg, 1.0 equiv., 0.49 mmol) in anhydrous CH_2_Cl_2_ (5 mL) were added dropwise methansulfonyl chloride (42 μL, 1.1 equiv., 0.54 mmol) and anhydrous triethylamine (75 μL, 1.1 equiv., 0.54 mmol) at 0 °C under the argon. After 40 min stirring at room temperature, the mixture was diluted with CH_2_Cl_2_ and washed with water (3 × 10 mL) and brine. The organic layer was dried over MgSO_4_, filtrated and concentrated in vacuo. The residue was directly used for the next step without further purification. To a solution of the above product in anhydrous DMF (8 mL) was added sodium azide (159 mg, 5.0 equiv., 2.45 mmol) under nitrogen atmosphere. After 5 h stirring at room temperature, the mixture was diluted with EtOAc and water and then extracted with EtOAc. The combined organic layer was washed with water (5 × 10 mL), brine, dried over MgSO_4_, and concentrated in vacuo. The residue was purified by silica gel column chromatography (EtOAc/PE, 4/6) to give (*E*)-4-Azido-3-methyl-but-2-enyl-(HDP/POC) phosphonate **14** (242 mg, 84%) as a colorless oil. ^1^H NMR (400 MHz, CDCl_3_) *δ* 5.69–5.58 (m, 2H, O-CH_2_-O), 5.51–5.41 (m, 1H, CH=C), 4.91 (sept., *J* = 6.3 Hz, 1H, CH(CH_3_)_2_), 4.22–4.07 (m, 2H, H^a^), 3.70 (d, *J* = 3.9 Hz, 2H, CH_2_-N_3_), 3.46 (t, *J* = 6.2 Hz, 2H, H^c^), 3.37 (t, *J* = 6.7 Hz, 2H, CH_3_(CH_2_)_14_CH_2_-O), 2.68 (dd, *J* = 22.7, 7.7 Hz, 2H, CH_2_-P), 1.90 (quint., *J* = 6.3 Hz, 2H, H^b^), 1.73 (d, *J* = 4.3 Hz, 3H, CH_3_), 1.53 (quint., *J* = 7.0 Hz, 2H, CH_3_(CH_2_)_13_CH_2_CH_2_-O), 1.32–1.22 (m, 32H, CH_3_(CH_2_)_13_CH_2_CH_2_-O, CH(CH_3_)_2_), 0.86 (t, *J* = 6.7 Hz, 3H, CH_3_(CH_2_)_15_O). ^13^C NMR (100 MHz, CDCl_3_) *δ* 153.3 (C=O), 135.4, 135.3 (CH=C), 117.3, 117.2 (CH=C), 84.5, 84.4 (O-CH_2_-O), 73.1 (CH(CH_3_)_2_), 71.2 (CH_3_(CH_2_)_14_CH_2_-O), 66.5 (C^c^), 63.5, 63.4 (C^a^), 58.7, 58.6 (CH_2_-N_3_), 32.0, 30.8, 30.7 (C^b^), 29.6 (CH_2_-P), 26.1, 26.0 (CH_2_-P), 22.7, 21.6 (CH(CH_3_)_2_), 14.8, 14.7 (CH=CCH_3_), 14.1 (CH_3_(CH_2_)_15_O). ^31^P NMR (162 MHz, CDCl_3_) *δ* 27.80. IR *ν*_max_ (neat, cm^−1^): 2922.60, 2851.80, 2094.55, 1755.94, 1460.42, 1257.26, 1100.26, 1047.93, 992.52, 949.43 cm^−1^. HRMS (ESI): *m*/*z* [M+H]^+^ calcd for C_29_H_57_N_3_O_7_P: 590.392864, found: 590.393411.

General Procedure B for Huisgen 1,3-dipolar cycloaddition

To a solution of terminal alkyne (1.3 equiv.) and (*E*)-4-Azido-3-methyl-but-2-enyl-(HDP/POC) phosphonate **14** (1.0 equiv.) in *t*BuOH/H_2_O (2:1) were added sodium ascorbate (0.6 equiv.) and CuSO_4_.5H_2_O (0.1 equiv.). The resulting suspension was stirred at 40 °C until completion (followed by TLC), then the crude mixture was co-evaporated five times with methanol. The residue was purified by silica gel column chromatography using an elution gradient of petroleum ether/ethyl acetate to give the desired HDP/POC phosphonate derivatives.

[3-Hexadecyloxypropoxy-[(*E*)-3-methyl-4-[4-(4-propylphenyl]triazol-1-yl]but-2-enyl]phosphoryl]oxymethyl isopropyl carbonate (**15a**)

The title compound was prepared from **14** (123 mg, 0.21 mmol) and 1-ethynyl-4-propylbenzene (43 μL mg, 0.27 mmol) following the general procedure B; the resulting suspension was stirred for 2 h at 40 °C. After purification on a silica gel column chromatography (EtOAc/PE, 4/6), the desired pure compound **15a** (104 mg, 68%) was obtained as a colorless oil. ^1^H NMR (400 MHz, CDCl_3_) δ 7.75 (s, 1H, H^5^), 7.73 (s, 2H, H^Ar^), 7.22 (d, *J* = 8.0 Hz, 2H, H^Ar^), 5.70 ‒ 5.56 (m, 3H, O-CH_2_-O; 1H, CH=C), 4.94 (s, 2H, CH_2_-N), 4.89 (sept., *J* = 6.3 Hz, 1H, CH(CH_3_)_2_), 4.22 ‒ 4.11 (m, 2H, H^a^), 3.44 (t, *J* = 6.1 Hz, 2H, H^c^), 3.35 (t, *J* = 6.7 Hz, 2H, CH_3_(CH_2_)_14_CH_2_-O), 2.73 (dd, *J* = 22.8, 7.8 Hz, 2H, CH_2_-P), 2.60 (t, *J* = 7.5 Hz, 2H, Ar-CH_2_CH_2_CH_3_), 1.90 (quint., *J* = 6.3 Hz, 2H, H^b^), 1.68–1.63 (m, 3H, CH_3_; 2H, CH_2_CH_2_CH_3_), 1.52 (quint., *J* = 7.0 Hz, 2H, CH_3_(CH_2_)_13_CH_2_CH_2_-O), 1.29–1.25 (m, 32H, CH_3_(CH_2_)_13_CH_2_CH_2_-O, CH(CH_3_)_2_), 0.95 (t, *J* = 7.3 Hz, 3H, CH_2_CH_2_CH_3_), 0.88 (t, *J* = 7.0 Hz, 3H, CH_3_(CH_2_)_15_O). ^13^C NMR (101 MHz, CDCl_3_) *δ* 153.3 (C=O), 148.3 (C^q,Ar^), 142.8 (C^q,Ar^), 128.9 (CH^Ar^), 128.0 (CH=C), 125.6 (CH^Ar^), 118.9 (CH=C), 118.8 (CH^Ar^), 84.4 (O-CH_2_-O), 73.2 (CH(CH_3_)_2_), 71.2 (CH_3_(CH_2_)_14_CH_2_-O), 66.4 (C^c^), 63.5 (C^a^), 57.9 (CH_2_-N), 37.8 (ArCH_2_CH_2_CH_3_), 31.9, 30.7 (C^b^), 29.6 (CH_2_-P), 24.5 (ArCH_2_CH_2_CH_3_), 22.7, 21.6, 21.5 (CH(CH_3_)_2_), 14.3 (C=CCH_3_), 14.1 (CH_3_(CH_2_)_15_O), 13.7 (ArCH_2_CH_2_CH_3_). ^31^P NMR (162 MHz, CDCl_3_) *δ* 27.46. HRMS (ESI): *m*/*z* [M+H]^+^ calcd for C_40_H_69_N_3_NaO_7_P: 734.486765, found: 734.486600.

[3-Hexadecyloxypropoxy-[(*E*)-3-methyl-4-[4-(4-pentylphenyl)triazol-1-yl]but-2-enyl]phosphoryl]oxymethyl isopropyl carbonate (**15c**)

The title compound was prepared from **14** (93 mg, 0.16mmol) and 1-ethynyl-4-penthylbenzene (40 μL, 0.21 mmol) following the general procedure B; the resulting suspension was stirred for 2 h at 40 °C. After purification on a silica gel column chromatography (EtOAc/PE, 4/6), the desired pure compound **15c** (89 mg, 74%) was obtained as a colorless oil. ^1^H NMR (400 MHz, CDCl_3_) *δ* 7.74 (d, *J* = 3.8 Hz, 2H, H^Ar^), 7.73 (s, 1H, H^5^), 7.22 (d, *J* = 8.0 Hz, 2H, H^Ar^), 5.70 ‒ 5.56 (m, 3H, O-CH_2_-O; 1H, CH=C), 4.94 (s, 2H, CH_2_-N), 4.90 (sept., *J* = 6.2 Hz, 1H, CH(CH_3_)_2_), 4.22–4.11 (m, 2H, H^a^), 3.44 (t, *J* = 6.1 Hz, 2H, H^c^), 3.35 (t, *J* = 6.7 Hz, 2H, CH_3_(CH_2_)_14_CH_2_-O), 2.73 (dd, *J* = 22.8, 7.8 Hz, 2H, CH_2_-P), 2.62 (t, *J* = 7.8 Hz, 2H, Ar-CH_2_(CH_2_)_3_CH_3_), 1.90 (quint., *J* = 6.3 Hz, 2H, H^b^), 1.65–1.61 (m, 5H, CH_3_, Ar-CH_2_CH_2_CH_2_CH_2_CH_3_), 1.52 (quint., *J* = 7.0 Hz, 2H, CH_3_(CH_2_)_13_CH_2_CH_2_-O), 1.29–1.25 (m, 36H, CH_3_(CH_2_)_13_CH_2_CH_2_-O, CH(CH_3_)_2,_ Ar-CH_2_CH_2_CH_2_CH_2_CH_3_), 0.88 (t, *J* = 6.9 Hz, 6H, Ar-(CH_2_)_4_CH_3_, CH_3_(CH_2_)_15_O). ^13^C NMR (101 MHz, CDCl_3_) *δ* 153.2 (C=O), 148.4, 148.3 (C^q,Ar^), 143.1 (C^q,Ar^), 135.2, 135.0 (C^q,Ar^), 128.8 (CH^Ar^), 127.9 (CH=C), 125.6 (CH^Ar^), 118.9 (CH=C), 118.8 (CH^Ar^), 84.4 (O-CH_2_-O), 73.2 (CH(CH_3_)_2_), 71.2 (CH_3_(CH_2_)_14_CH_2_-O), 66.4 (C^c^), 63.6 (C^a^), 57.8 (CH_2_-N), 35.7 (Ar-CH_2_(CH_2_)_3_CH_3_), 31.9, 31.5, 31.1, 30.8, 30.7 (C^b^), 29.8, 29.7, 29.6, 29.5, 29.4, 26.2 (CH_2_-P), 22.7, 22.5, 21.6, 21.5 (CH(CH_3_)_2_), 14.3, 14.2 (C=CCH_3_), 14.1 (CH_3_(CH_2_)_15_O), 14.0 (Ar-(CH_2_)_4_CH_3_). ^31^P NMR (162 MHz, CDCl_3_) *δ* 27.56. HRMS (ESI): *m*/*z* [M+Na]^+^ calcd for C_42_H_72_N_3_NaO_7_P: 784.500009, found: 784.499917.

[3-Hexadecyloxypropoxy-[(*E*)-3-methyl-4-[4-(heptylphenyl)triazol-1-yl]but-2-enyl]phosphoryl]oxymethyl isopropyl carbonate (**15e**)

The title compound was prepared from **14** (113 mg, 0.19 mmol) and 1-ethynyl-4-heptylbenzene (56 μL, 0.25 mmol) following the general procedure B; the resulting suspension was stirred for 2 h at 40 °C. After purification on a silica gel column chromatography (EtOAc/PE, 4/6), the desired pure compound **15e** (118 mg, 78%) was obtained as a colorless oil. ^1^H NMR (400 MHz, CDCl_3_) *δ* 7.73 (t, *J* = 3.9 Hz, 3H, H^Ar^, H^5^), 7.22 (d, *J* = 8.0 Hz, 2H, H^Ar^), 5.70–5.60 (m, 3H, O-CH_2_-O; 1H, CH=C), 4.94 (s, 2H, CH_2_-N), 4.89 (sept., *J* = 6.3 Hz, 1H, CH(CH_3_)_2_), 4.20–4.11 (m, 2H, H^a^), 3.43 (t, *J* = 6.1 Hz, 2H, H^c^), 3.35 (t, *J* = 6.7 Hz, 2H, CH_3_(CH_2_)_14_CH_2_-O), 2.73 (dd, *J* = 22.8, 7.8 Hz, 2H, CH_2_-P), 2.63 (t, *J* = 7.8 Hz, 2H, Ar-CH_2_(CH_2_)_5_CH_3_), 1.90 (quint., *J* = 6.3 Hz, 2H, H^b^), 1.65 (d, *J* = 4.1 Hz, 3H, C=CCH_3_), 1.60 (quint., *J* = 7.2, Ar-CH_2_CH_2_(CH_2_)_4_CH_3_), 1.52 (quint., *J* = 7.0 Hz, 2H, CH_3_(CH_2_)_13_CH_2_CH_2_-O), 1.29–1.25 (m, 40H, CH_3_(CH_2_)_13_CH_2_CH_2_-O, CH(CH_3_)_2,_ Ar-CH_2_CH_2_(CH_2_)_4_CH_3_), 0.93 (t, *J* = 7.3 Hz, 3H, Ar-(CH_2_)_6_CH_3_), 0.88 (t, *J* = 6.7 Hz, 3H, CH_3_(CH_2_)_15_O). ^13^C NMR (101 MHz, CDCl_3_) *δ* 153.3 (C=O), 143.5 (C^q,Ar^), 135.2 (C^q,Ar^), 135.1 (C^q,Ar^), 129.5, 129.3 ((CH^Ar^), 128.8 (CH^Ar^), 127.9 (CH=C), 125.6 (CH^Ar^), 118.9 (CH=C), 84.5, 84.4 (O-CH_2_-O), 73.3 (CH(CH_3_)_2_), 71.2 (CH_3_(CH_2_)_14_CH_2_-O), 66.4 (C^c^), 63.6, 63.5 (C^a^), 57.8 (CH_2_-N), 35.4 (Ar-CH_2_(CH_2_)_5_CH_3_), 33.5, 32.0, 31.4, 30.8, 30.7 (C^b^), 30.2, 29.7, 29.7, 29.6, 29.6, 29.5, 29.4, 27.6, 26.2 (CH_2_-P), 22.7, 22.3, 21.6, 21.6 (CH(CH_3_)_2_), 14.3 (C=CCH_3_), 14.1 (CH_3_(CH_2_)_15_O), 13.9 (Ar-(CH_2_)_6_CH_3_). ^31^P NMR (162 MHz, CDCl_3_) *δ* 27.49. HRMS (ESI): *m*/*z* [M+H]^+^ calcd for C_44_H_77_N_3_O_7_P: 790.549365, found: 790.549322.

[[(*E*)-4-(4-Carbamoyltriazol-1-yl)-3-methyl-but-2-enyl]-(3-hexadecyloxypropoxy)phosphoryl]oxymethyl isopropyl carbonate (**15j**)

The title compound was prepared from **14** (166 mg, 0.28 mmol) and propiolamide (25 mg, 0.37 mmol) following the general procedure B; the resulting suspension was stirred for 18 h at 40 °C. After purification on a silica gel column chromatography (MeOH/CH_2_Cl_2_, 3/97), the desired pure compound **15j** (130 mg, 70%) was obtained as a colorless oil. ^1^H NMR (400 MHz, CDCl_3_) δ 8.07 (s, 1H, H^5^), 7.05 (s, 1H, NH_2_), 5.76 (s, 1H, NH_2_), 5.69–5.58 (m, 2H, O-CH_2_-O; 1H, CH=C), 4.94 ‒ 4.91 (m, 2H, CH_2_-N; 1H, CH(CH_3_)_2_), 4.22–4.12 (m, 2H, H^a^), 3.46 (t, *J* = 6.1 Hz, 2H, H^c^), 3.37 (t, *J* = 6.7 Hz, 2H, CH_3_(CH_2_)_14_CH_2_-O), 2.71 (dd, *J* = 22.9, 7.8 Hz, 2H, CH_2_-P), 1.90 (quint., *J* = 6.3 Hz, 2H, H^b^), 1.62 (d, *J* = 4.4 Hz, 3H, C=CCH_3_), 1.52 (quint., *J* = 7.0 Hz, 2H, CH_3_(CH_2_)_13_CH_2_CH_2_-O), 1.31–1.24 (m, 32H, CH_3_(CH_2_)_13_CH_2_CH_2_-O, CH(CH_3_)_2_), 0.87 (t, *J* = 7.0 Hz, 3H, CH_3_(CH_2_)_15_O). ^13^C NMR (101 MHz, CDCl_3_) *δ* 161.8 (NH_2_C=O), 153.2 (C=O), 143.1 (C^q,Ar^), 134.2, 134.1 (CH=C), 125.6 (CH^Ar^), 120.1, 120.0 (CH=C), 84.5, 84.4 (O-CH_2_-O), 73.2 (CH(CH_3_)_2_), 71.3 (CH_3_(CH_2_)_14_CH_2_-O), 66.4 (C^c^), 63.6, 63.5 (C^a^), 58.2, 58.1 (CH_2_-N), 31.9, 30.8, 30.7 (C^b^), 29.7, 29.6, 29.6, 29.6, 29.5, 29.3, 27.6, 26.2, 26.1 (CH_2_-P), 22.7, 21.6 (CH(CH_3_)_2_), 14.3, 14.2 (C=CCH_3_), 14.1 (CH_3_(CH_2_)_15_O). ^31^P NMR (162 MHz, CDCl_3_) *δ* 27.11. HRMS (ESI): *m*/*z* [M+H]^+^ calcd for C_32_H_60_N_4_O_8_P: 659.414328, found: 659.414346.

[3-Hexadecyloxypropoxy-[(*E*)-3-methyl-4-[4-(nitrophenyl)triazol-1-yl]but-2-enyl]phosphoryl]oxymethyl isopropyl carbonate (**15l)**

The title compound was prepared from **14** (115 mg, 0.20 mmol) and 1-ethynyl-4-nitrobenzene (37 mg, 0.25 mmol) following the general procedure B; the resulting suspension was stirred for 3 h at 40 °C. After purification on a silica gel column chromatography (EtOAc/PE, 5/5), the desired pure compound **15l** (102 mg, 71%) was obtained as a colorless oil. ^1^H NMR (400 MHz, CDCl_3_) δ 8.27 (d, *J* = 8.8 Hz, 2H, H^Ar^), 8.01 (d, *J* = 8.9 Hz, 2H, H^Ar^), 7.98 (s, 1H, H^5^), 5.70 ‒ 5.58 (m, 3H, O-CH_2_-O; 1H, CH=C), 4.97 (s, 2H, CH_2_-N), 4.89 (sept., *J* = 6.3 Hz, 1H, CH(CH_3_)_2_), 4.22–4.11 (m, 2H, H^a^), 3.44 (t, *J* = 6.2 Hz, 2H, H^c^), 3.35 (t, *J* = 6.6 Hz, 2H, CH_3_(CH_2_)_14_CH_2_-O), 2.73 (dd, *J* = 22.8, 7.8 Hz, 2H, CH_2_-P), 1.90 (quint., *J* = 6.2 Hz, 2H, H^b^), 1.66 (d, *J* = 4.4 Hz, 3H, C=CCH_3_), 1.52 (quint., *J* = 7.0 Hz, 2H, CH_3_(CH_2_)_13_CH_2_CH_2_-O), 1.27–1.23 (m, 32H, CH_3_(CH_2_)_13_CH_2_CH_2_-O, CH(CH_3_)_2_), 0.86 (t, *J* = 7.0 Hz, 3H, CH_3_(CH_2_)_15_O). ^13^C NMR (101 MHz, CDCl_3_) *δ* 153.2 (C=O), 147.3 (C^q,Ar^), 146.0 (C^q,Ar^), 136.9 (C^q,Ar^), 134.7, 134.5 (CH=C), 126.1 (CH^Ar^), 124.3 (CH^Ar^), 121.0 (CH^Ar^), 119.8, 119.7 (CH=C), 84.4, 84.3 (O-CH_2_-O), 73.3, 73.1 (CH(CH_3_)_2_), 71.2 (CH_3_(CH_2_)_14_CH_2_-O), 66.5, 66.3 (C^c^), 63.6, 63.5 (C^a^), 58.1, 58.0 (CH_2_-N), 31.9, 30.8, 30.7 (C^b^), 29.7, 29.6, 29.6, 29.6, 29.5, 29.5, 29.3, 27.5, 26.1, 26.1 (CH_2_-P), 22.7, 21.6, 21.6, 21.6 (CH(CH_3_)_2_), 14.4, 14.3 (C=CCH_3_), 14.1 (CH_3_(CH_2_)_15_O). ^31^P NMR (162 MHz, CDCl_3_) *δ* 27.31. HRMS (ESI): *m*/*z* [M+Na]^+^ calcd for C_37_H_61_N_4_NaO_9_P: 759.406837, found: 759.406817.

## 4. Conclusions

In summary, we have efficiently synthesized various hitherto unknown ANP prodrugs bearing the (*E*)-2′-methyl-but-2′-enyl aliphatic side-chain with 1,4-substituted-1,2,3-triazoles as nucleobase. The convergent synthesis was based on olefin acyclic cross-metathesis and CuAAC cross-coupling reaction as the key steps. All those compounds were evaluated against HBV, HIV and SARS-CoV-2 viruses for their antiviral properties. Among them, compound **15j**, a HDP/POC prodrug with R = C(O)NH_2_, a ribavirin analog, showed 62% inhibition (at 10 μM) without significant cytotoxicity (IC_50_ 66.4 μM in HepG2 cells and IC_50_ = 43.1 μM in HepG2 cells). Further structural optimization of both the (E)-2′-methyl-but-2′-enyl aliphatic side-chain and the heterocycle is underway, alongside more detailed biological testing of the most active compound, with the aim of improving further its antiviral potency.

## Figures and Tables

**Table 1:**
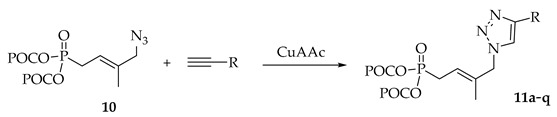
Copper-catalyzed azide alkyne cycloaddition to triazolo compounds **11a**–**q**.

Entry	R	Product	Yield (%)
1 ^a^	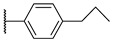	**11a**	83
2 ^a^	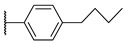	**11b**	88
3 ^a^	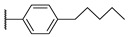	**11c**	85
4 ^a^	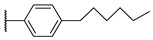	**11d**	84
5 ^a^	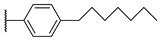	**11e**	91
6 ^a^	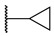	**11f**	79
7 ^a^	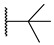	**11g**	48
8 ^a^	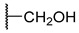	**11h**	71
9 ^a^	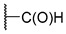	**11i**	21
10 ^a^	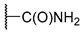	**11j**	78
11 ^a^	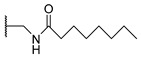	**11k**	94
12 ^a^	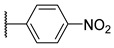	**11l**	69
13 ^b^	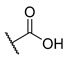	**11m**	57
14 ^a^	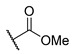	**11n**	63
15 ^a^	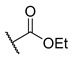	**11o**	64
16 ^a^	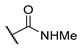	**11p**	82
17 ^a^	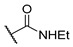	**11q**	91

^a^*Method A:* CuSO_4_.5H_2_O, sodium ascorbate, *t*BuOH/H_2_O (2:1), 40 °C, time (followed by thin layer chromatography (TLC)); ^b^ *Method B:* 2-nitrophenylboronic acid, CH_2_Cl_2_, rt, 42 h.

**Table 2:**
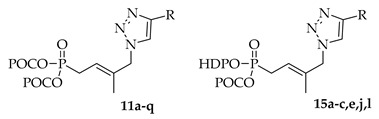
Antiviral activity and cytotoxicity of synthesized compounds in cellular assays at 10 μM.

	HBV			SARS-CoV-2	HIV	MTS	
	% Inhibition		% Cell Viability	% Inhibition	% Inhibition	Cytotoxicity (IC_50_, μM)	
**Cmpd**	Huh7	HepAD38	Huh7	Vero E6	PBM	PBM	HepG2
**11a**	51	19.6	96	<1	<1	50.2	18.3
**11b**	25	14.4	88	<1	<1	19.4	16.2
**11c**	42	<1	89	<1	44.4	47.8	51.8
**11d**	39	38.8	100	<1	41.8	23.7	59.6
**11e**	50	33.5	95	<1	29.9	9.2	90.8
**11f**	<1	36.3	100	<1	30.7	25.5	>100
**11g**	10	17.3	79	<1	12.3	28.3	>100
**11h**	28	15.1	100	12	<1	76.4	>100
**11i**	33	6.1	95	<1	28.4	75.1	>100
**11j**	40	31.3	100	<1	<1	76.8	>100
**11k**	<1	6.1	100	5	42	51.5	57.3
**11l**	40	42.6	100	10	38.8	23.5	85.3
**11m**	12	41.3	57	<1	12.9	>100	>100
**11n**	28	3.9	60	<1	<1	40.9	>100
**11o**	<1	41.2	60	<1	22.4	67.5	>100
**11p**	14	<1	54	<1	36.4	78.3	>100
**11q**	<1	<1	90	<1	31.2	80.6	>100
**15a**	<1	ND	95	<1	ND	ND	ND
**15c**	14	28.8	47	<1	28.2	50.5	>100
**15e**	8	ND	100	<1	ND	ND	ND
**15j**	62	<1	74	<1	32.2	43.1	66.4
**15l**	12	ND	100	<1	ND	ND	ND
**Entecavir**	92	-	96	-	-	-	-
**Remdesivir**	-	-	-	100	-	-	-

ND, not determined; PBM, peripheral blood mononuclear; cmpd, compound.

## Supplementary Materials

The following are available online at https://www.mdpi.com/1420-3049/26/5/1493/s1, 1H-,13C-, 31P- and NOESY NMR spectra of all synthetized molecules.

## Sample Availability

Samples of the compounds are available on request from the corresponding authors.

## References

[1] Krečmerová M., Magel F.D. (2012). Nucleoside and nucleotide analogues for the treatment of herpesvirus infections: Current stage and new prospects in the field of acyclic nucleoside phosphonates. Herpesviridae–A Look into This Unique Family of Viruses.

[2] Holý A. (2005). Current Protocols in Nucleic Acid Chemistry.

[3] De Clercq E., Holý A. (2005). Acyclic nucleoside phosphonates: A key class of antiviral drugs. Nat. Rev. Drug Discov..

[4] De Clercq E. (2011). Acyclic nucleoside phosphonates: An unfinished story. Collect. Czech. Chem. Commun..

[5] Wiemer A.J., Wiemer D.F. (2015). Prodrugs of phosphonates and phosphates: Crossing the membrane barrier. Top. Curr. Chem..

[6] Farquhar D., Srivastva D.N., Kattesch N.J., Saunders P.P. (1983). Biologically reversible phosphate-protective groups. J. Pharm. Sci..

[7] Srivastva D.N., Farquhar D. (1984). Bioreversible phosphate protective groups—Synthesis and stability of model acyloxymethyl phosphates. Bioorg. Chem..

[8] Naesens L., Bischofberger N., Augustijns P., Annaert P., Van den Mooter G., Arimilli M.N., Kim C.U., De Clercq E. (1998). Antiretroviral efficacy and pharmacokinetics of oral bis(isopropyloxycarbonyloxymethyl)-9-(2-phosphonylmethoxypropyl)adenine in mice. Antimicrob. Agents Chemother..

[9] Hostetler K.Y. (2009). Alkoxyalkyl prodrugs of acyclic nucleoside phosphonates enhance oral antiviral activity and reduce toxicity: Current state of the art. Antiviral. Res..

[10] Ballatore C., McGuigan C., De Clercq E., Balzarini J. (2001). Synthesis and evaluation of novel amidate prodrugs of PMEA and PMPA. Bioorg. Med. Chem. Lett..

[11] McGuigan C., Hassan-Abdallah A., Srinivasan S., Wang Y., Siddiqui A., Daluge S.M., Gudmundsson K.S., Zhou H., McLean E.W., Peckham J.P. (2006). Application of phosphoramidate ProTide technology significantly improves antiviral potency of carbocyclic adenosine derivatives. J. Med. Chem..

[12] Topalis D., Pradère U., Roy V., Caillat C., Azouzi A., Broggi J., Snoeck R., Andrei G., Lin J., Eriksson S. (2011). Novel antiviral C5-substituted pyrimidine acyclic nucleoside phosphonates selected as human thymidylate kinase substrates. J. Med. Chem..

[13] Montagu A., Pradere U., Roy V., Nolan S.P., Agrofoglio L.A. (2011). Expeditious convergent procedure for the preparation of bis(POC) prodrugs of new (E)-4-phosphono-but-2-en-1-yl nucleosides. Tetrahedron.

[14] Pradère U., Clavier H., Roy V., Nolan S.P., Agrofoglio L.A. (2011). The shortest strategy for generating phosphonate prodrugs by olefin cross metathesis–application to acyclonucleoside phosphonates. Eur. J. Org. Chem..

[15] Hamada M., Roy V., McBrayer T.R., Whitaker T., Urbina-Blanco C., Nolan S.P., Balzarini J., Snoeck R., Andrei G., Schinazi R.F. (2013). Synthesis and broad spectrum antiviral evaluation of bis(POM) prodrugs of novel acyclic nucleosides. Eur. J. Med. Chem..

[16] Amblard F., Cho J.H., Schinazi R.F. (2009). The Cu(I)-catalyzed Huisgen azide-alkyne 1,3-dipolar cycloaddition reaction in nucleoside, nucleotide and oligonucleotide chemistry. Chem. Rev..

[17] Bessières M., Hervin V., Roy V., Chartier A., Snoeck R., Andrei G., Lohier J.-F., Agrofoglio L.A. (2018). Highly convergent synthesis and antiviral activity of €-but-2-enyl nucleoside phosphonoamidates. Eur. J. Med. Chem..

[18] Tornøe C.W., Christensen C., Meldal M. (2002). Peptidotriazoles on solid phase: [1,2,3]-triazoles by regiospecific copper(i)-catalyzed 1,3-dipolar cycloadditions of terminal alkynes to azides. J. Org. Chem..

[19] Rostovtsev V.V., Green L.G., Fokin V.V., Sharpless K.B. (2002). A Stepwise Huisgen Cycloaddition Process: Copper(I)—Catalyzed Regioselective “Ligation” of Azides and Terminal Alkynes. Angew. Chem. Int. Ed..

[20] Zheng H., McDonald R., Hall D.G. (2010). Boronic acid catalysis for mild and selective [3+2] dipolar cycloadditions to unsaturated carboxylic acids. Chem. Eur. J..

[21] Langley D.R., Walsh A.W., Baldick C.J., Eggers B.J., Rose R.E., Levine S.M., Kapur J., Colonno R.J., Tenney D.J. (2007). Inhibition of hepatitis B virus polymerase by entecavir. J. Virol..

[22] Marlet J., Lier C., Roch E., Maugey M., Moreau A., Combe B., Lefeuvre S., d’Alteroche L., Barbereau D., Causse X. (2020). Revisiting HBV resistance to entecavir with a phenotypic approach. Antiviral Res..

[23] Ladner S.K., Otto M.J., Barker C.S., Zaifert K., Wang G.H., Guo J.T., Seeger C., King R.W. (1997). Inducible expression of human hepatitis B virus (HBV) in stably transfected hepatoblastoma cells: A novel system for screening potential inhibitors of HBV replication. Antimicrob. Agents Chemother..

[24] Stuyver L.J., Lostia S., Adams M., Mathew J.S., Pai B.S., Grier J., Tharnish P.M., Choi Y., Chong Y., Choo H. (2002). Antiviral activities and cellular toxicities of modified 2’,3’-dideoxy-2’,3’-didehydrocytidine analogues. Antimicrob. Agents Chemother..

[25] Schinazi R.F., Sommadossi J.P., Saalmann V., Cannon D.L., Xie M.-W., Hart G.C., Smith G.A., Hahn E.F. (1990). Activity of 3’-azido-3’-deoxythymidine nucleotide dimers in primary lymphocytes infected with human immunodeficiency virus type 1. Antimicrob. Agents Chemother..

[26] Schinazi R.F., McMillan A., Cannon D., Mathis R., Lloyd R.M., Peck A., Sommadossi J.-P., St Clair M., Wilson J., Furman P.A. (1992). Selective inhibition of human immunodeficiency viruses by racemates and enantiomers of *cis*-5-fluoro-1-[2-(hydroxymethyl)-1,3-oxathiolan-5-yl]cytosine. Antimicrob. Agents Chemother..

[27] Belen’kii M.S., Schinazi R.F. (1994). Multiple drug effect analysis with confidence interval. Antivir. Res..

[28] Chou T.-C., Talalay P. (1984). Quantitative analysis of dose-effect relationships: The combined effects of multiple drugs or enzyme inhibitors. Adv. Enzyme Regul..

