# Supplementary material for: Synthesis and Antiviral Evaluation of (1,4-Disubstituted-1,2,3-Triazol)-(E)-2-Methyl-but-2-Enyl Nucleoside Phosphonate Prodrugs
Source: Molecules. 2021 Mar 9;26(5):1493. doi: 10.3390/molecules26051493 (PMC7967160; doi:10.3390/molecules26051493)

Synthesis and antiviral evaluation of (1,4-disubstituted-1,2,3-triazol)-*(E)*-2-methyl-but-2-enyl nucleosides phosphonate prodrugs

Tuniyazi Abuduaini,[1] Vincent Roy,[1,] Julien Marlet,[2] Catherine Gaudy-Graffin,[2] Denys Brand,[2] Cécile Baronti,[3] Franck Touret,[3] Bruno Coutard,[3] Tamara R. McBrayer,[4] Raymond F. Schinazi,[4] and Luigi A. Agrofoglio[1,*]**

[1]Univ. Orléans, CNRS, ICOA, UMR 7311, F-45067, Orléans, France
[2]Inserm U1259, Université de Tours, 37032 Tours, France
[3]Unité des Virus Émergents (UVE: Aix-Marseille Univ - IRD 190 - Inserm 1207 - IHU Méditerranée Infection), Marseille, France.
[4]Center for AIDS Research, Laboratory of Biochemical Pharmacology, Department of Pediatrics, Emory University School of Medicine and Children's Healthcare of Atlanta, Atlanta, Georgia, USA

Corresponding authors : vincent.roy@univ-orleans.fr; luigi.agrofoglio@univ-orleans.fr

Supplementary Informations

Copies of ^{1}H-, ^{13}C- and ^{1}H-^{13}C HMBC NMR spectra of representative molecules

^1H NMR (400 MHz, $CDCl_3$) Analysis of Compound 9

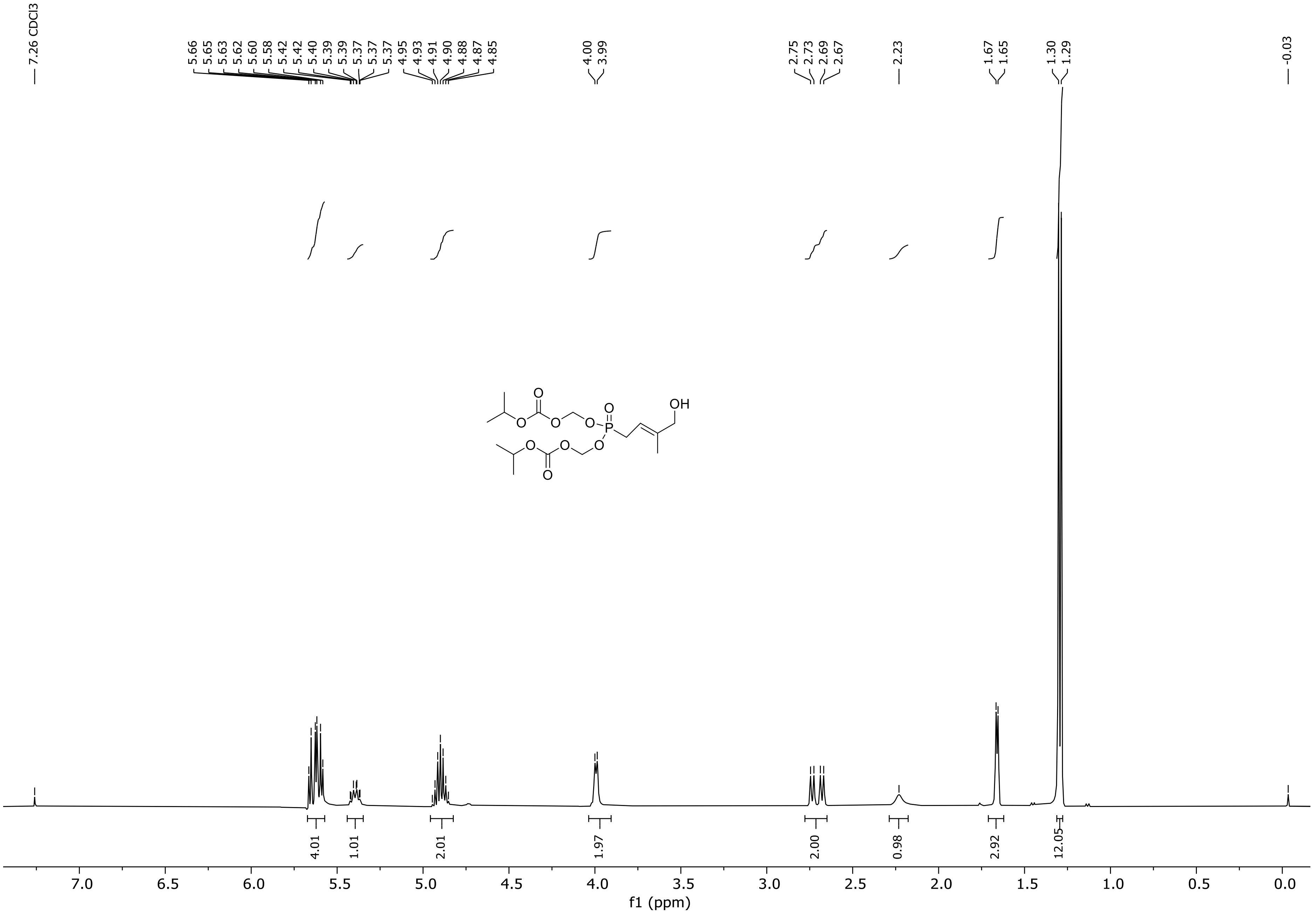

^{13}C NMR (101 MHz, $CDCl_3$) Analysis of Compound 9

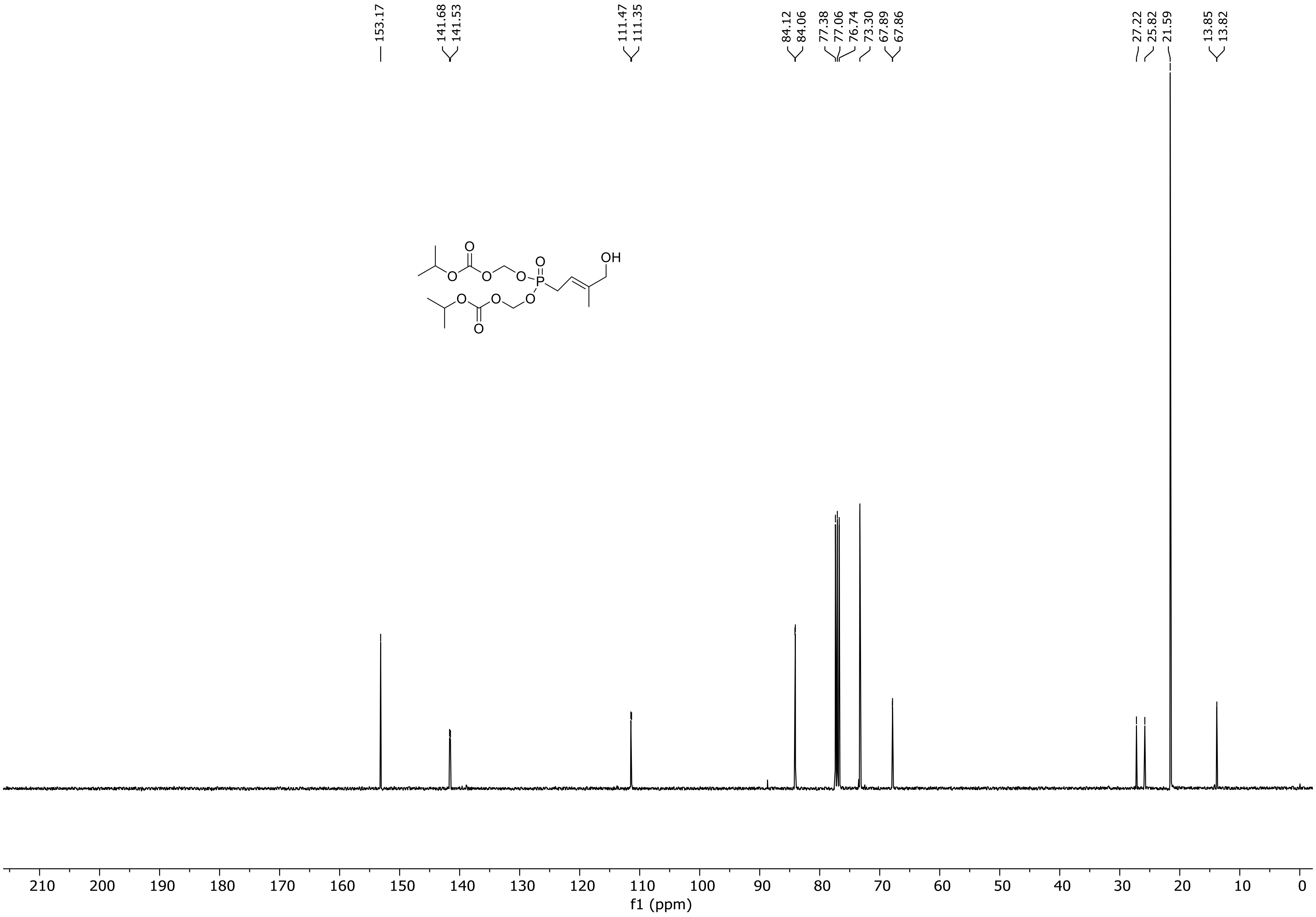

NOESY of Compound 9

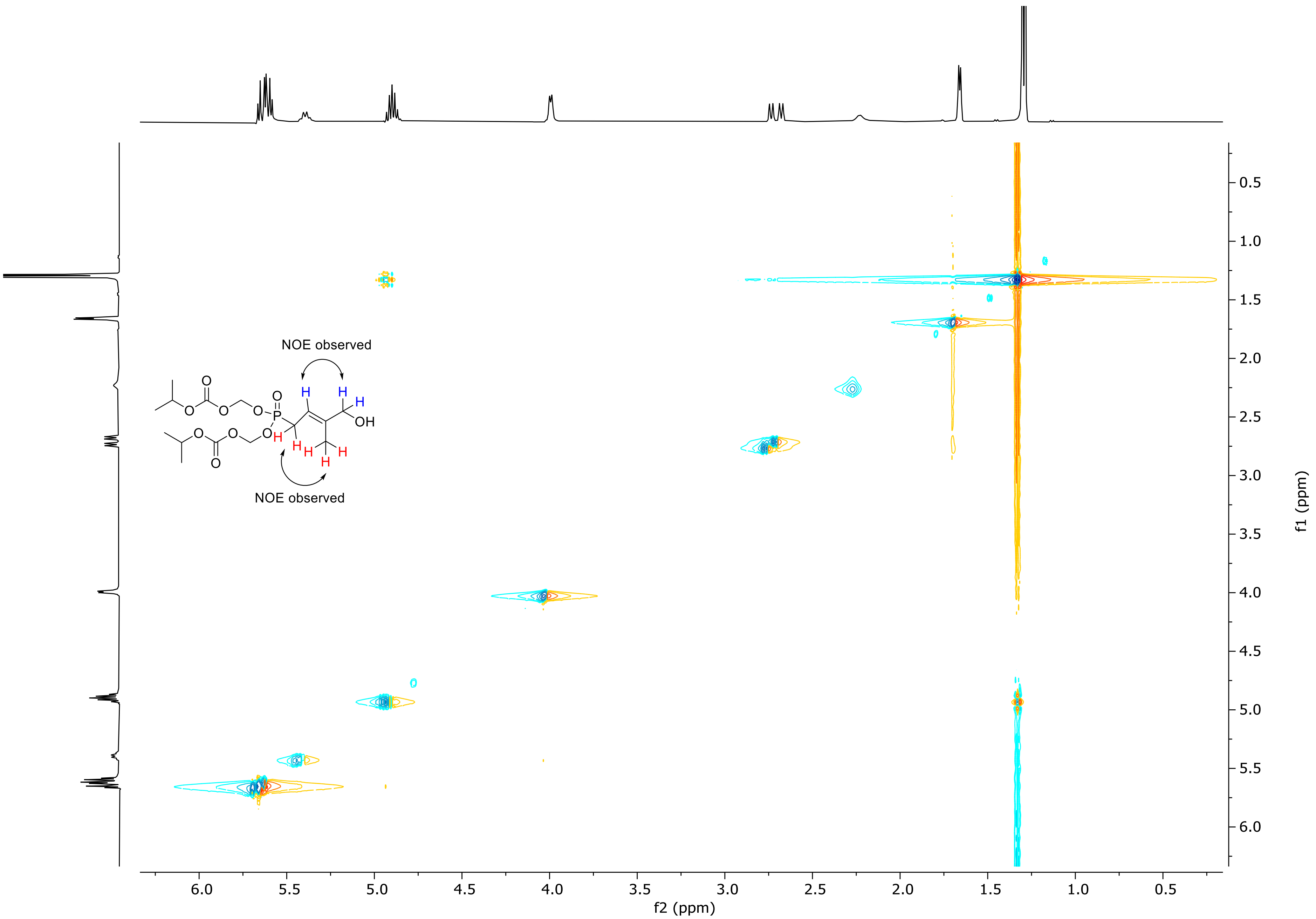

^{31}P NMR (161 MHz, $CDCl_3$) Analysis of Compound 9

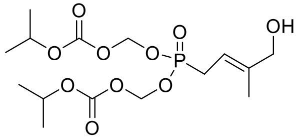

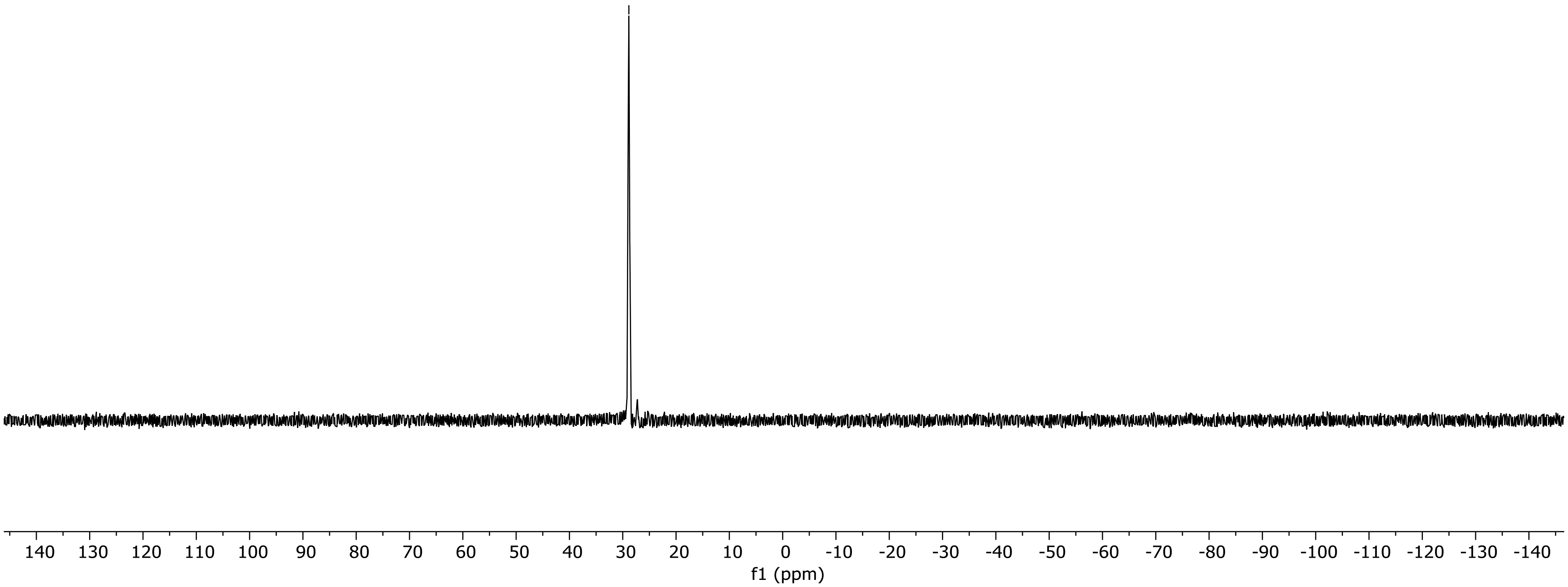

^{1}H NMR (400 MHz, $CDCl_3$) Analysis of Compound 10

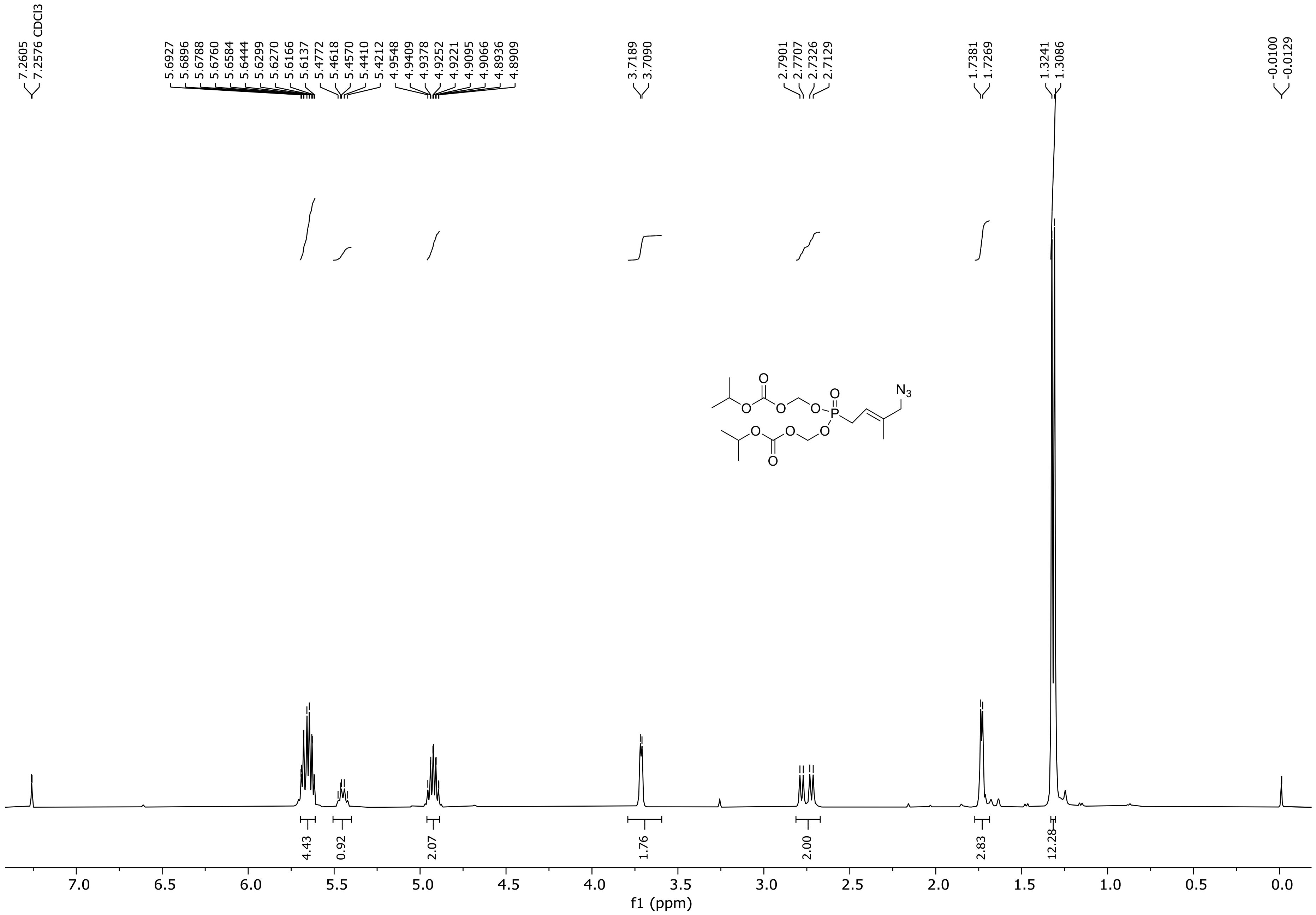

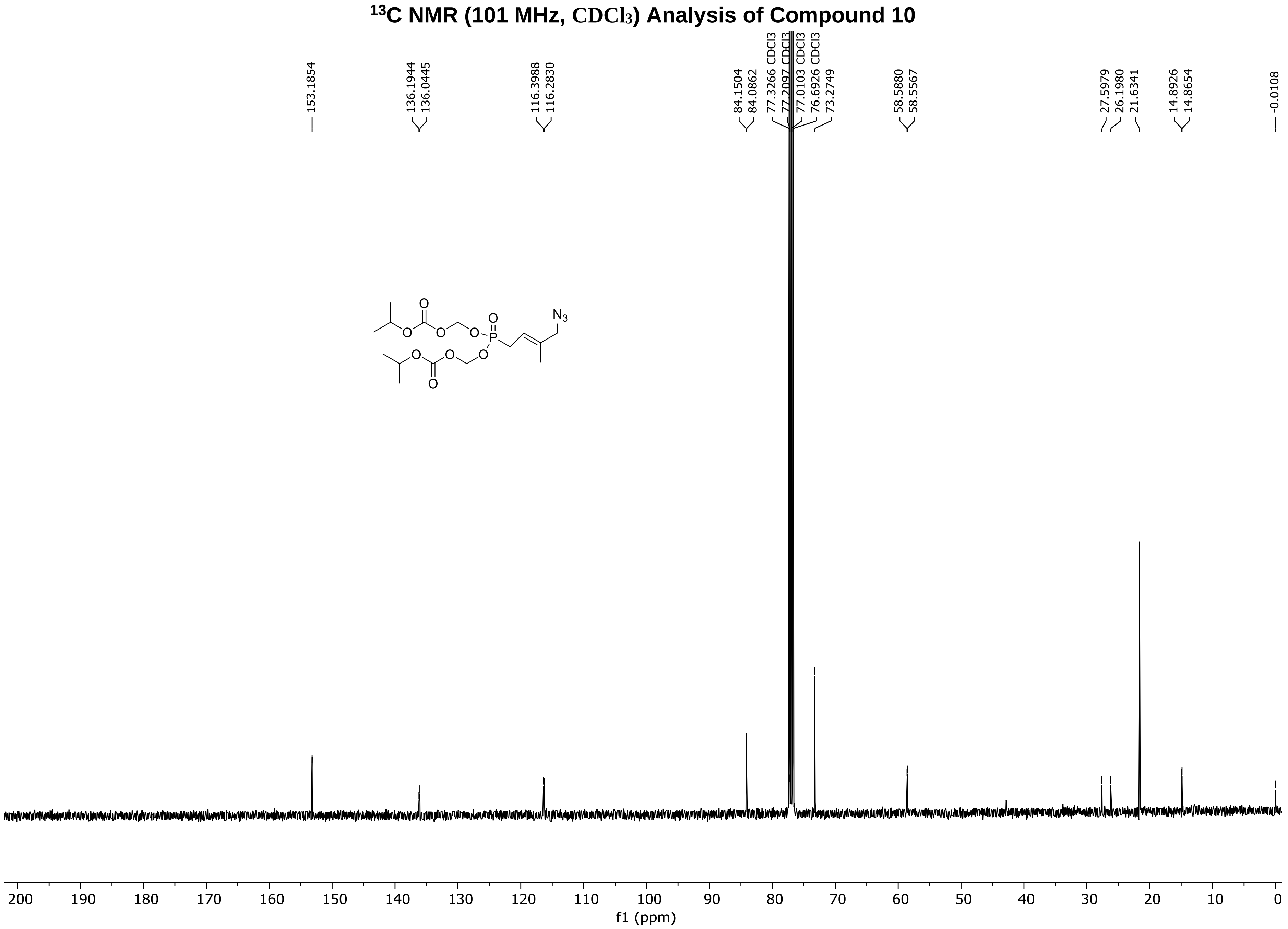
^{13}C NMR (101 MHz, CDCl₃) Analysis of Compound 10
153.1854
136.1944
136.0445
116.3988
116.2830
84.1504
84.0862
77.3266 CDCl3
77.2097 CDCl3
77.0103 CDCl3
76.6926 CDCl3
73.2749
58.5880
58.5567
27.5979
26.1980
21.6341
14.8926
14.8654
-0.0108
f1 (ppm)

^{31}P NMR (161 MHz, $CDCl_3$) Analysis of Compound 10

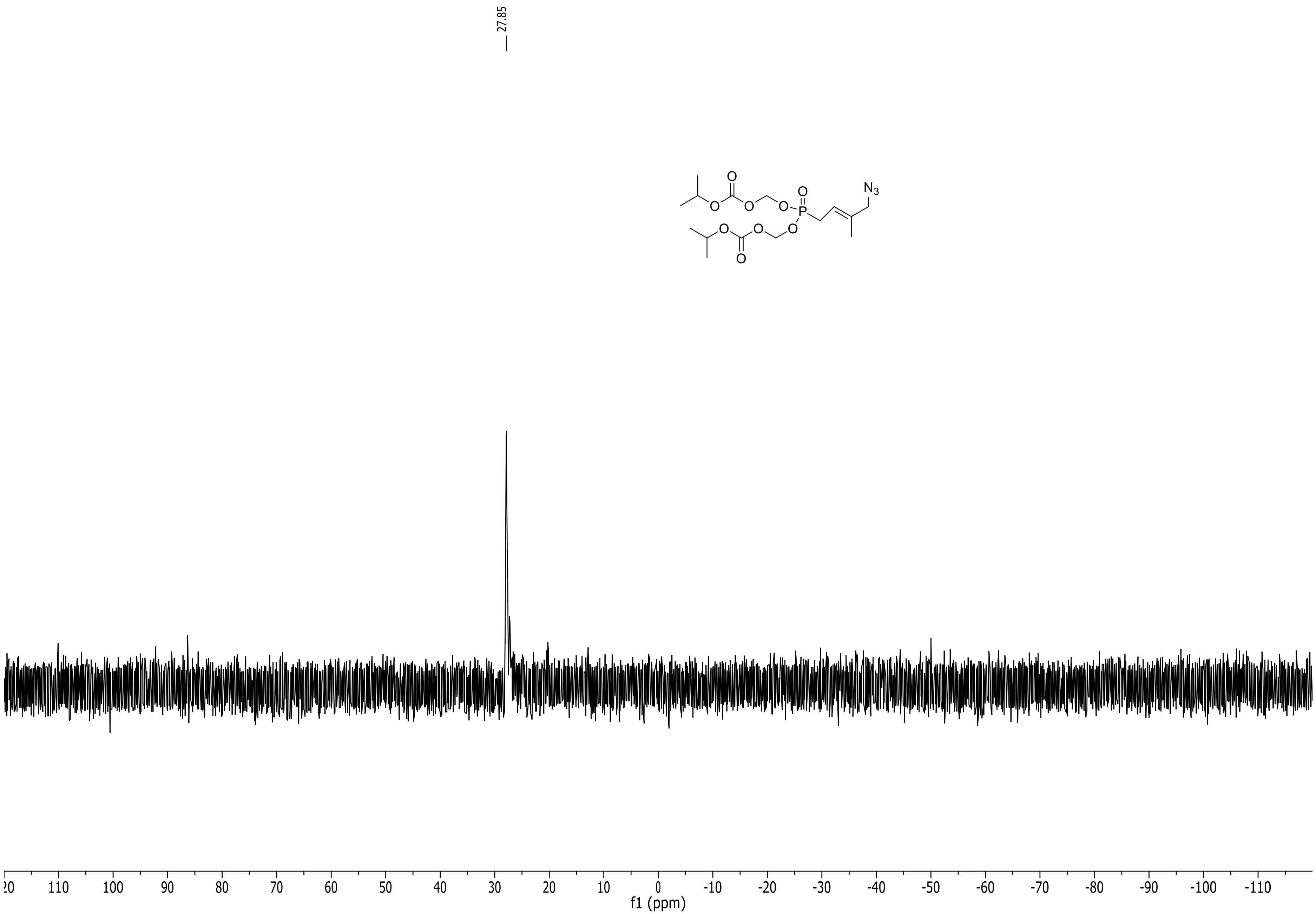

^{1}H NMR (250 MHz, $CDCl_3$) Analysis of Compound 11a

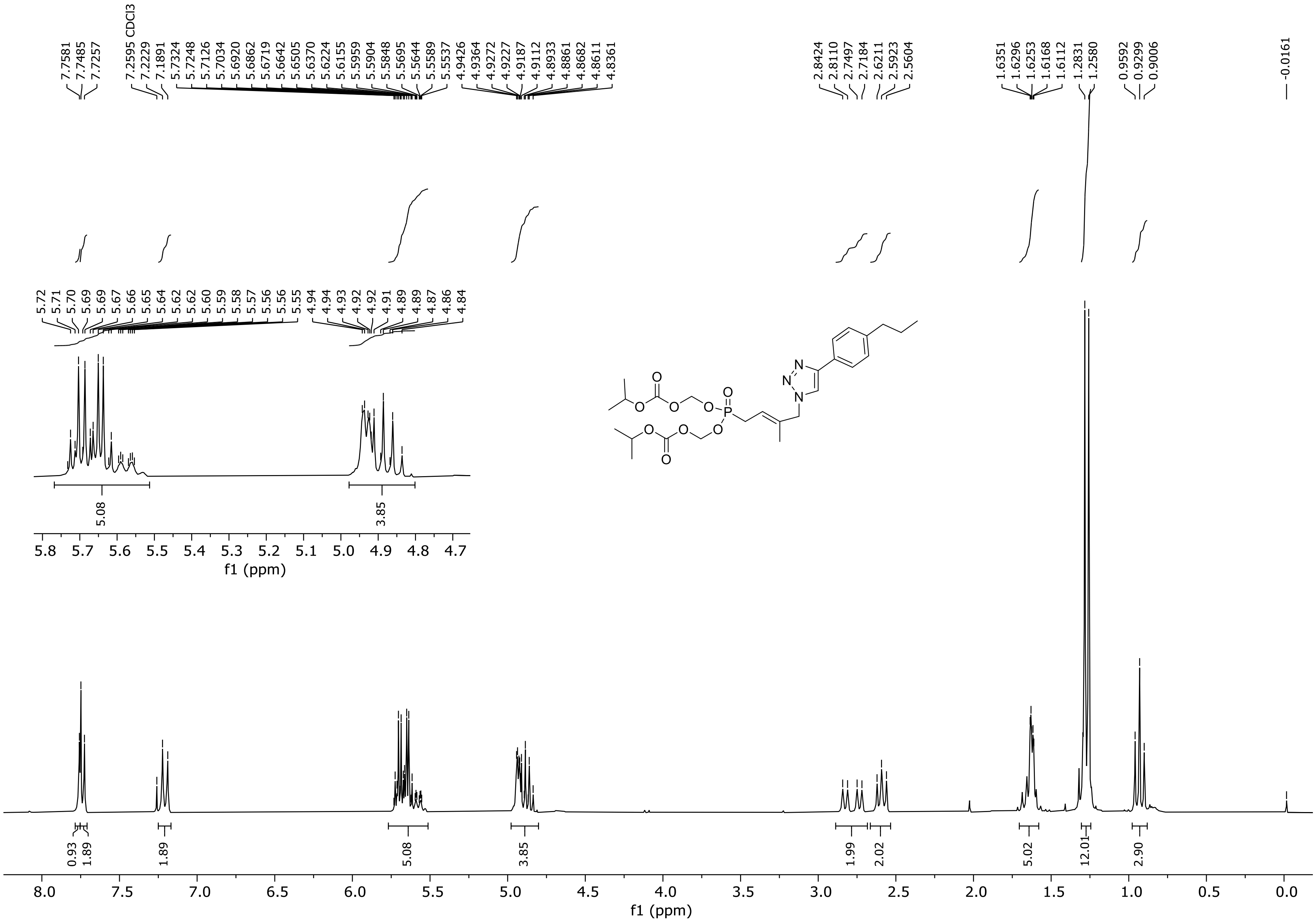

^{13}C NMR (101 MHz, $CDCl_3$) Analysis of Compound 11a

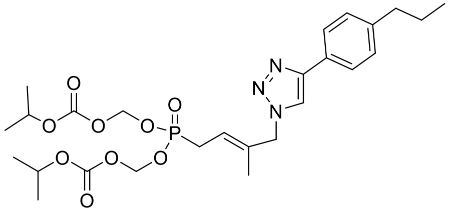

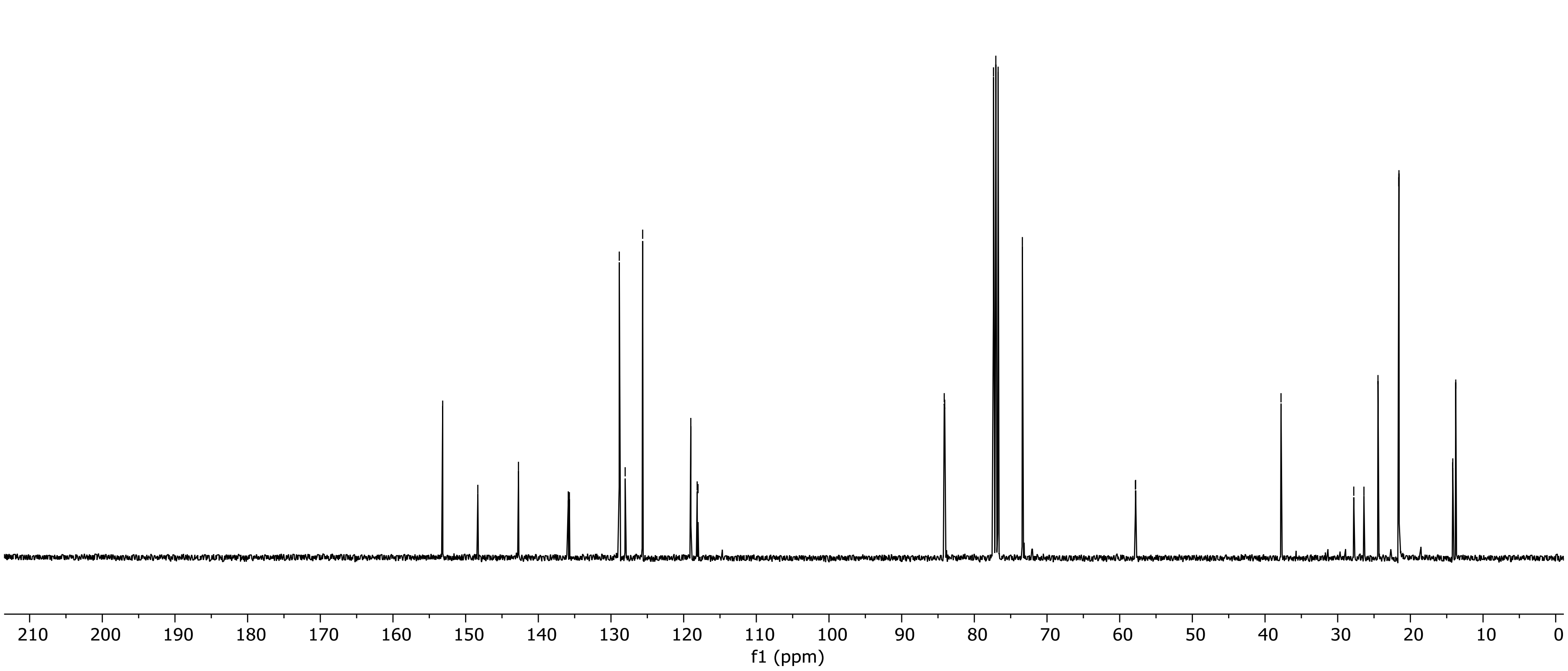

^{31}P NMR (161 MHz, $CDCl_3$) Analysis of Compound 11a

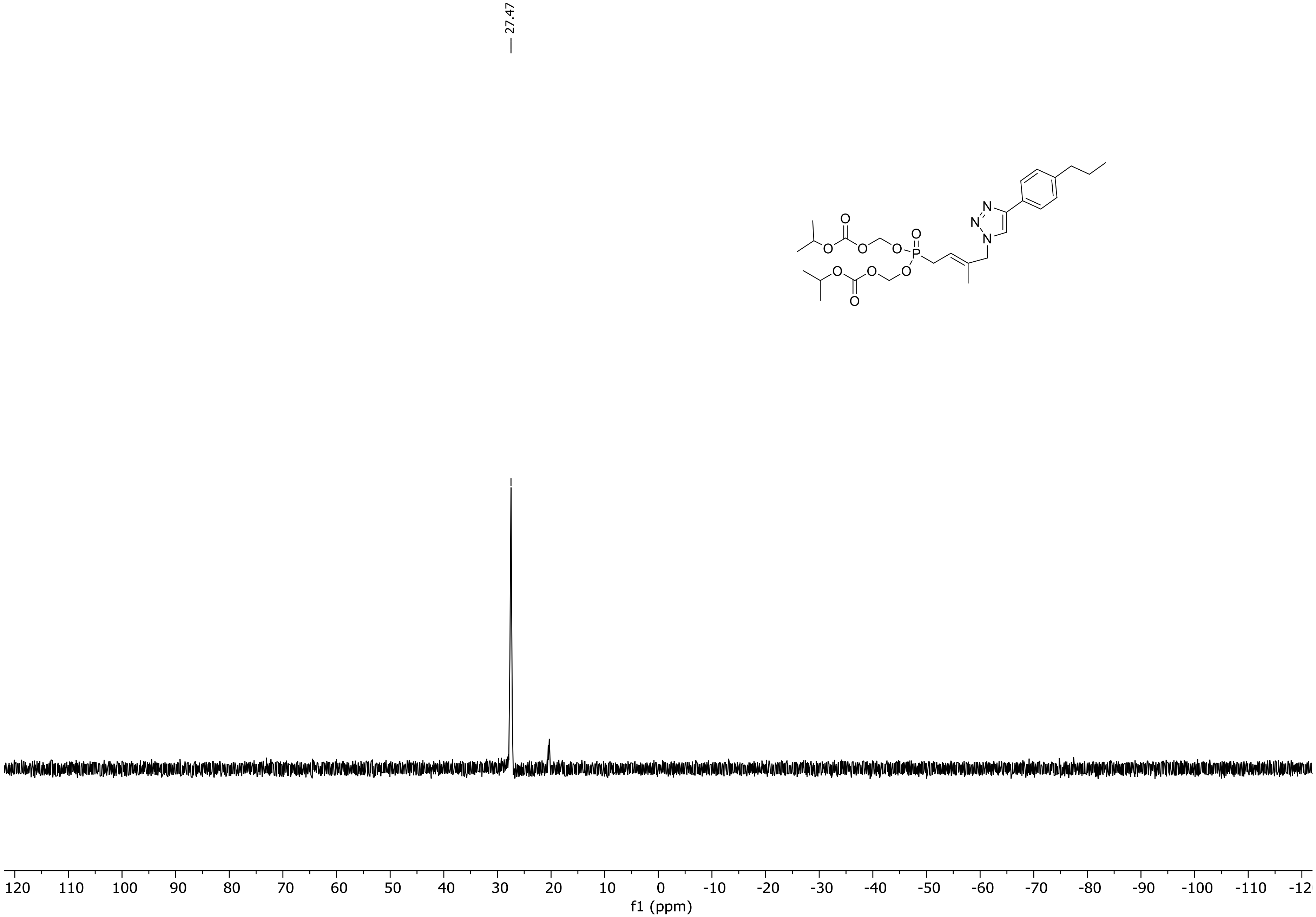

^{1}H NMR (250 MHz, $CDCl_3$) Analysis of Compound 11b

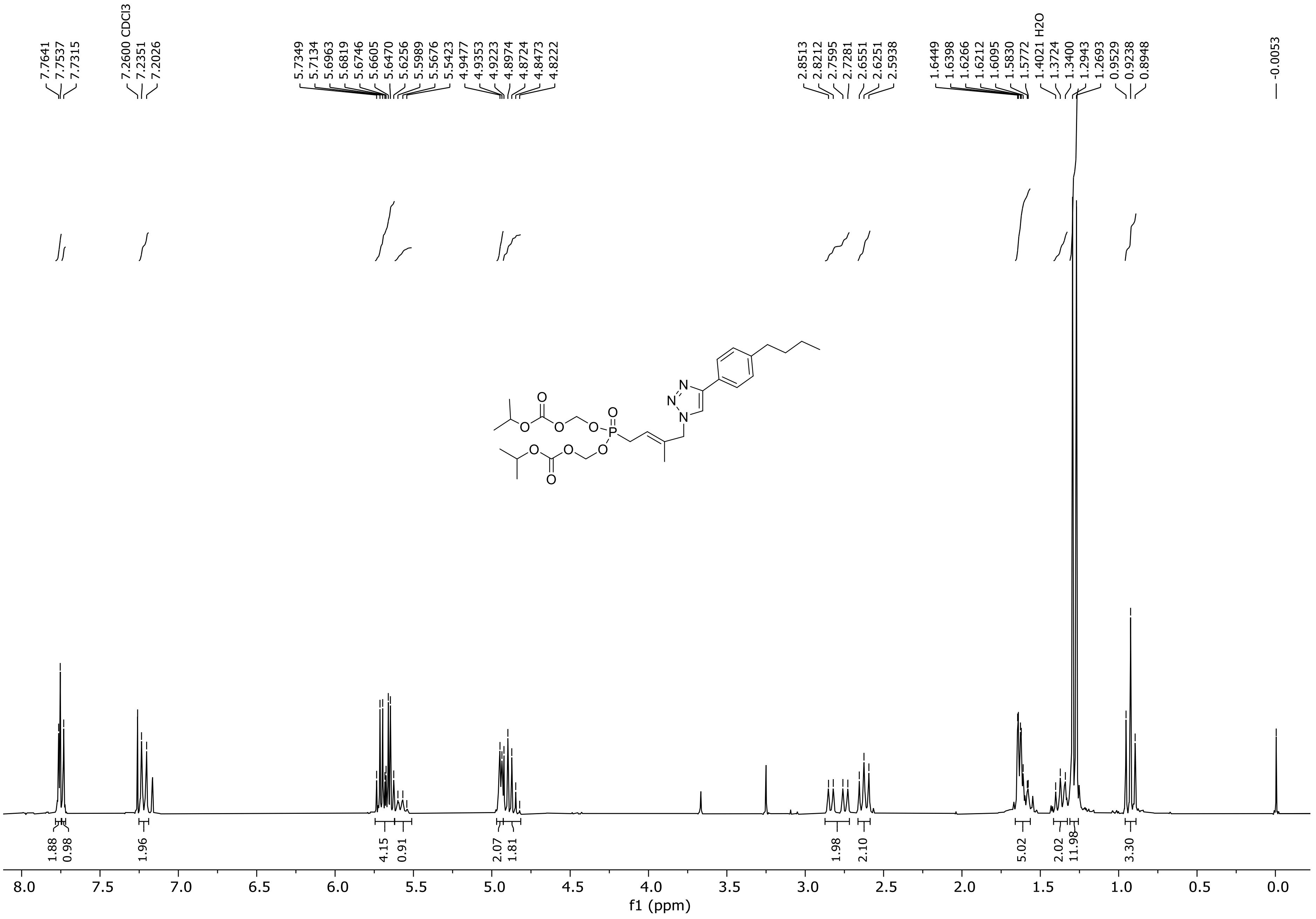

^{13}C NMR (101 MHz, $CDCl_3$) Analysis of Compound 11b

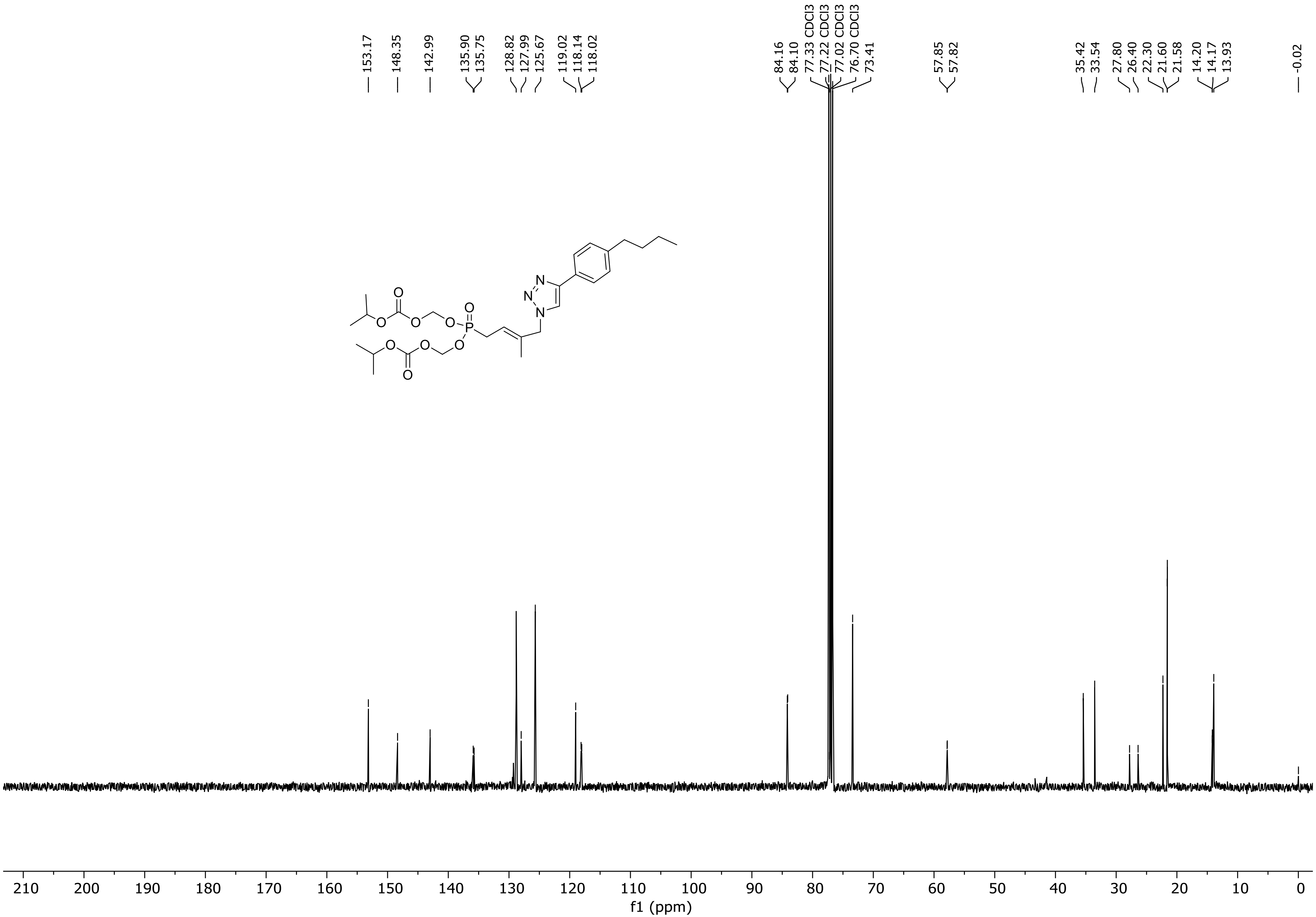

^{31}P NMR (161 MHz, $CDCl_3$) Analysis of Compound 11b

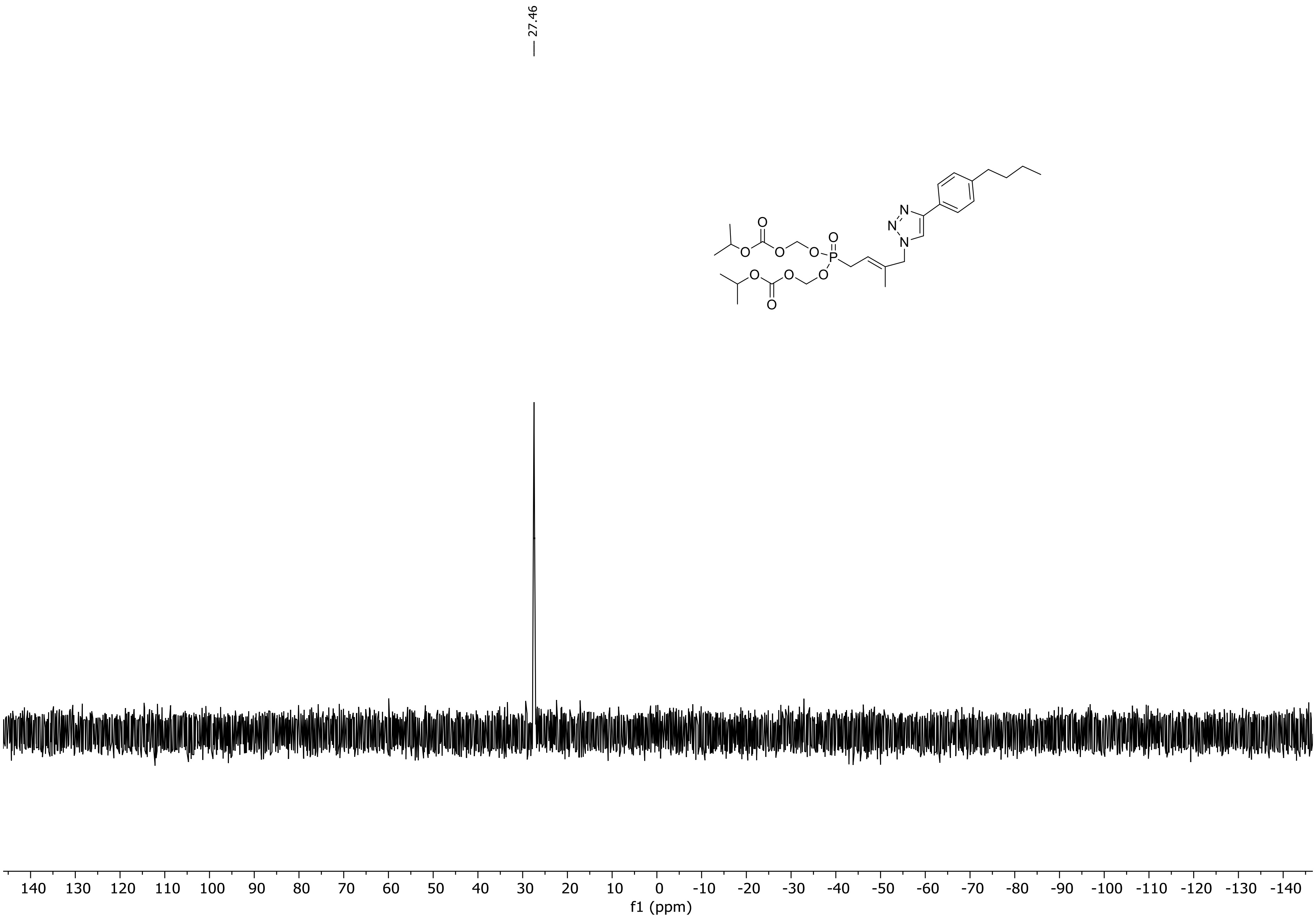

^{1}H NMR (400 MHz, $CDCl_3$) Analysis of Compound 11c

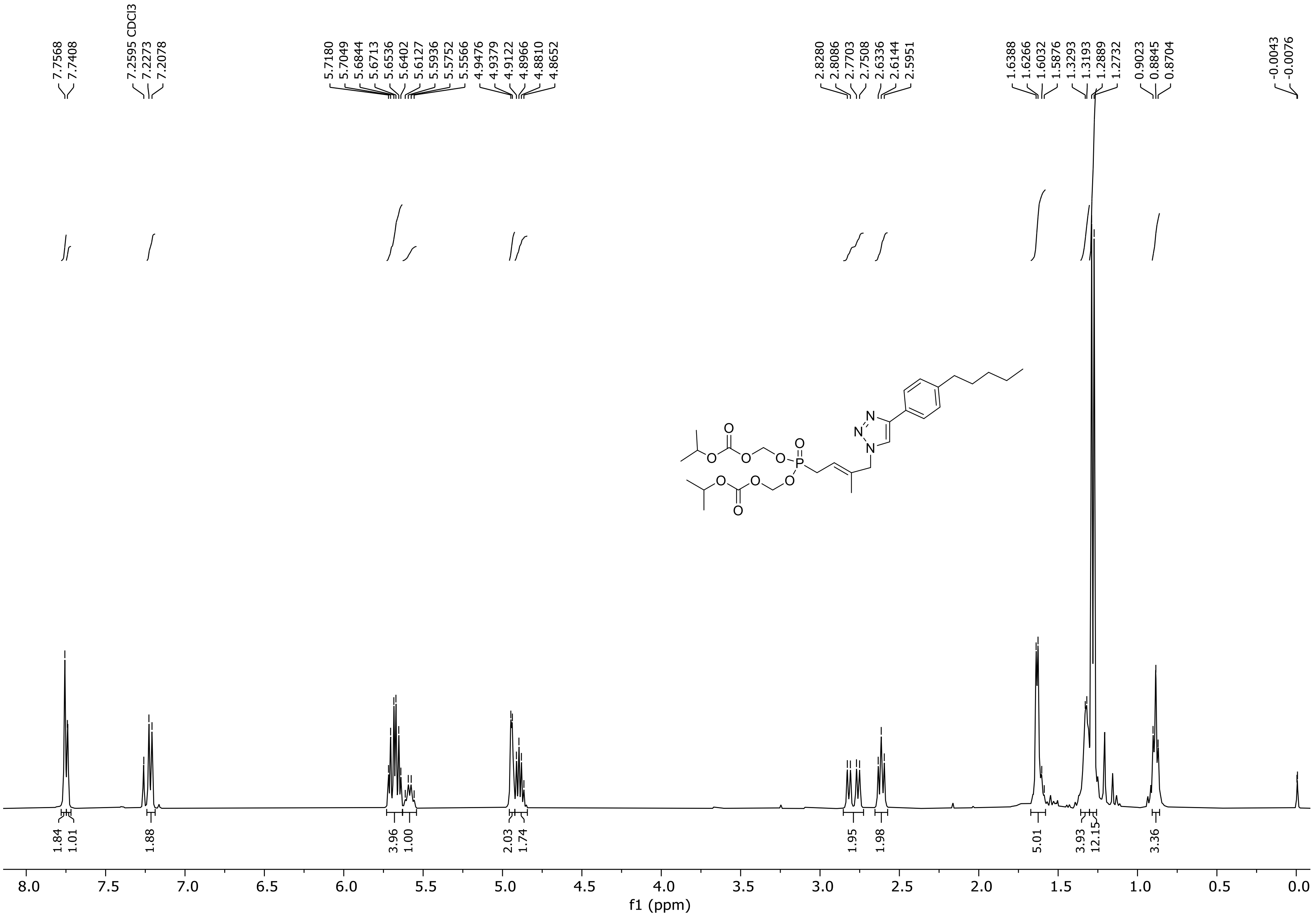

^{13}C NMR (101 MHz, CDCl$_3$) Analysis of Compound 11c

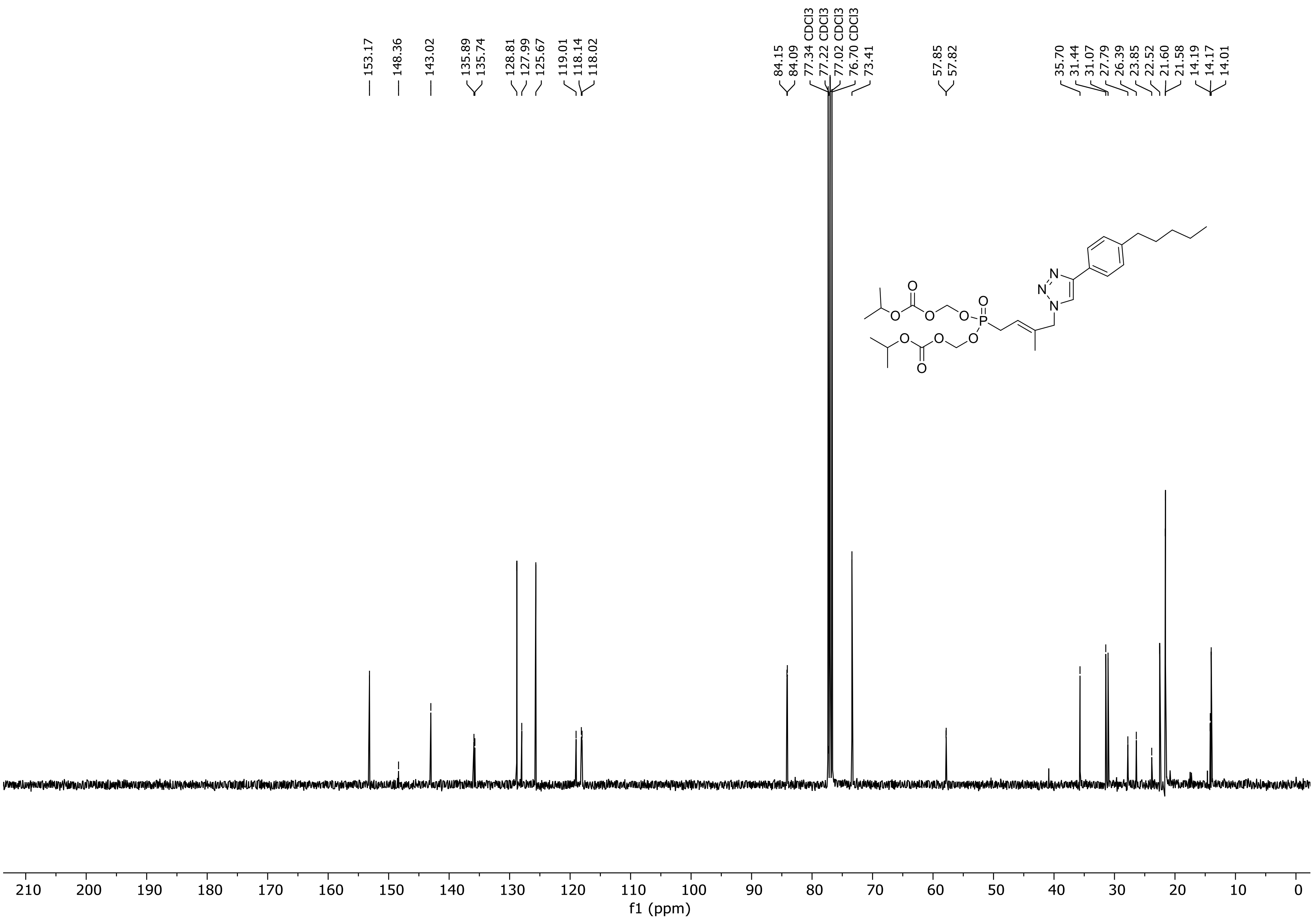

^{31}P NMR (161 MHz, $CDCl_3$) Analysis of Compound 11c

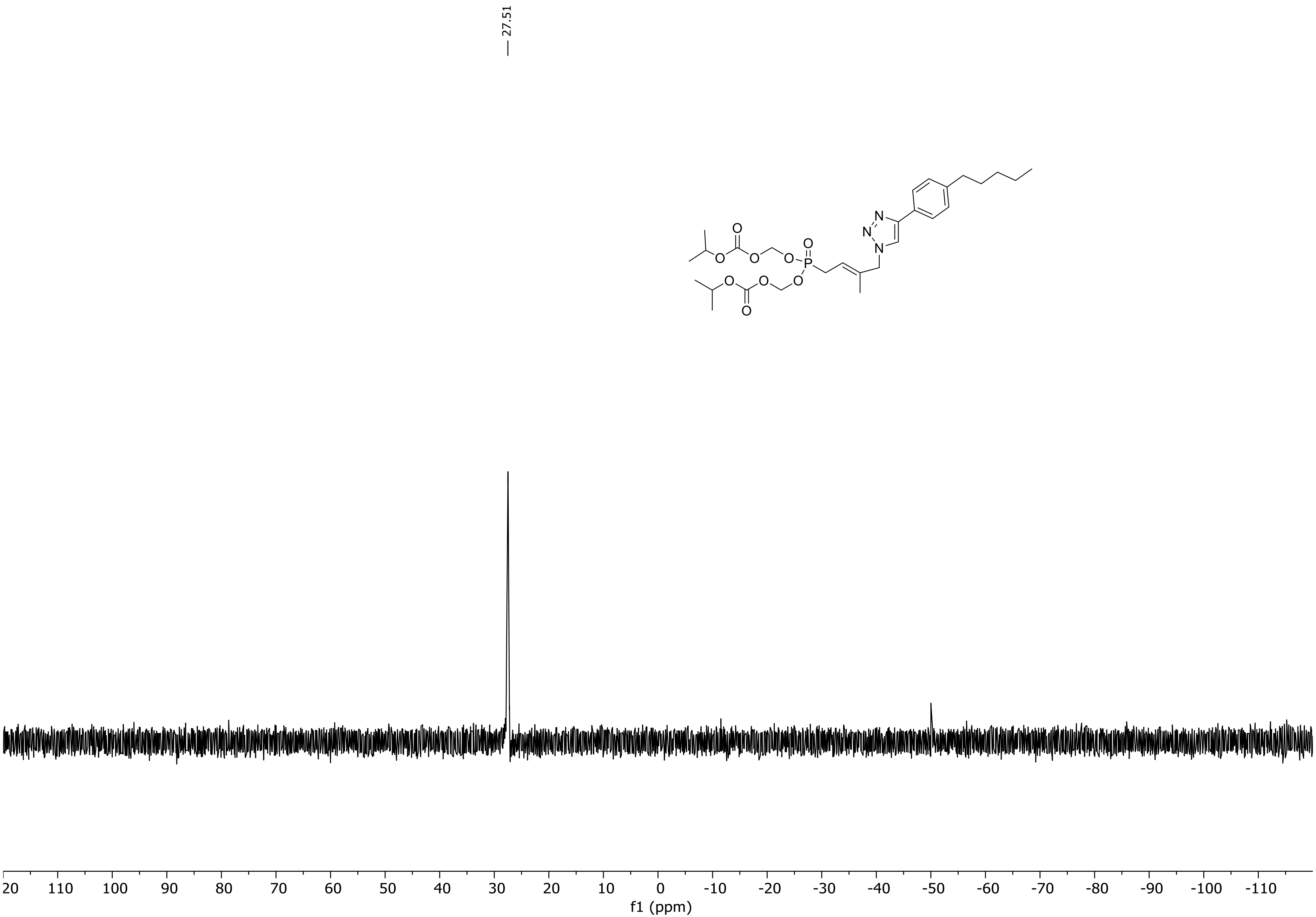

^{1}H NMR (400 MHz, $CDCl_3$) Analysis of Compound 11d

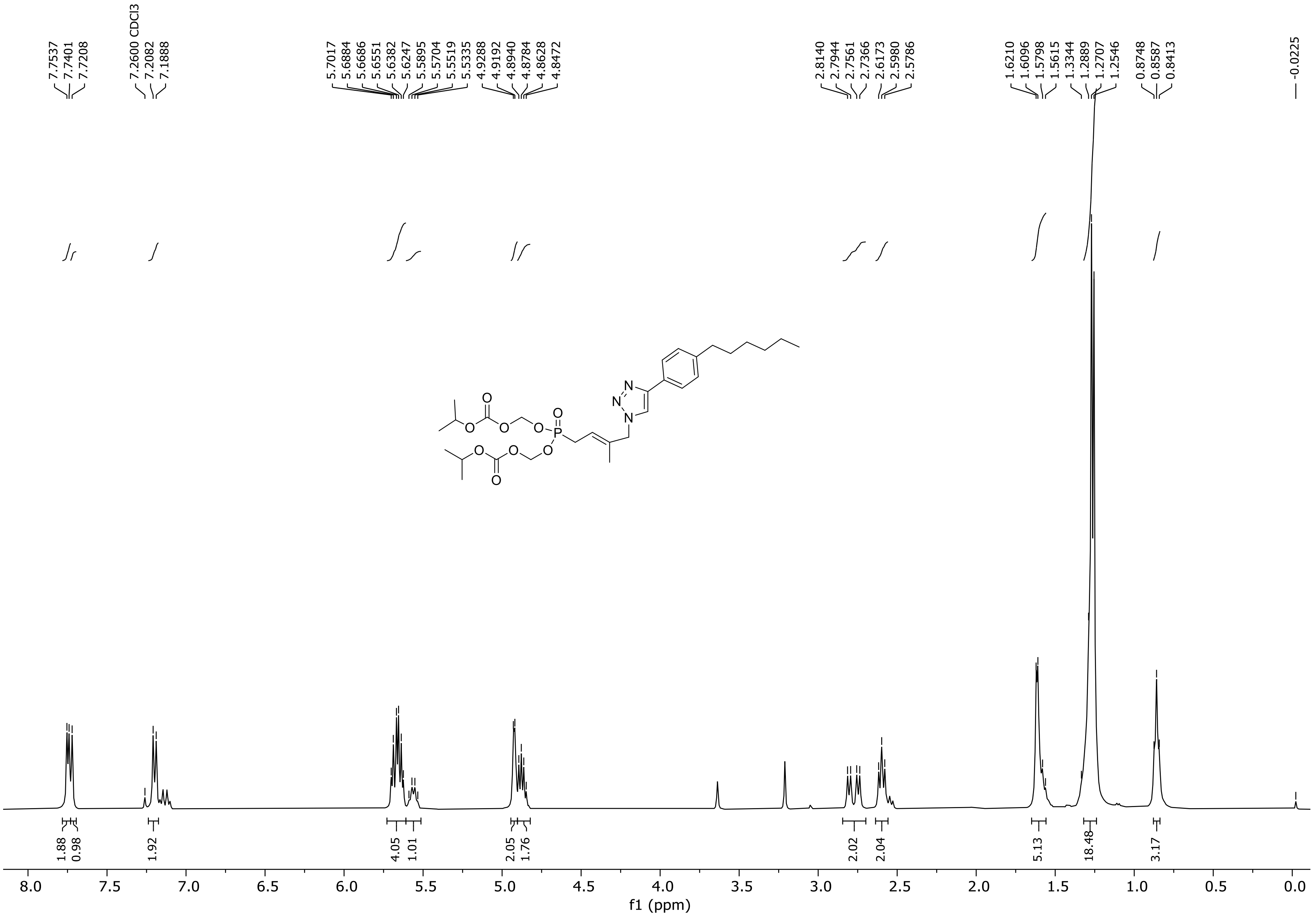

^{13}C NMR (101 MHz, $CDCl_3$) Analysis of Compound 11d

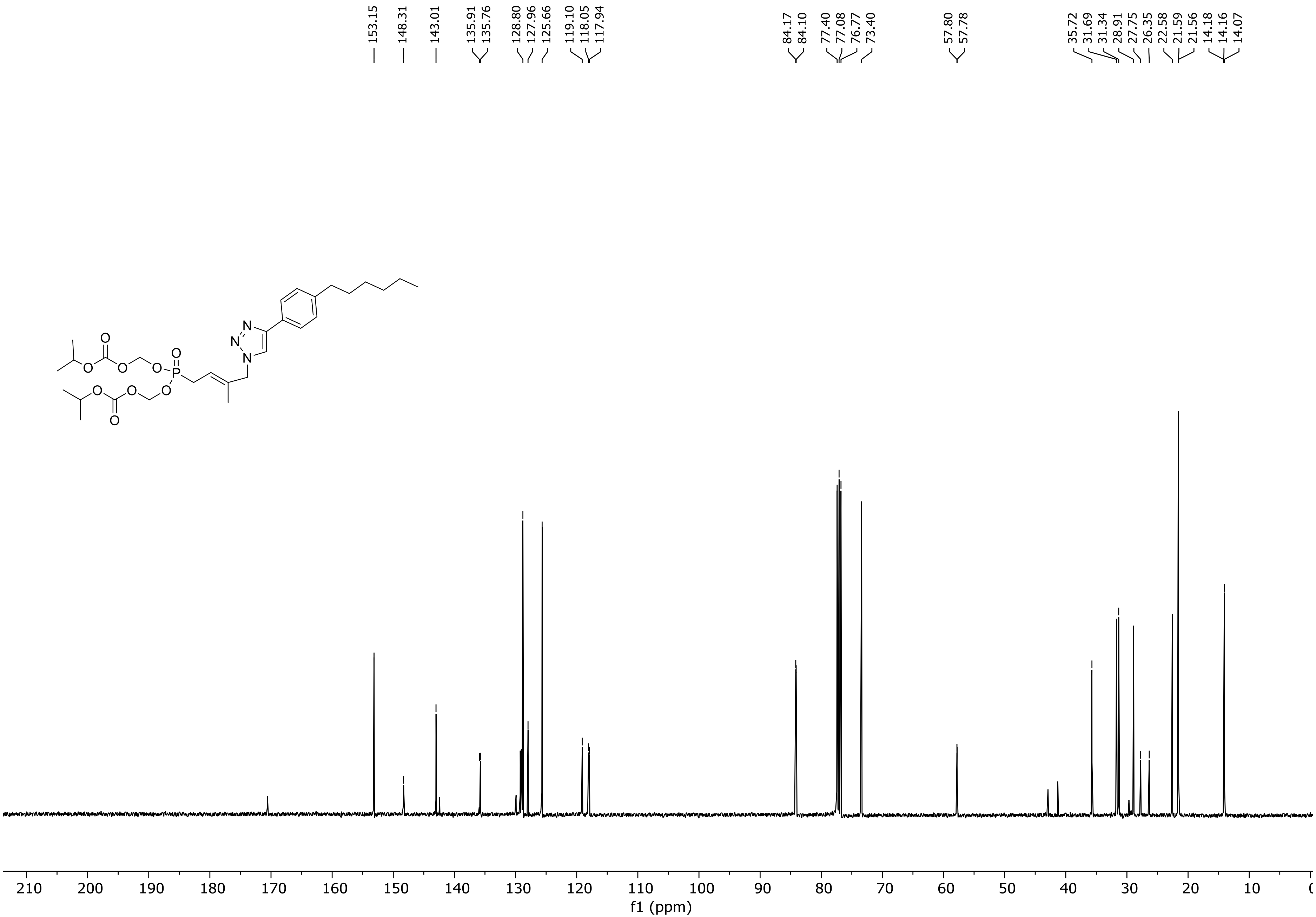

^{31}P NMR (161 MHz, $CDCl_3$) Analysis of Compound 11d

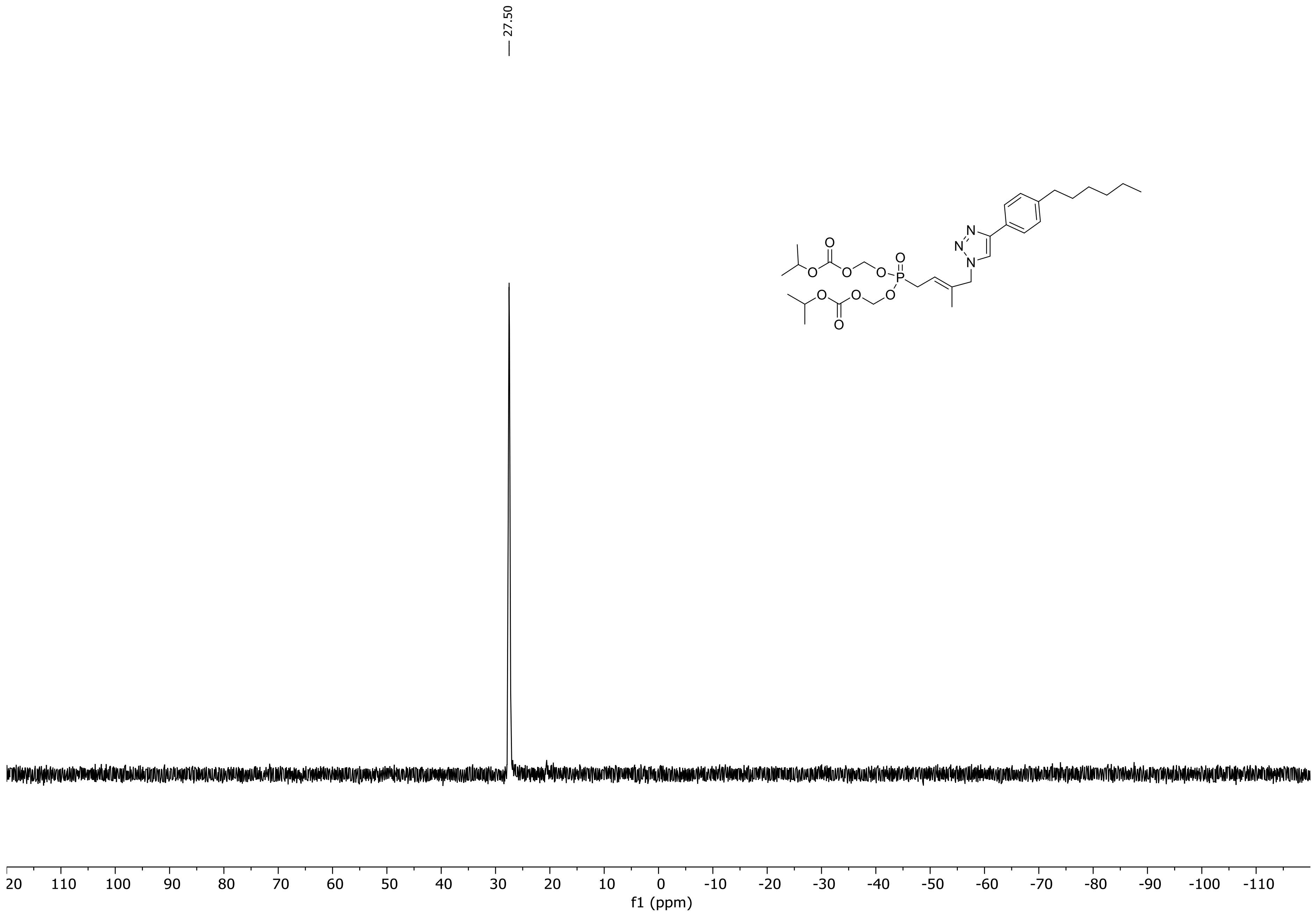

^{1}H NMR (400 MHz, $CDCl_3$) Analysis of Compound 11e

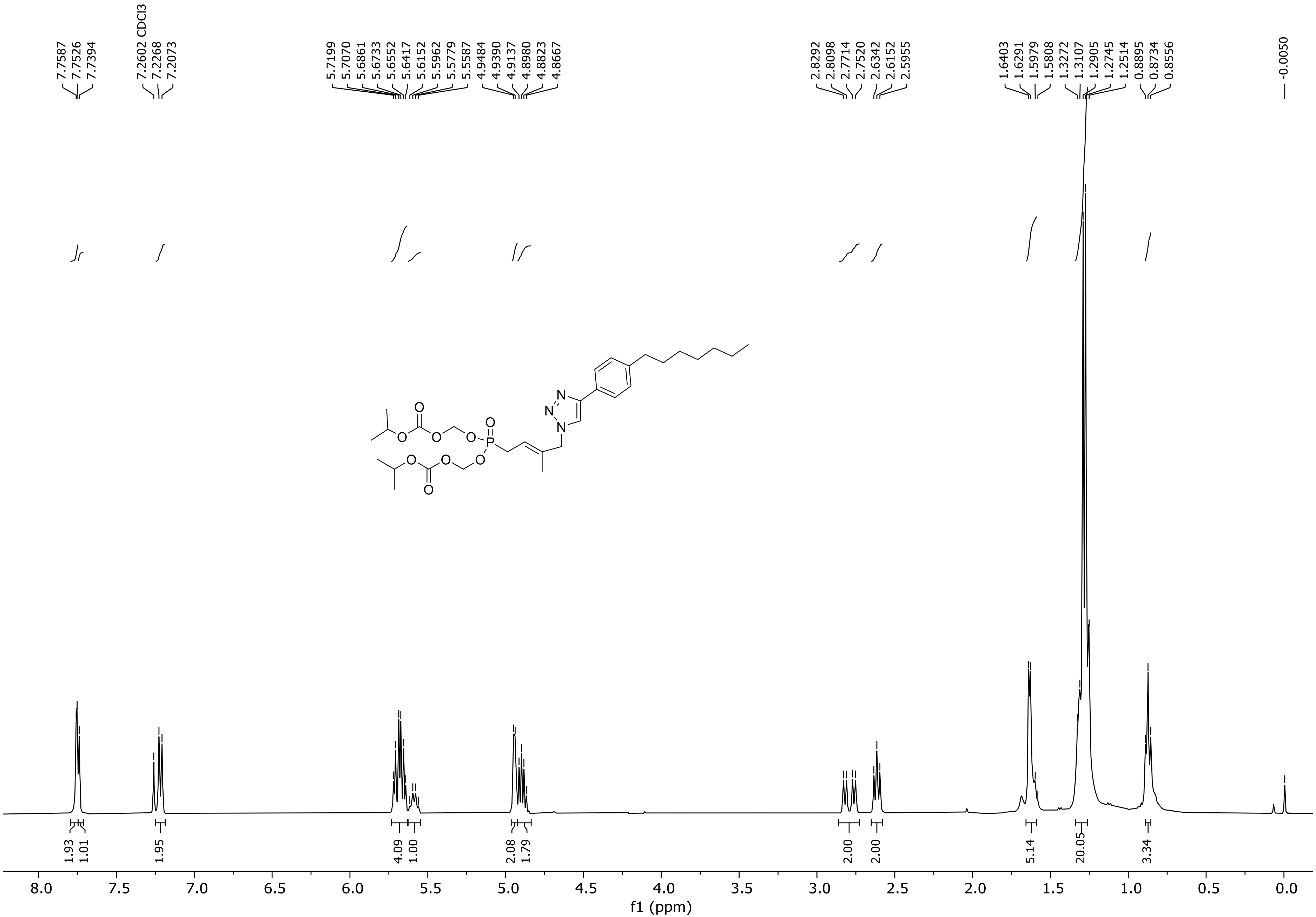

^{13}C NMR (101 MHz, CDCl$_3$) Analysis of Compound 11e

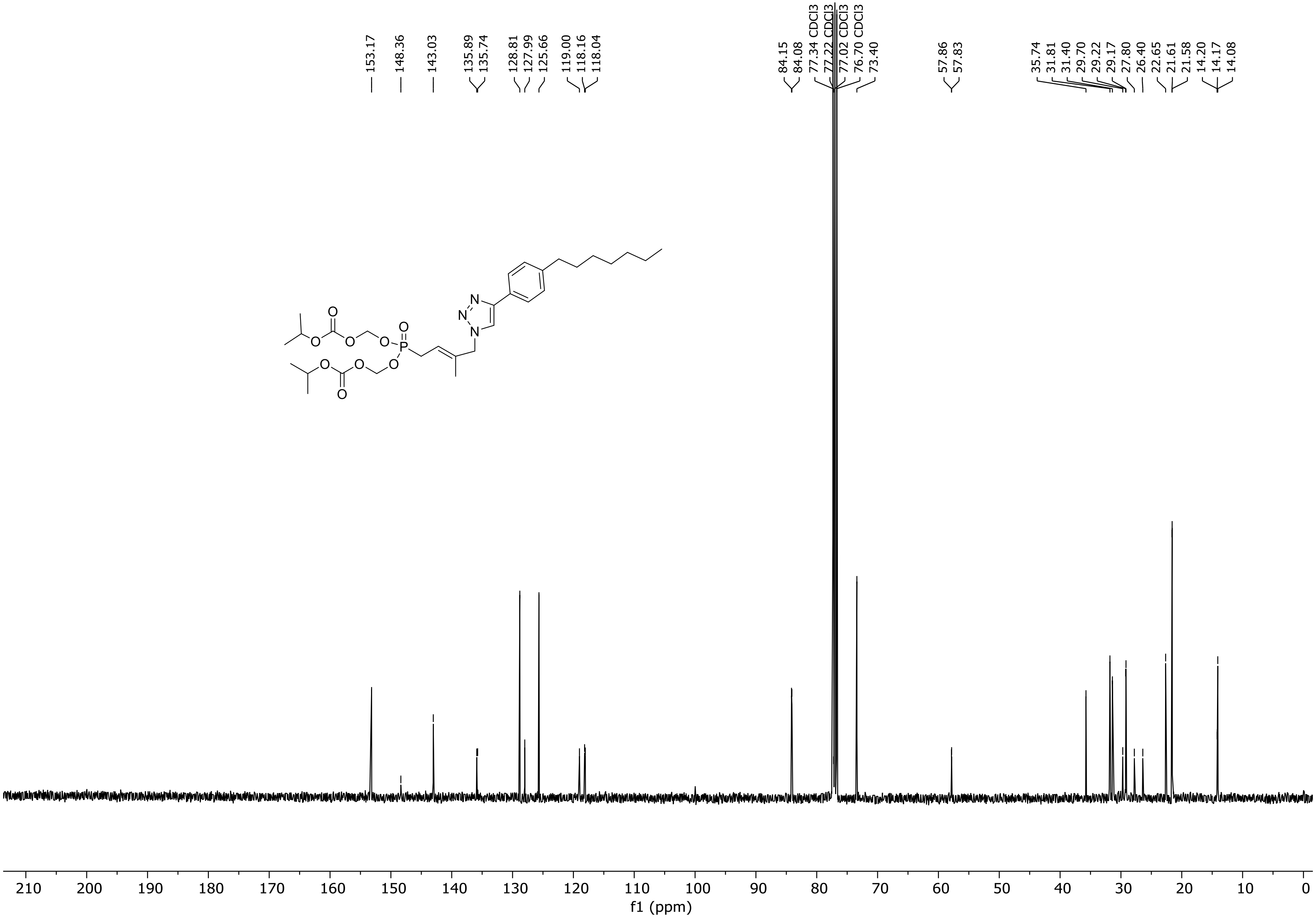

^{31}P NMR (161 MHz, $CDCl_3$) Analysis of Compound 11e

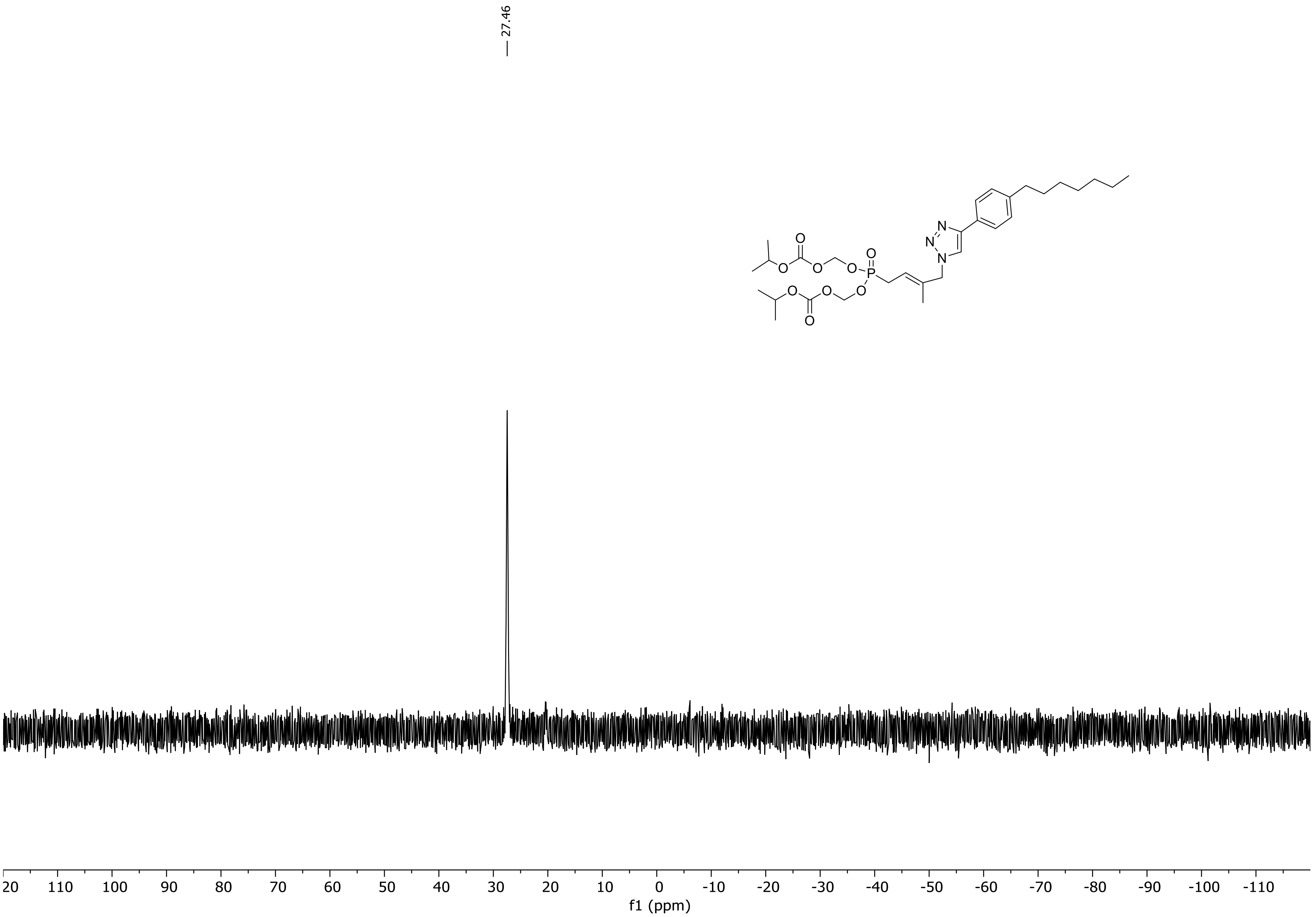

^{1}H NMR (250 MHz, $CDCl_3$) Analysis of Compound 11f

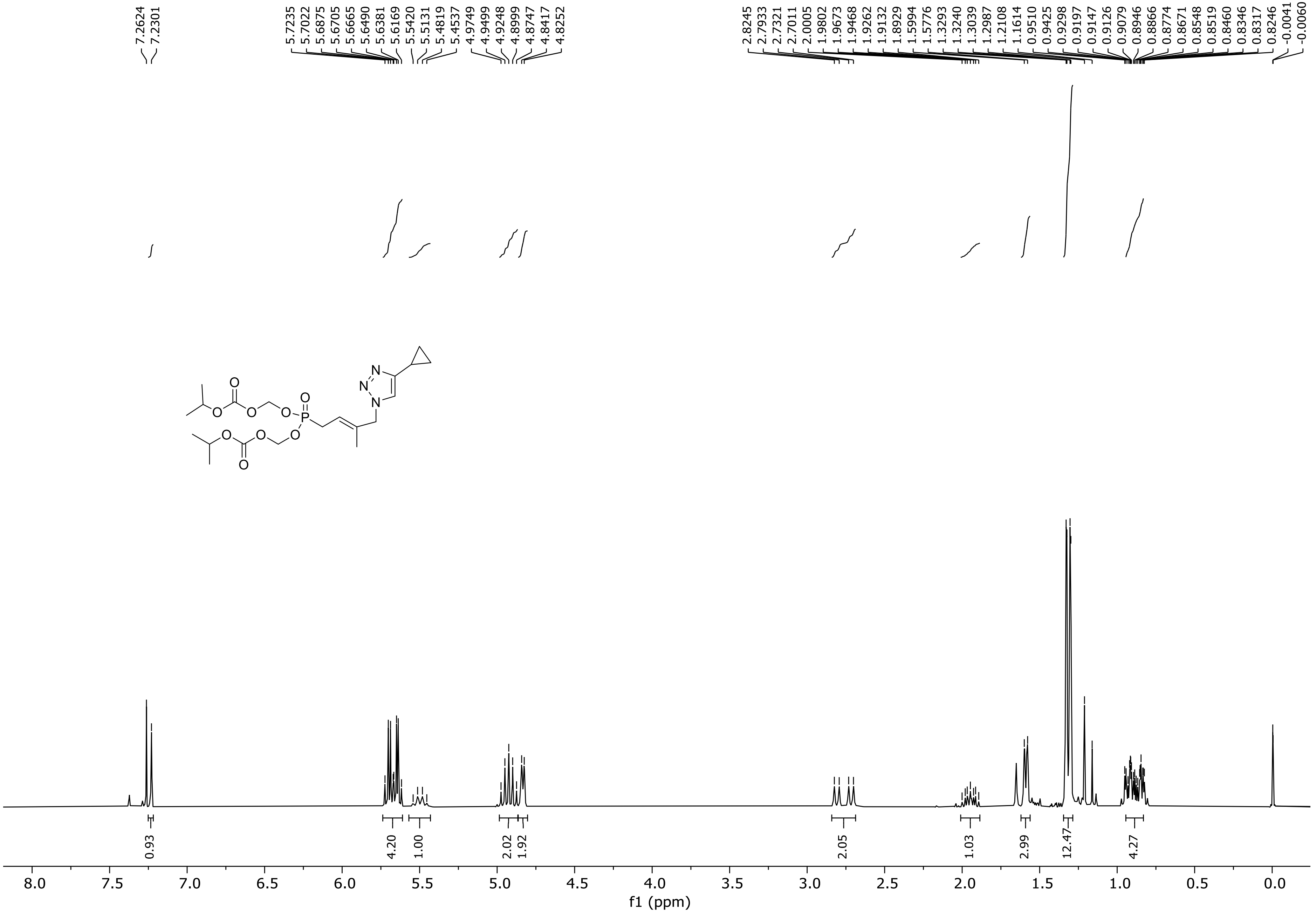

^{13}C NMR (101 MHz, $CDCl_3$) Analysis of Compound 11f

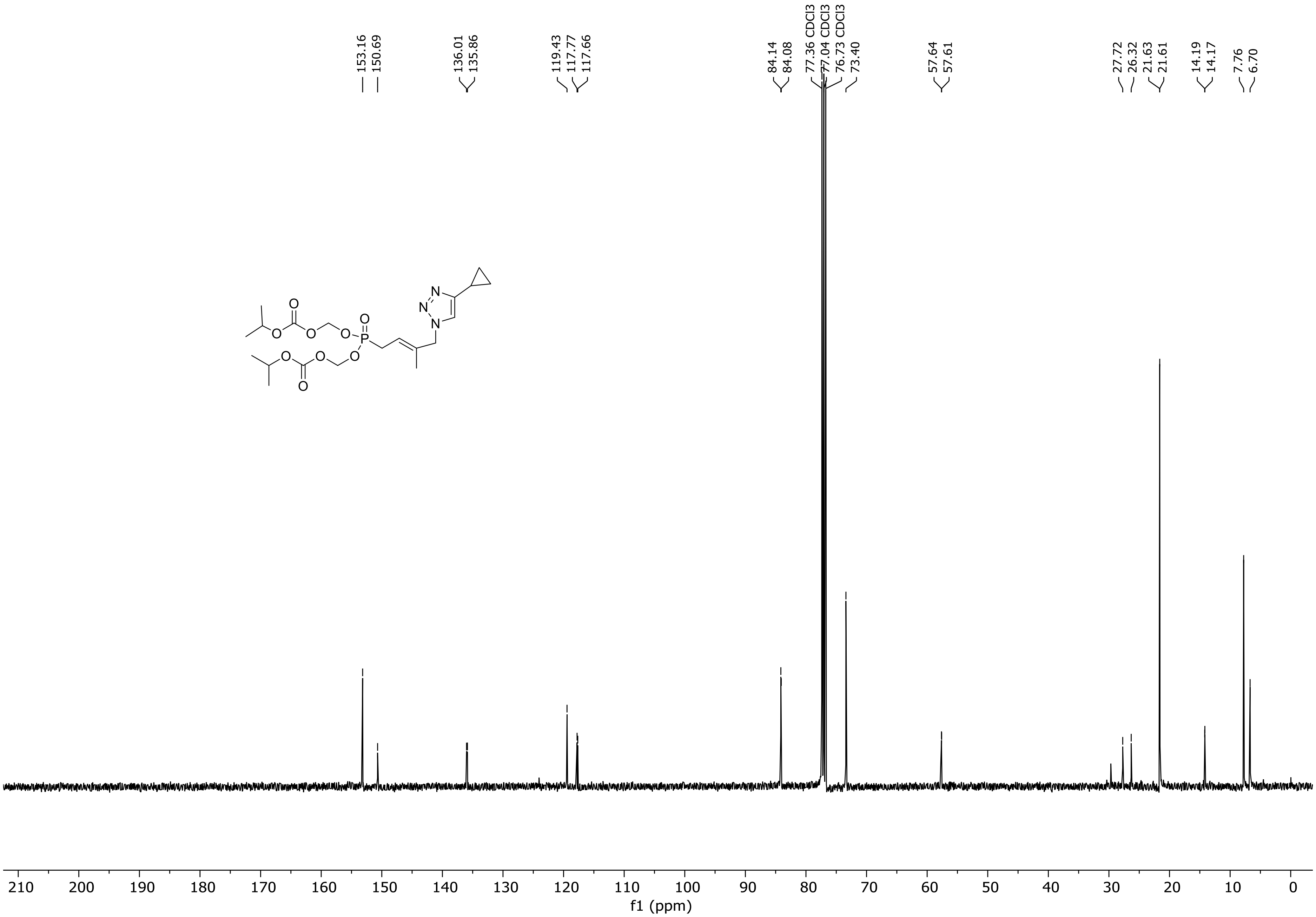

^{31}P NMR (161 MHz, $CDCl_3$) Analysis of Compound 11f

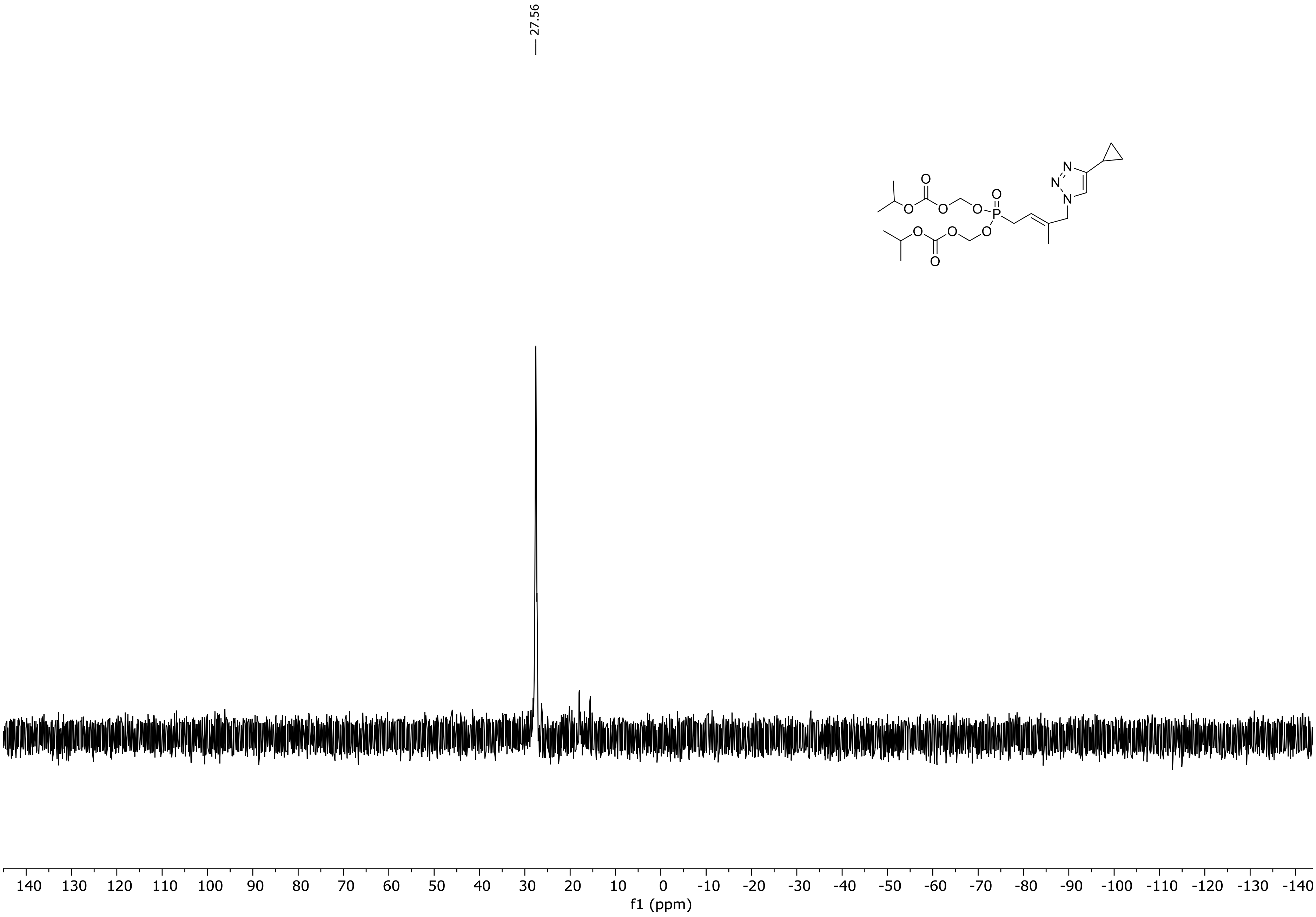

^{1}H NMR (400 MHz, $CDCl_3$) Analysis of Compound 11g

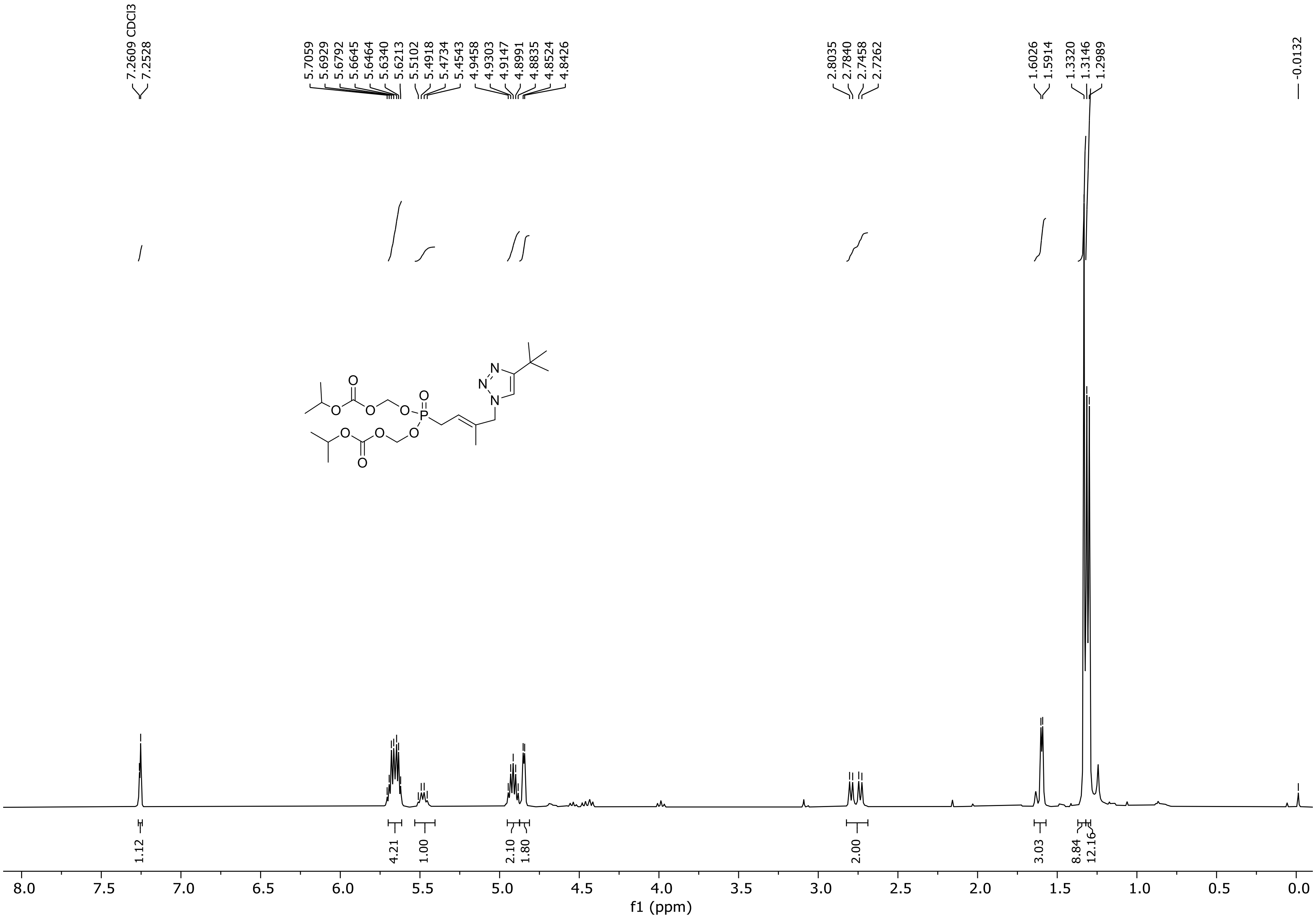

^{13}C NMR (101 MHz, $CDCl_3$) Analysis of Compound 11g

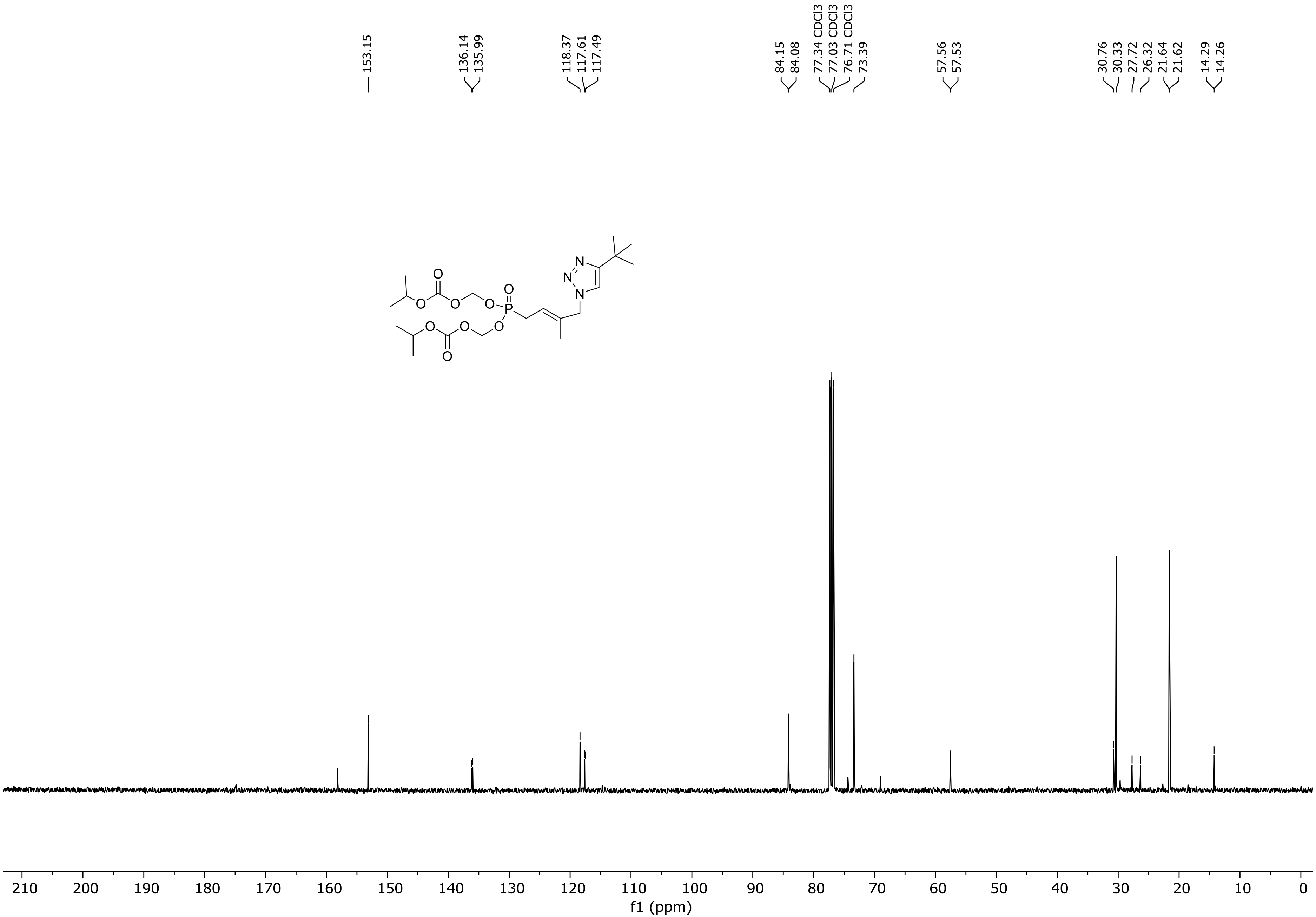

^{31}P NMR (161 MHz, $CDCl_3$) Analysis of Compound 11g

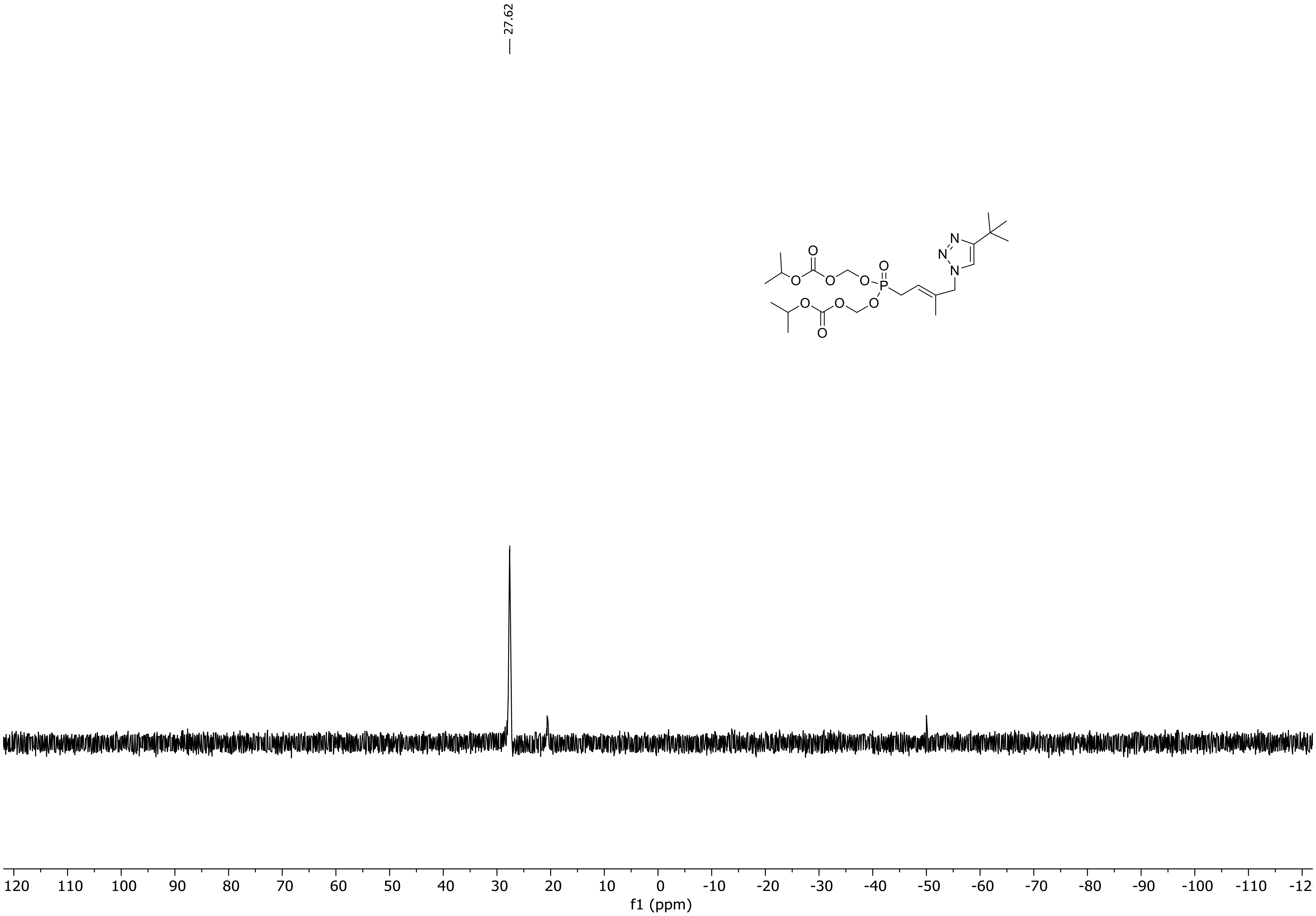

^{1}H NMR (400 MHz, $CDCl_3$) Analysis of Compound 11h

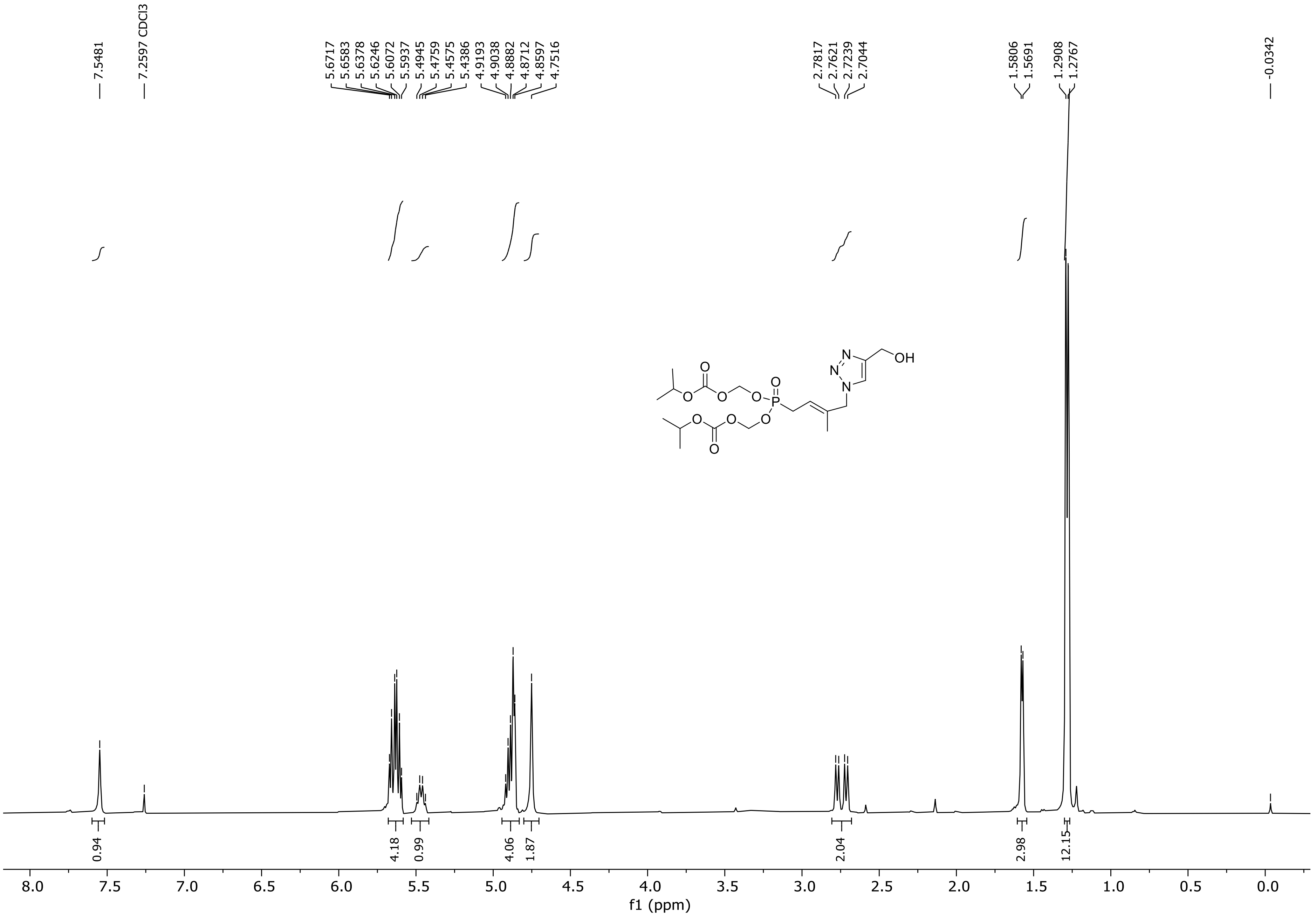

^{13}C NMR (101 MHz, CDCl3) Analysis of Compound 11h

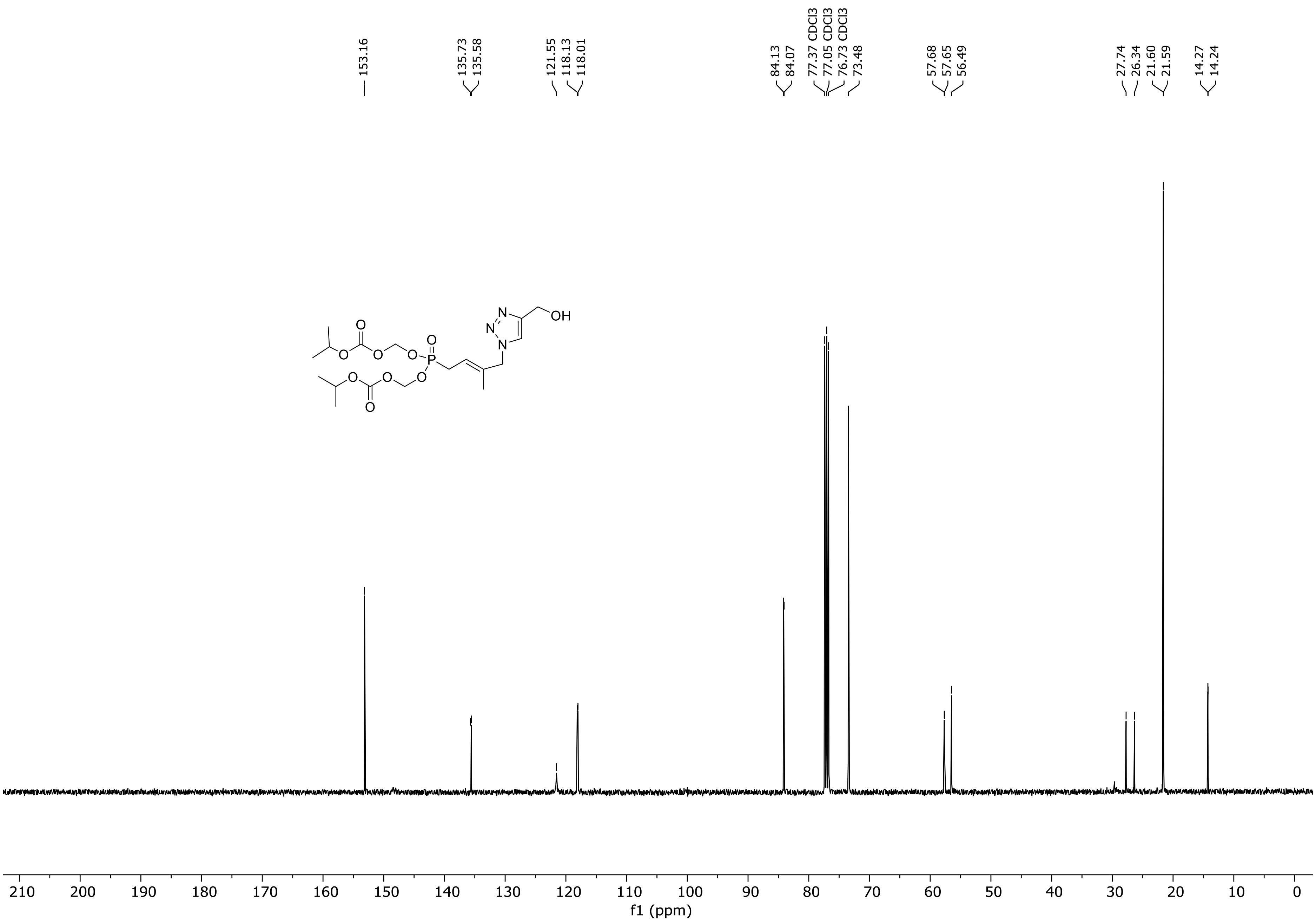

^{31}P NMR (161 MHz, $CDCl_3$) Analysis of Compound 11h

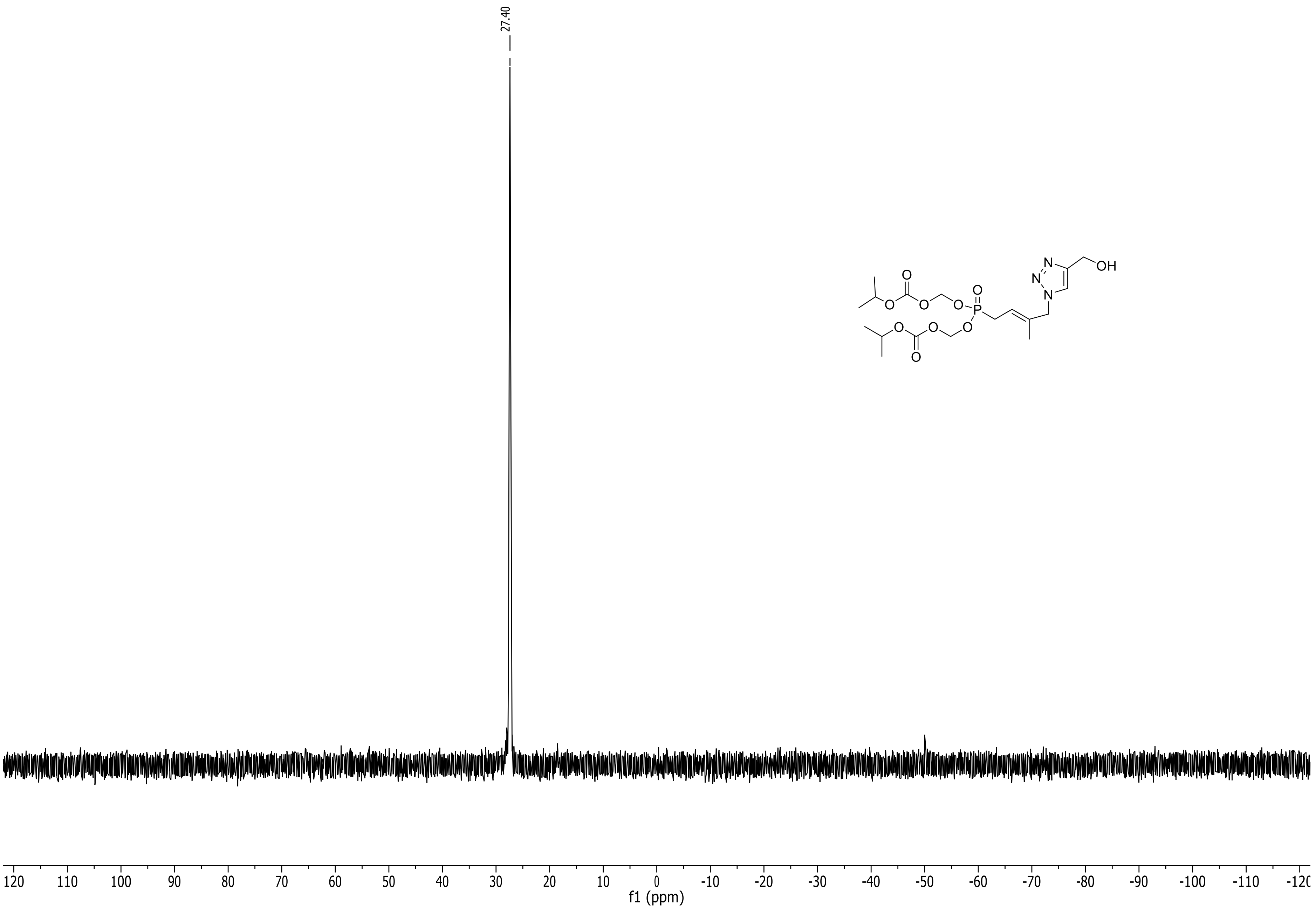

^{1}H NMR (400 MHz, $CDCl_3$) Analysis of Compound 11i

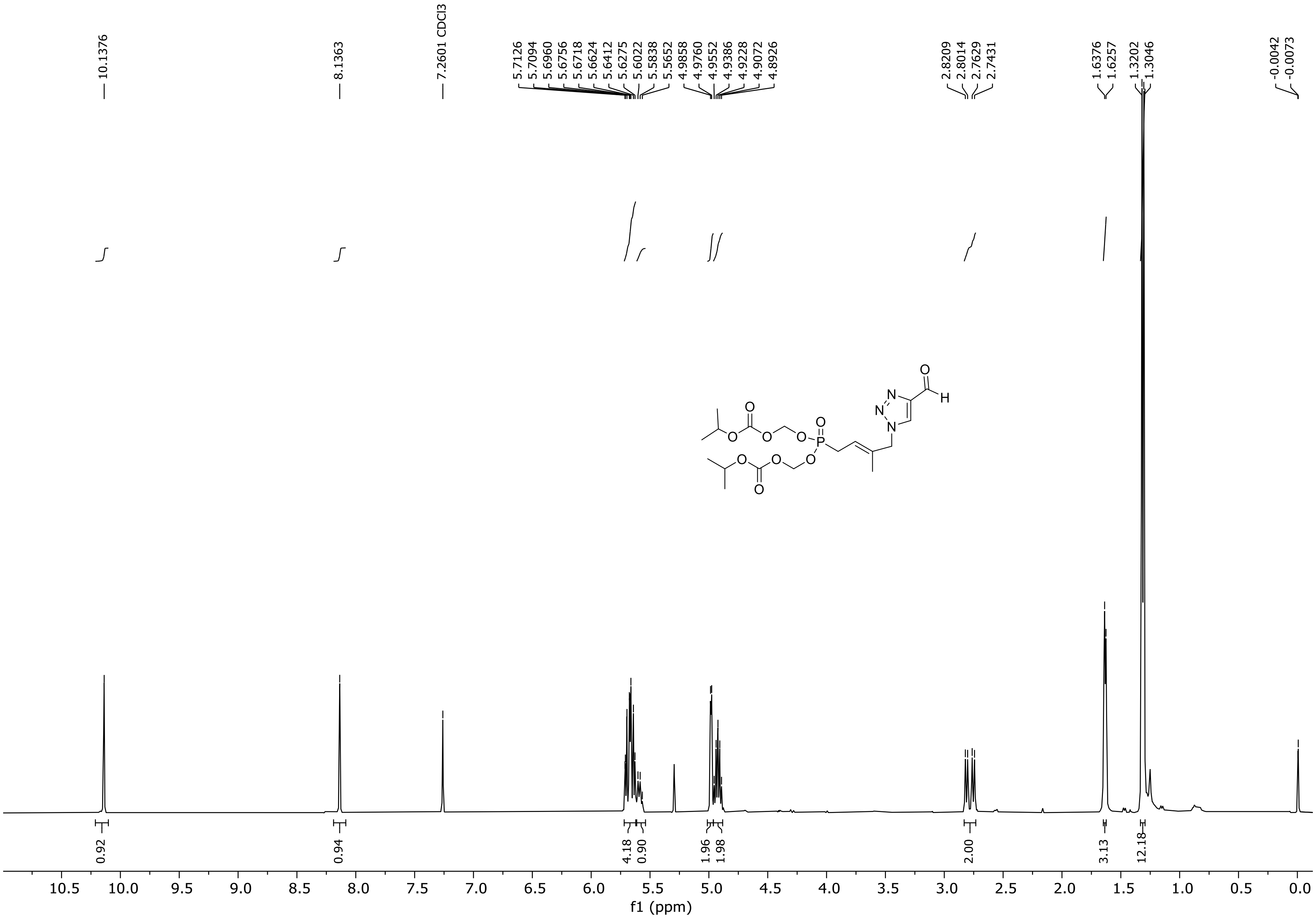

^{13}C NMR (101 MHz, CDCl$_3$) Analysis of Compound 11i

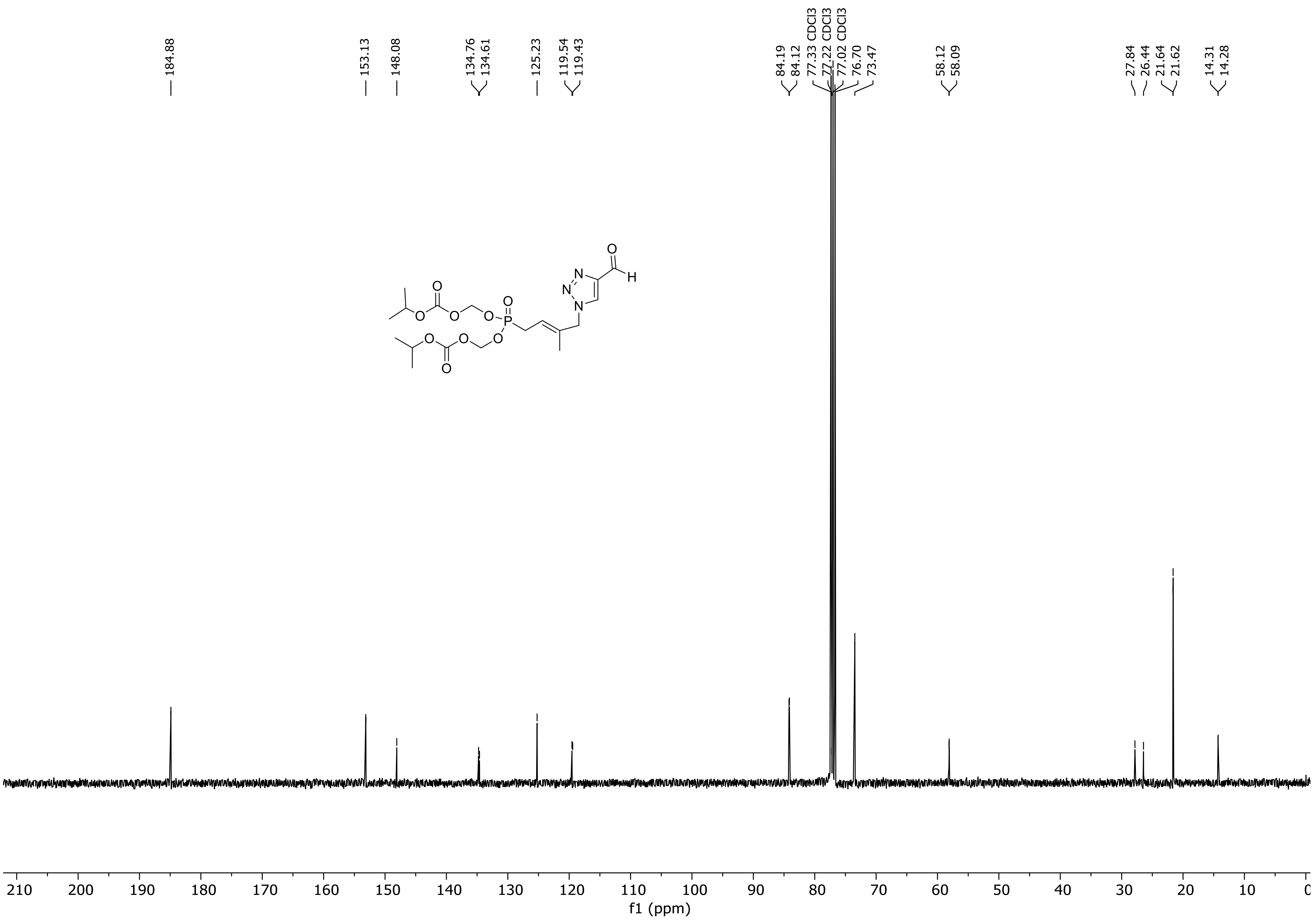

^{31}P NMR (161 MHz, CDCl$_3$) Analysis of Compound 11i

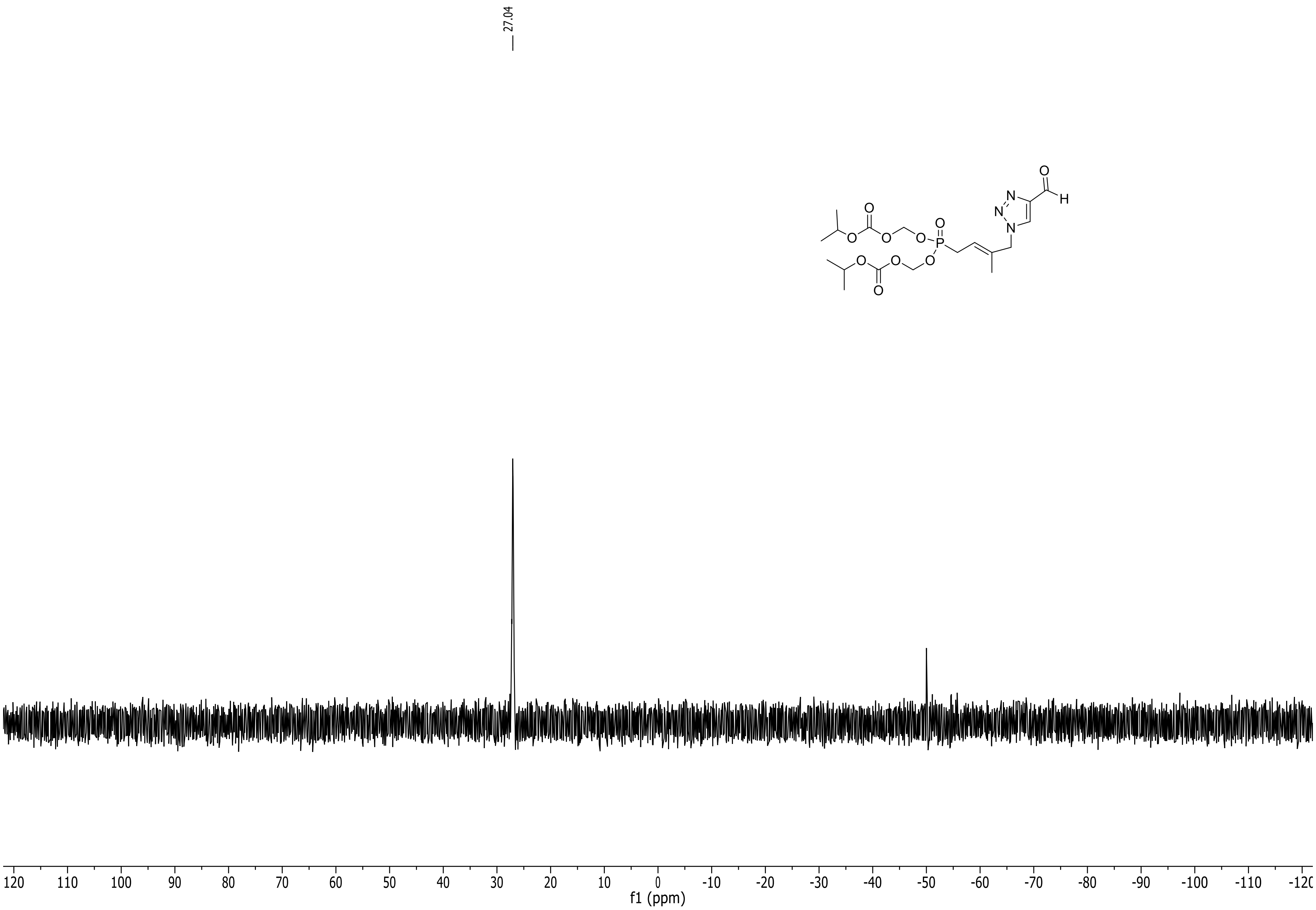

^{1}H NMR (400 MHz, Acetone-d_6) Analysis of Compound 11j

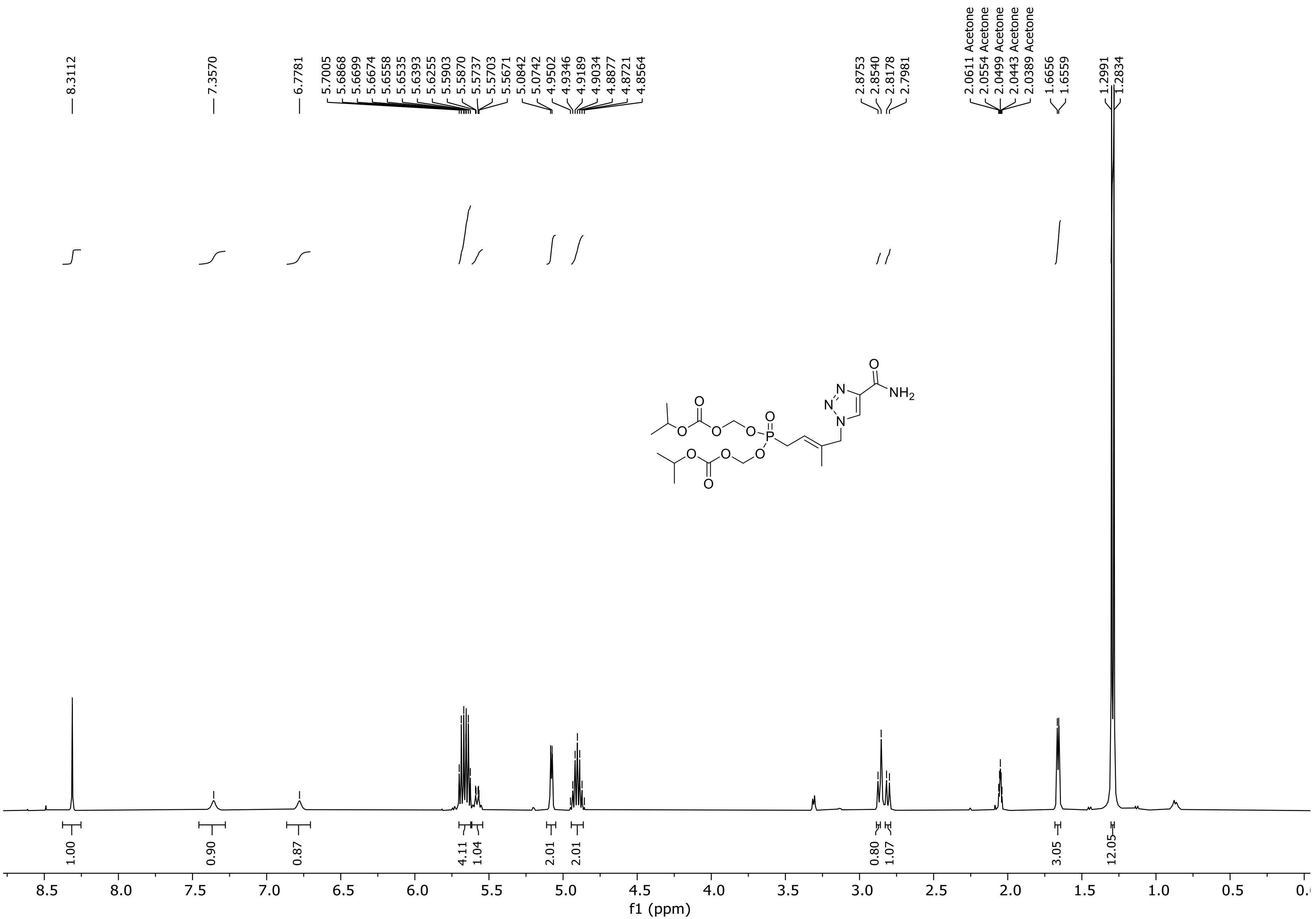

^{13}C NMR (101 MHz, Acetone-d$_6$) Analysis of Compound 11j

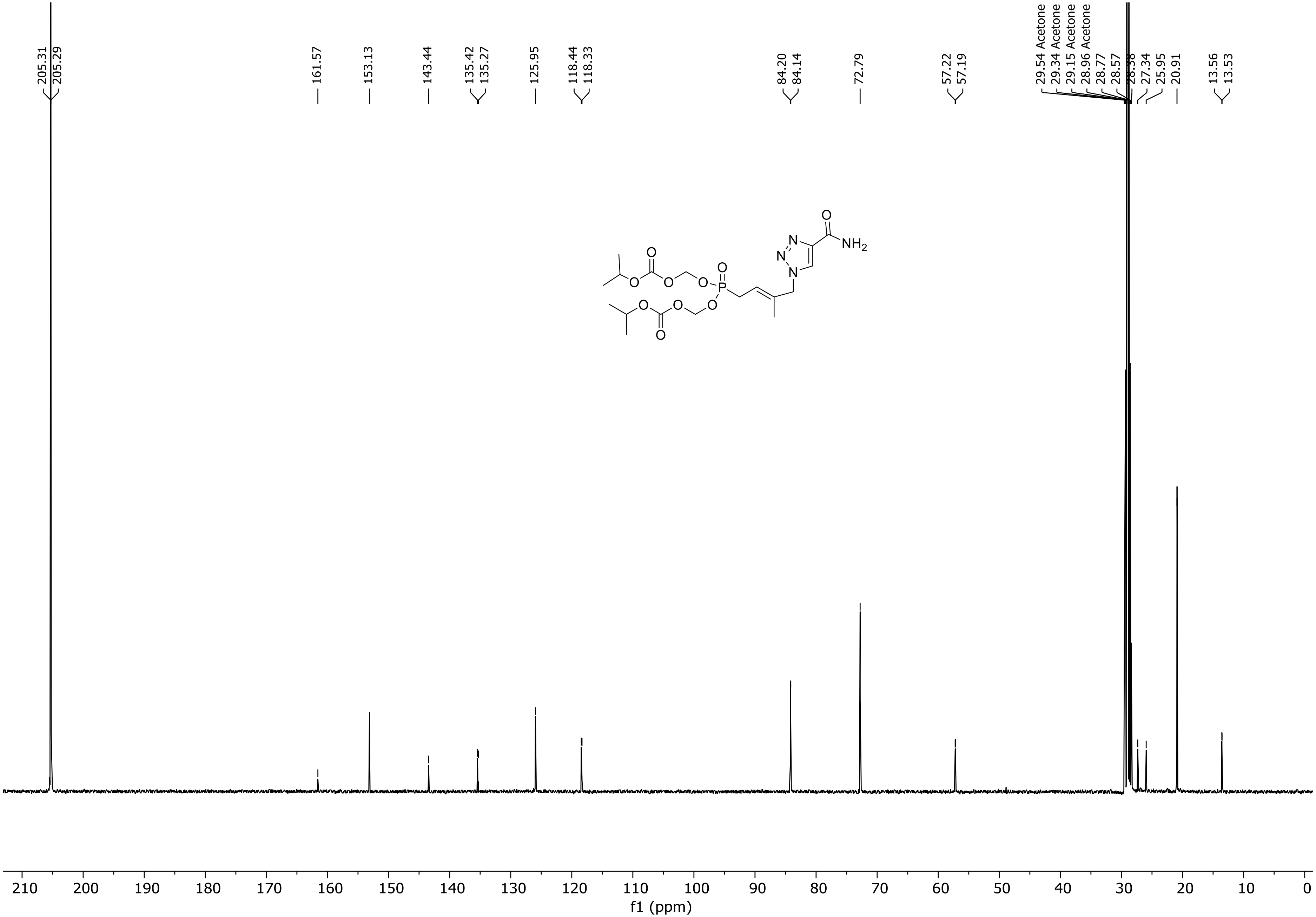

^{31}P NMR (161 MHz, Acetone-d$_6$) Analysis of Compound 11j

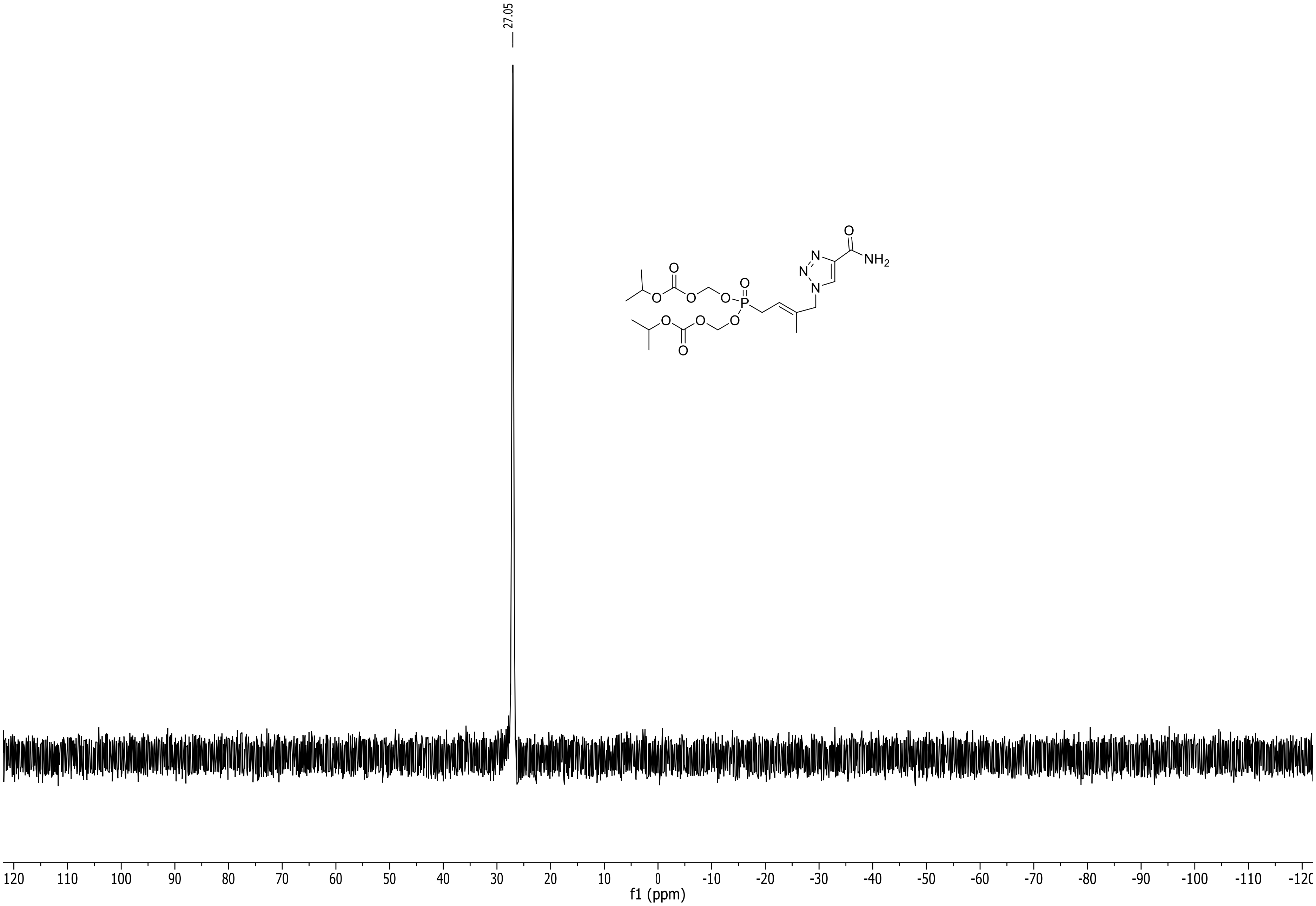

^{1}H NMR (400 MHz, $CDCl_3$) Analysis of Compound 11k

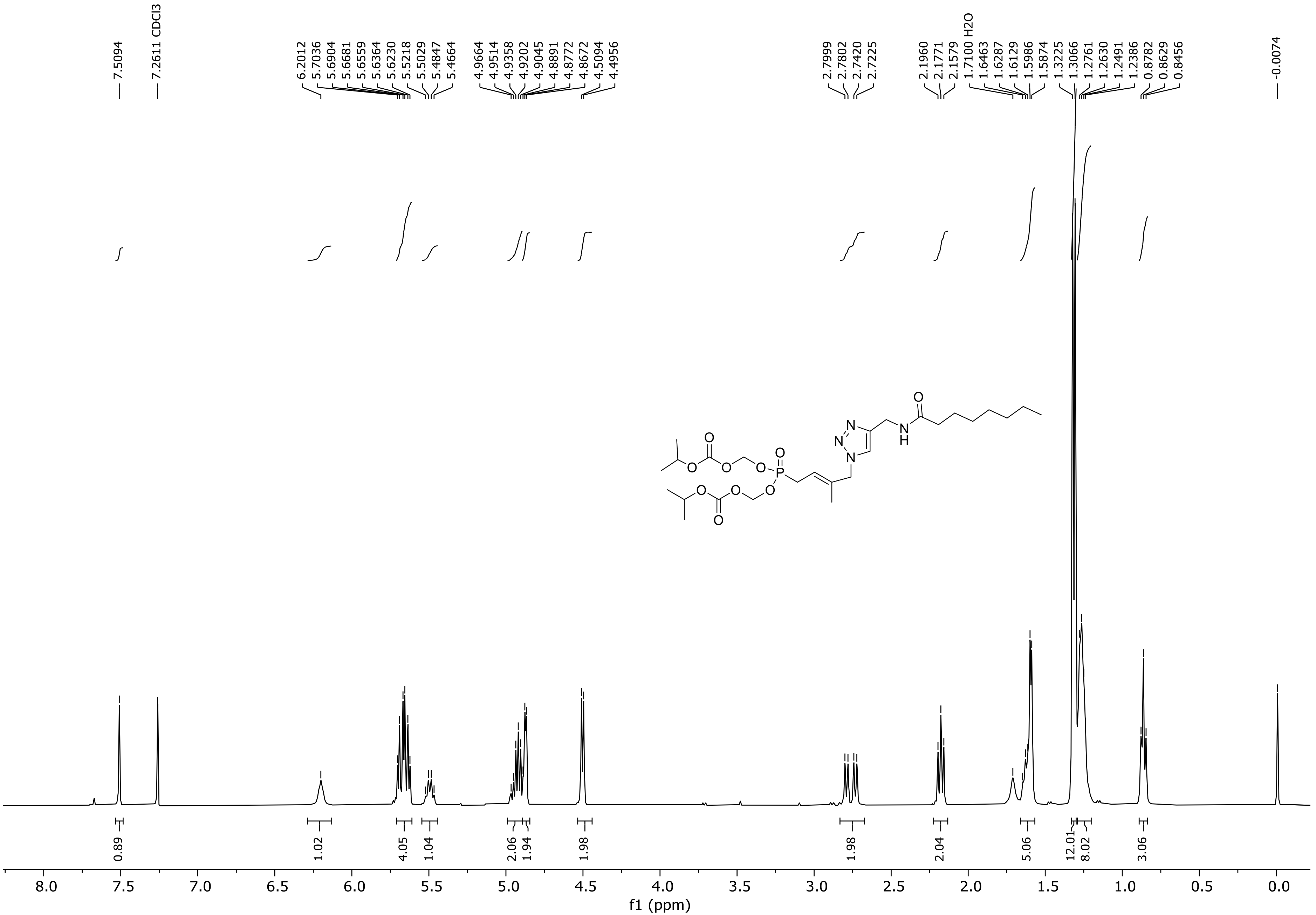

^{13}C NMR (101 MHz, $CDCl_3$) Analysis of Compound 11k

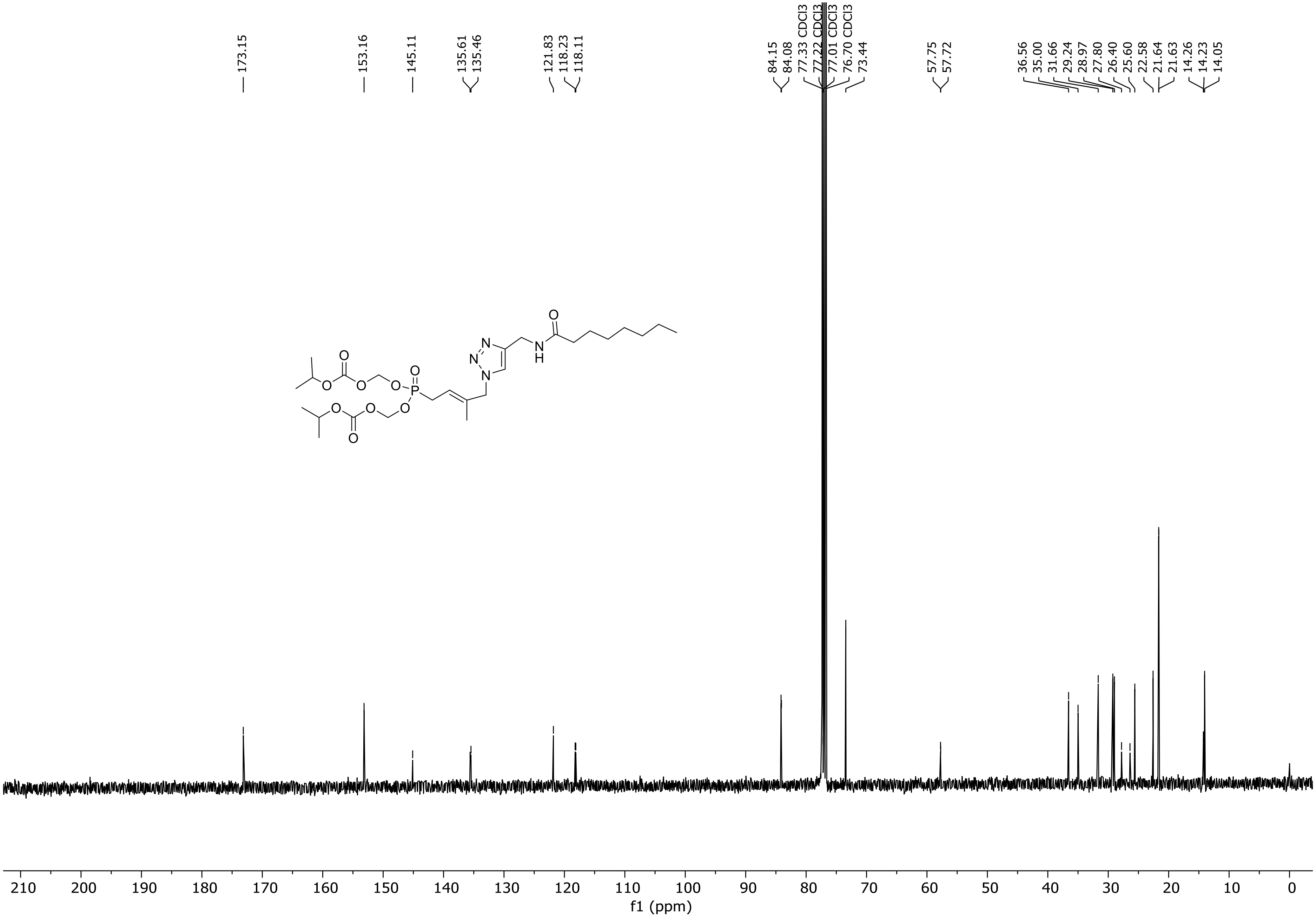

^{31}P NMR (161 MHz, $CDCl_3$) Analysis of Compound 11k

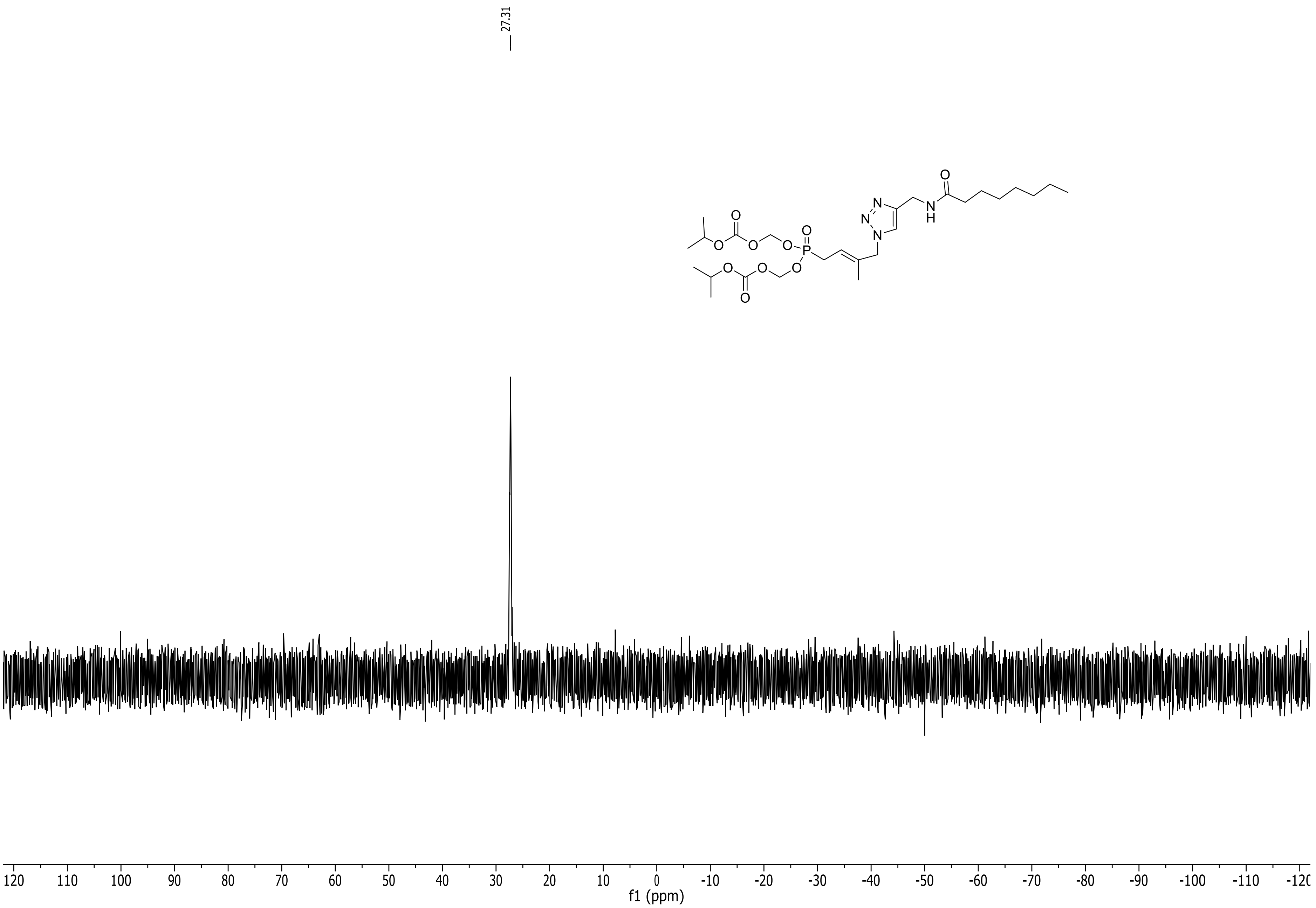

^{1}H NMR (400 MHz, CDCl3) Analysis of Compound 11l

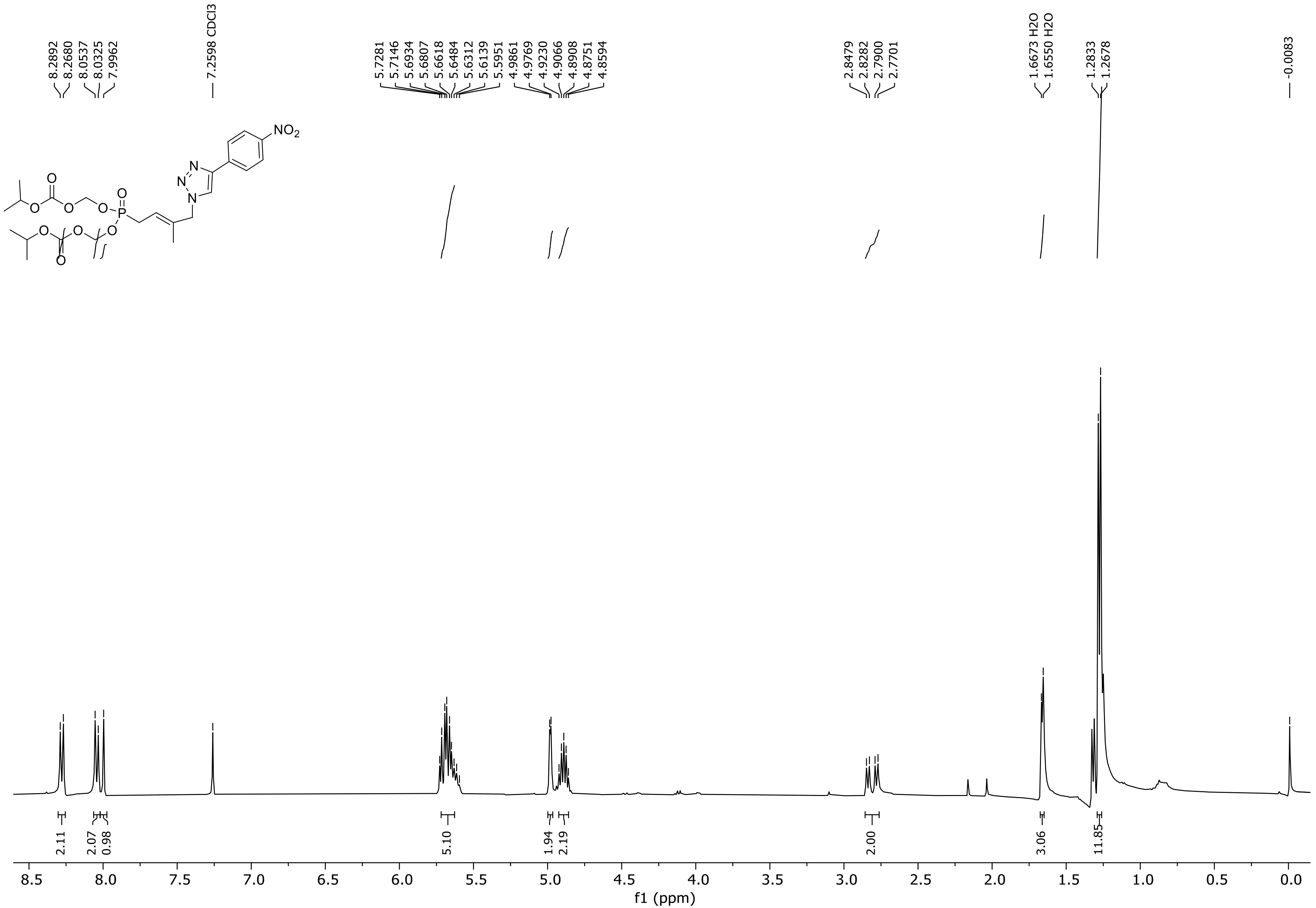

^{13}C NMR (101 MHz, $CDCl_3$) Analysis of Compound 11l

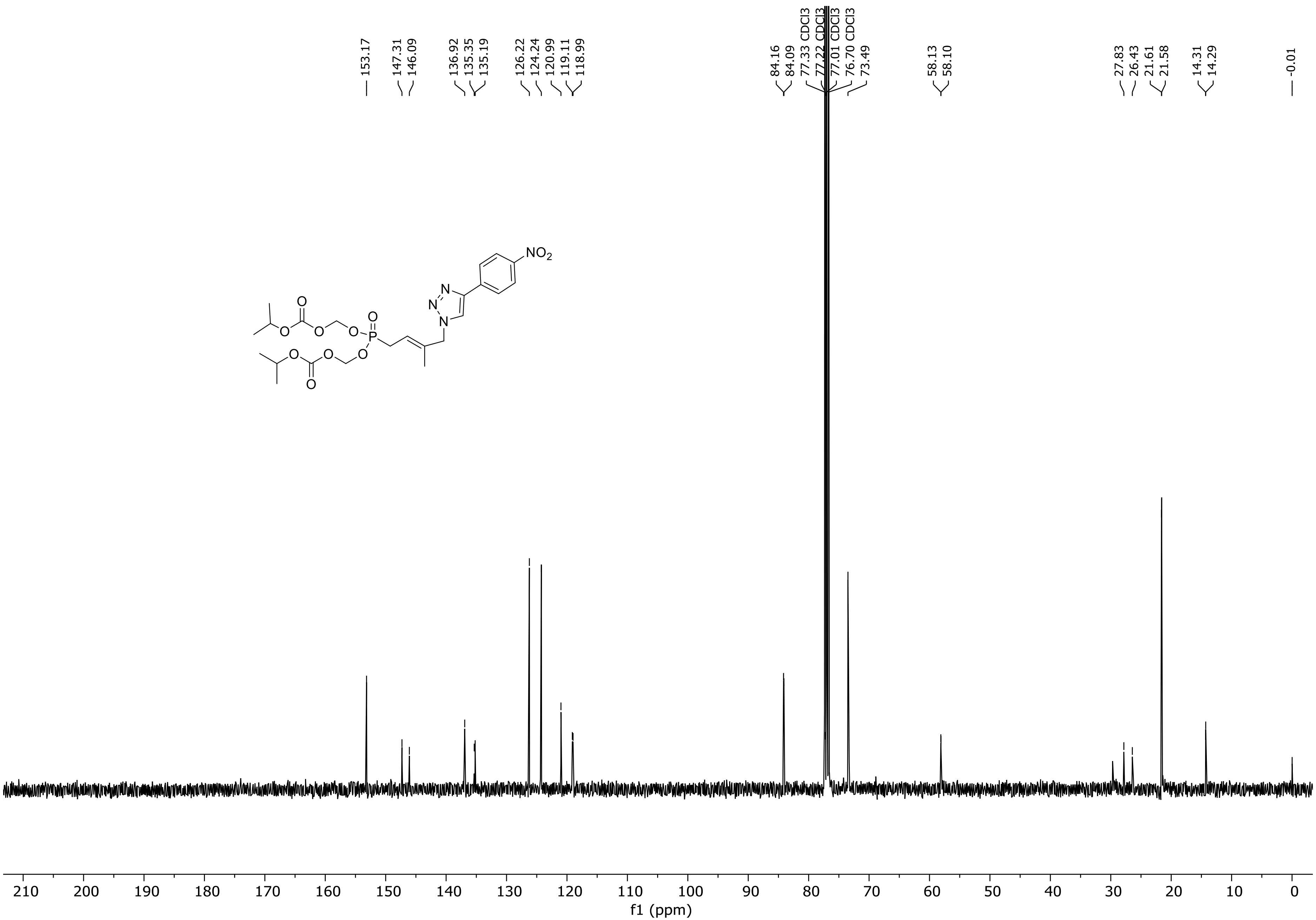

^{31}P NMR (161 MHz, CDCl$_3$) Analysis of Compound 11l

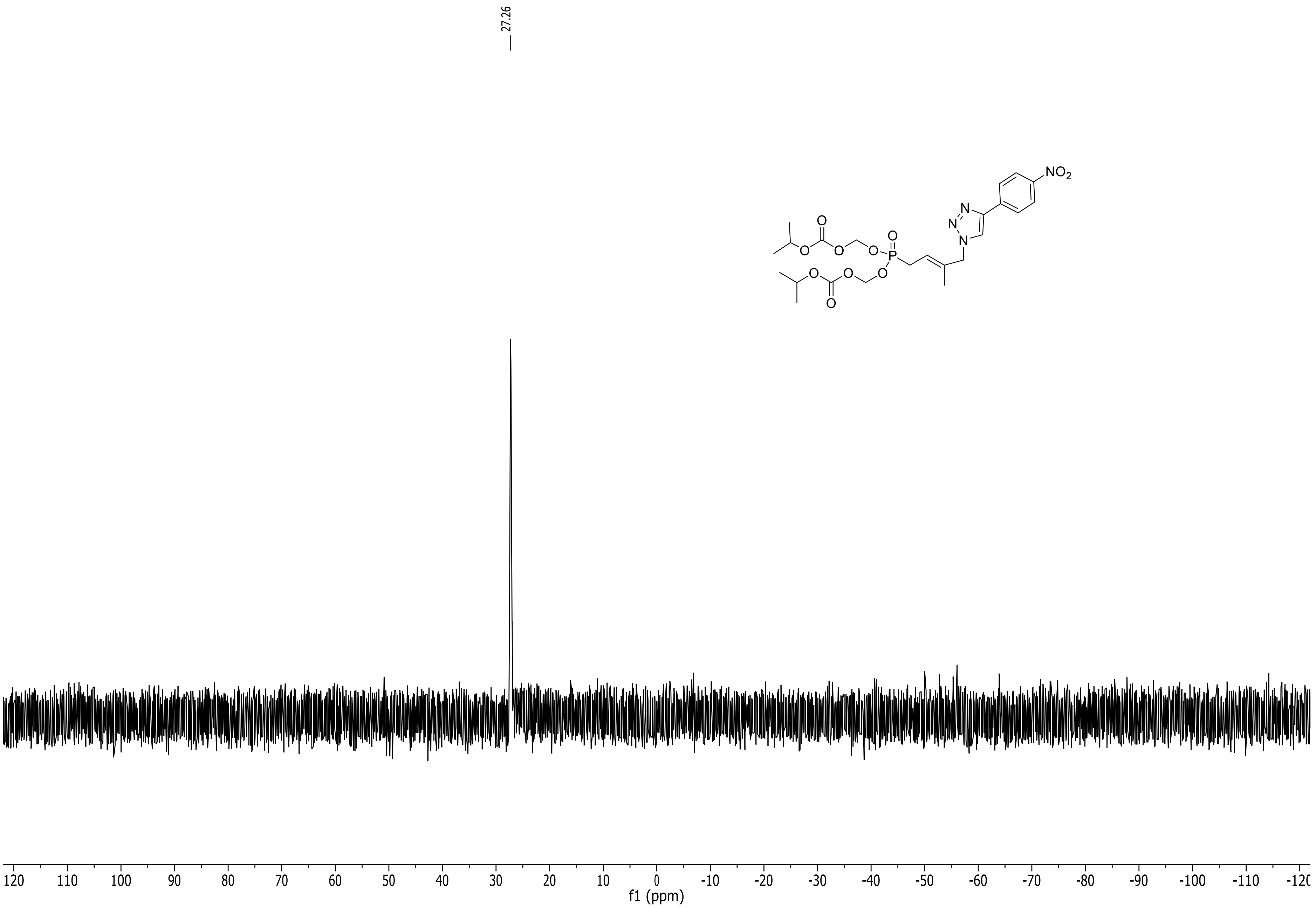

^{1}H NMR (400 MHz, MeOD) Analysis of Compound 11m

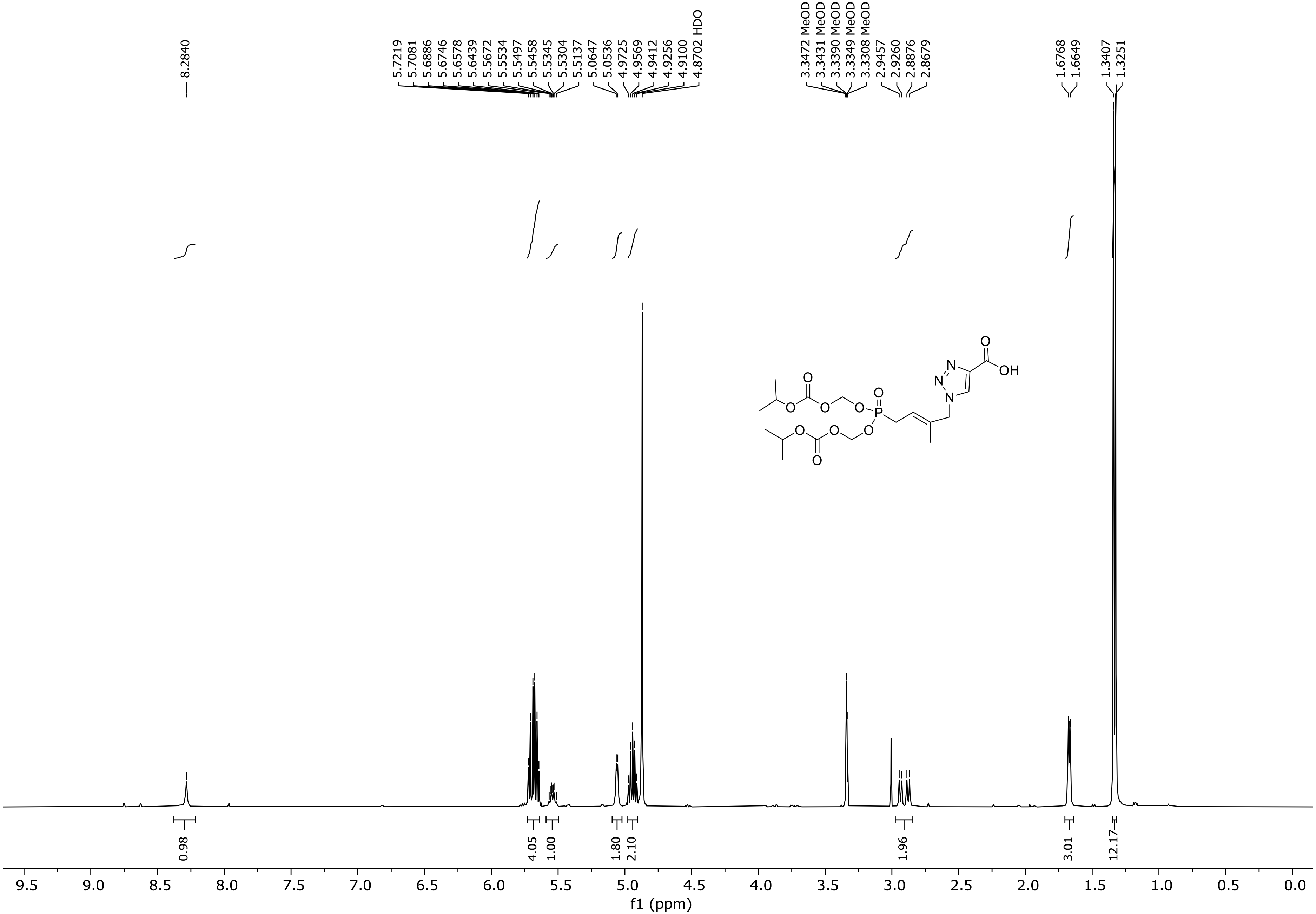

^{13}C NMR (101 MHz, MeOD) Analysis of Compound 11m

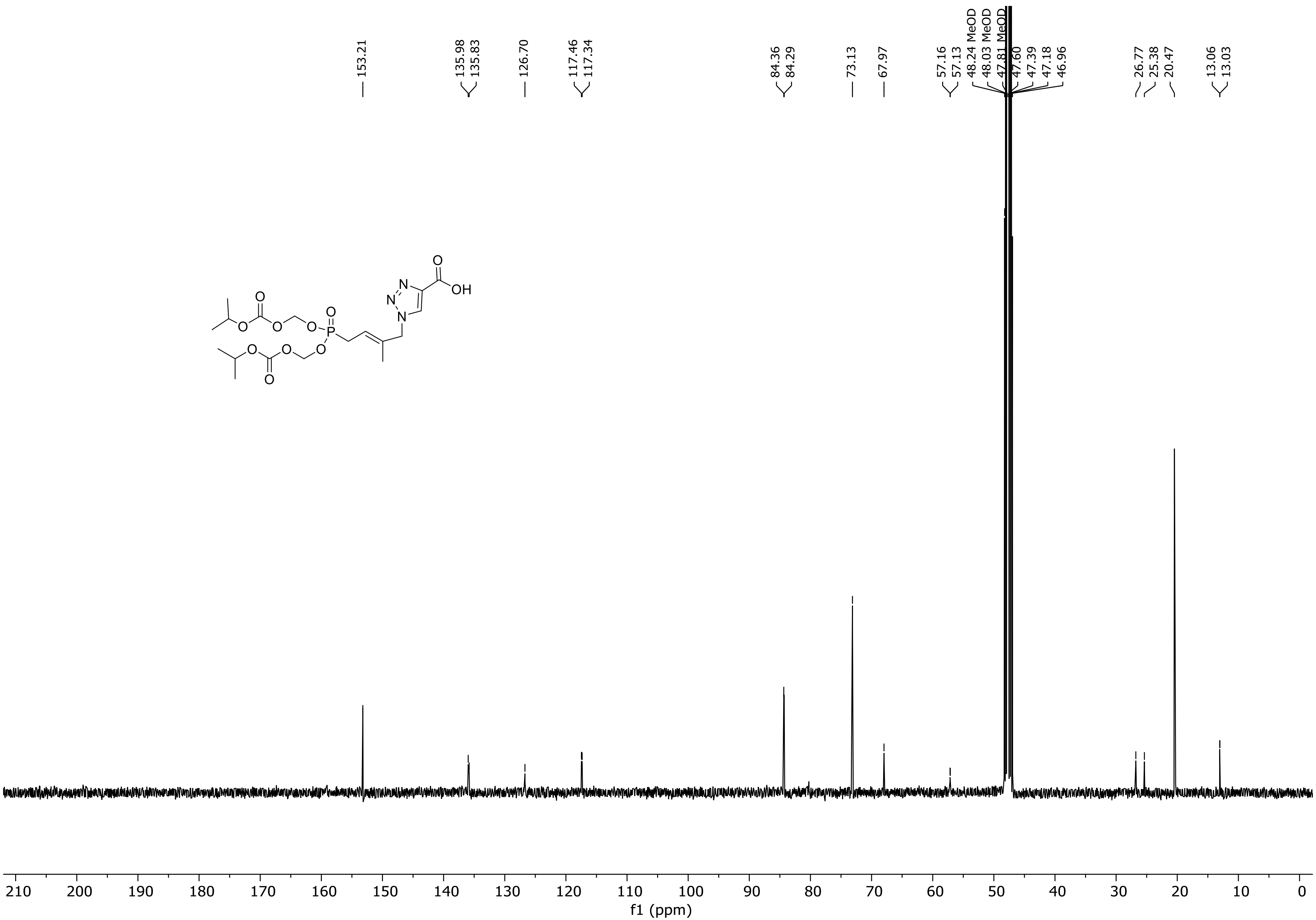

^{31}P NMR (161 MHz, MeOD) Analysis of Compound 11m

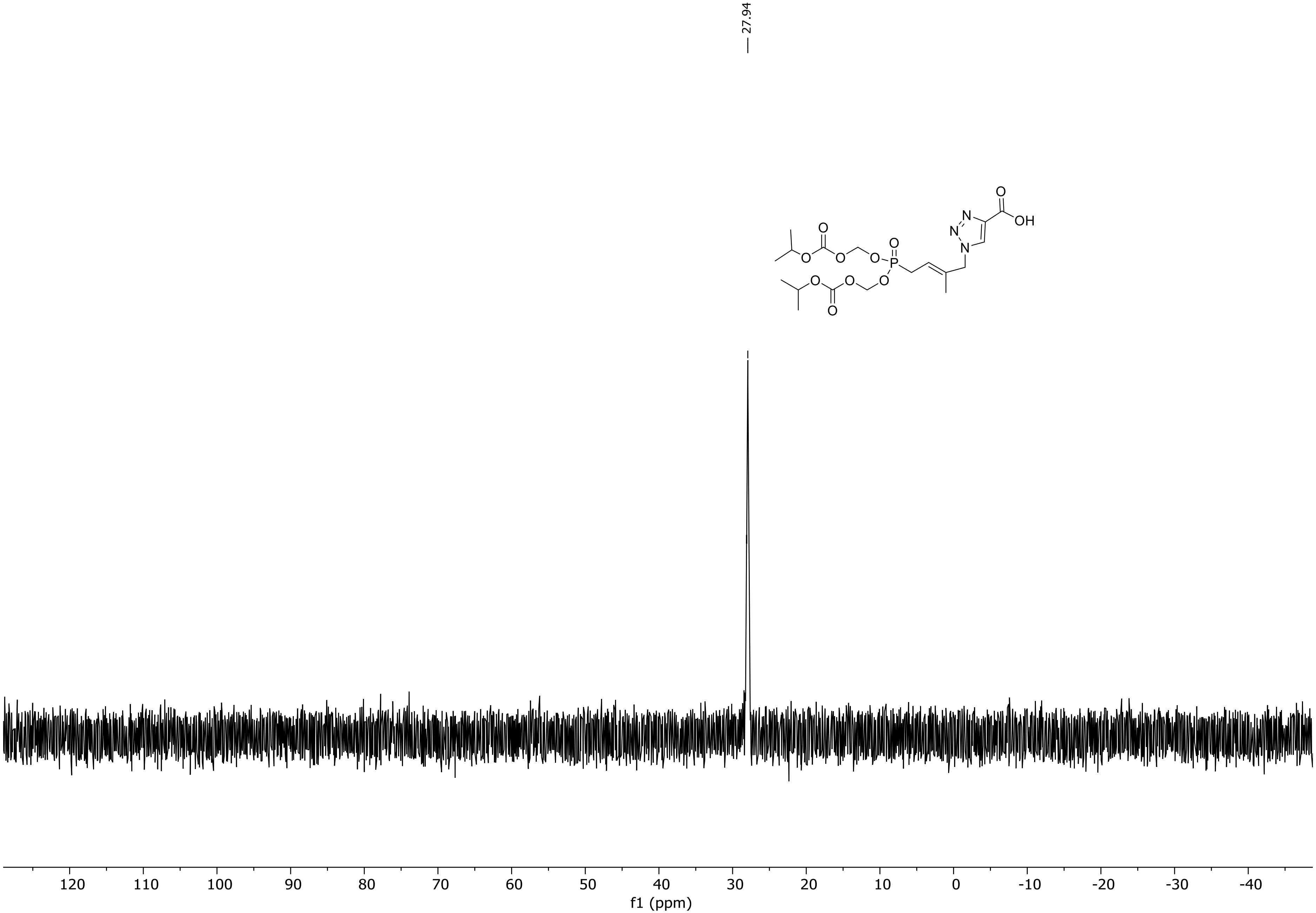

^{1}H NMR (250 MHz, MeOD) Analysis of Compound 11n

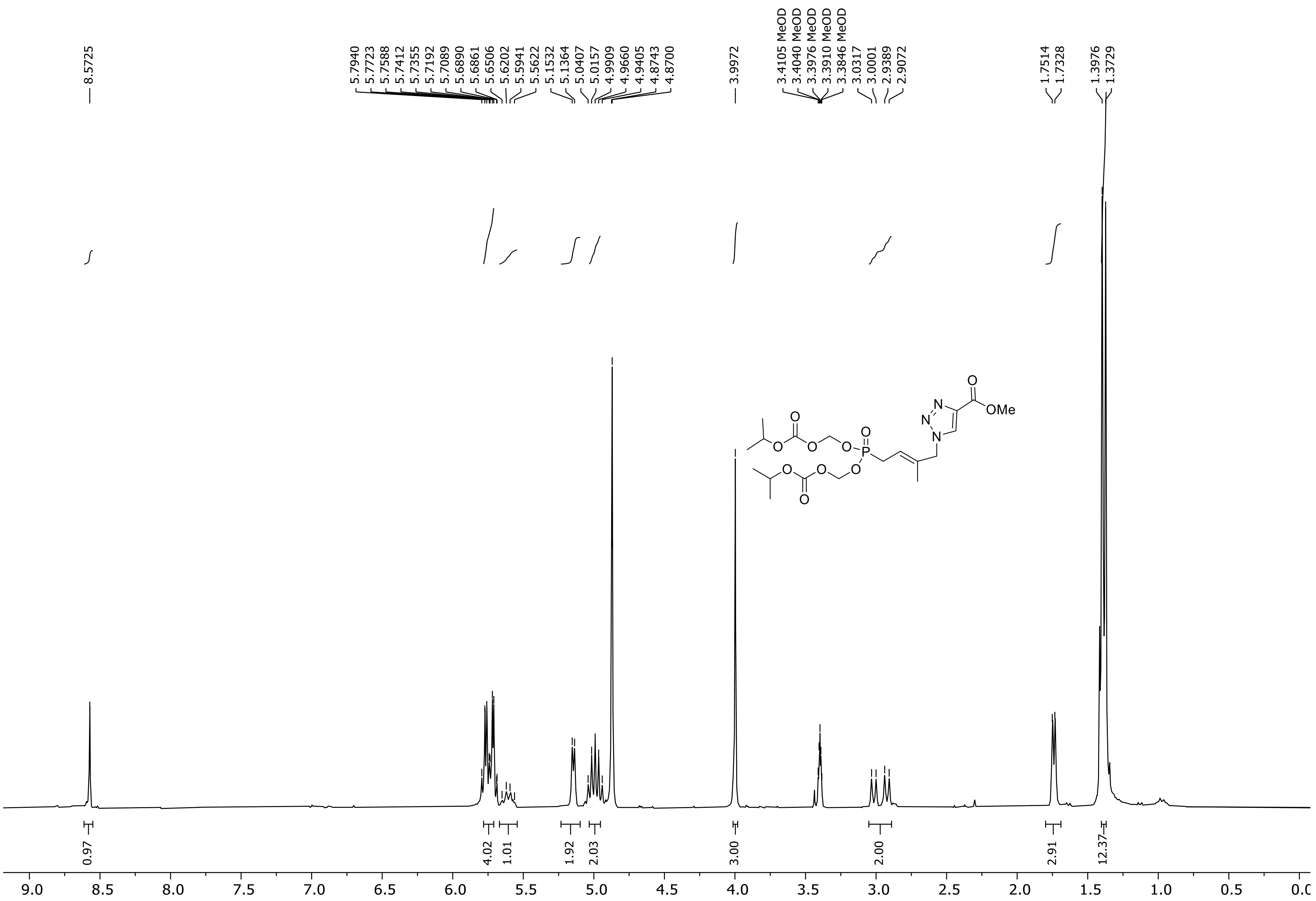

^{13}C NMR (101 MHz, $CDCl_3$) Analysis of Compound 11n

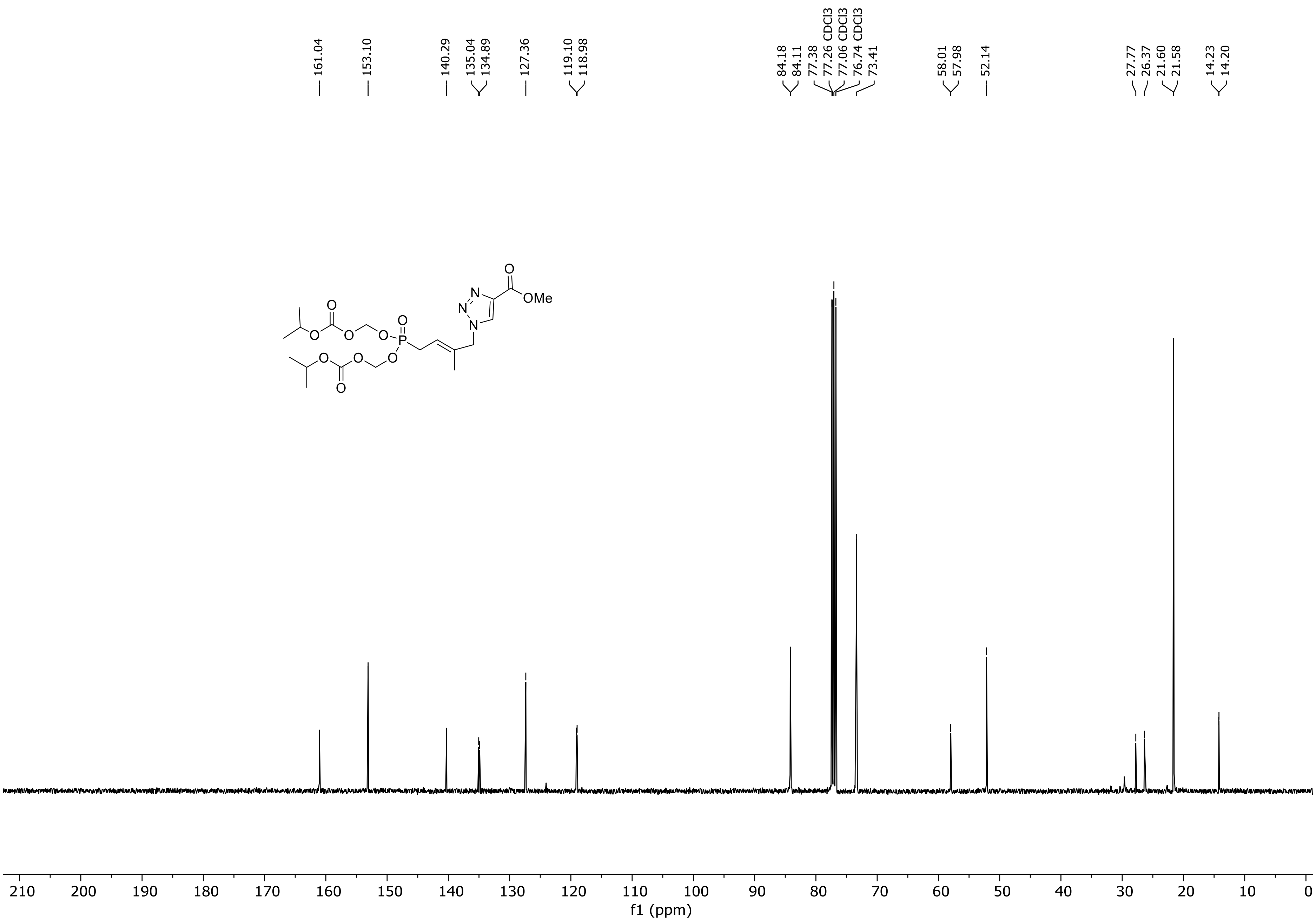

^{31}P NMR (161 MHz, $CDCl_3$) Analysis of Compound 11n

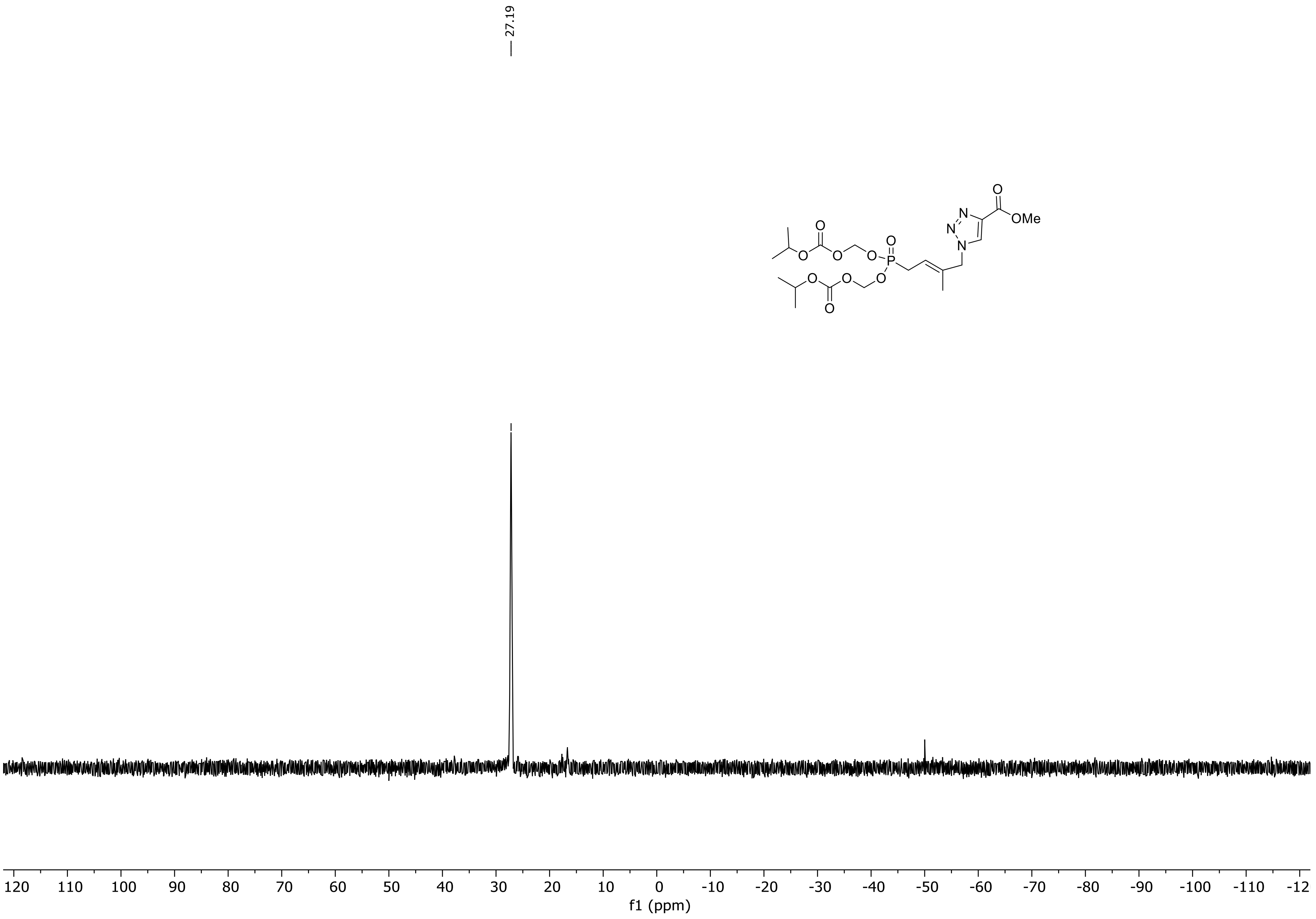

^{1}H NMR (400 MHz, MeOD) Analysis of Compound 11o

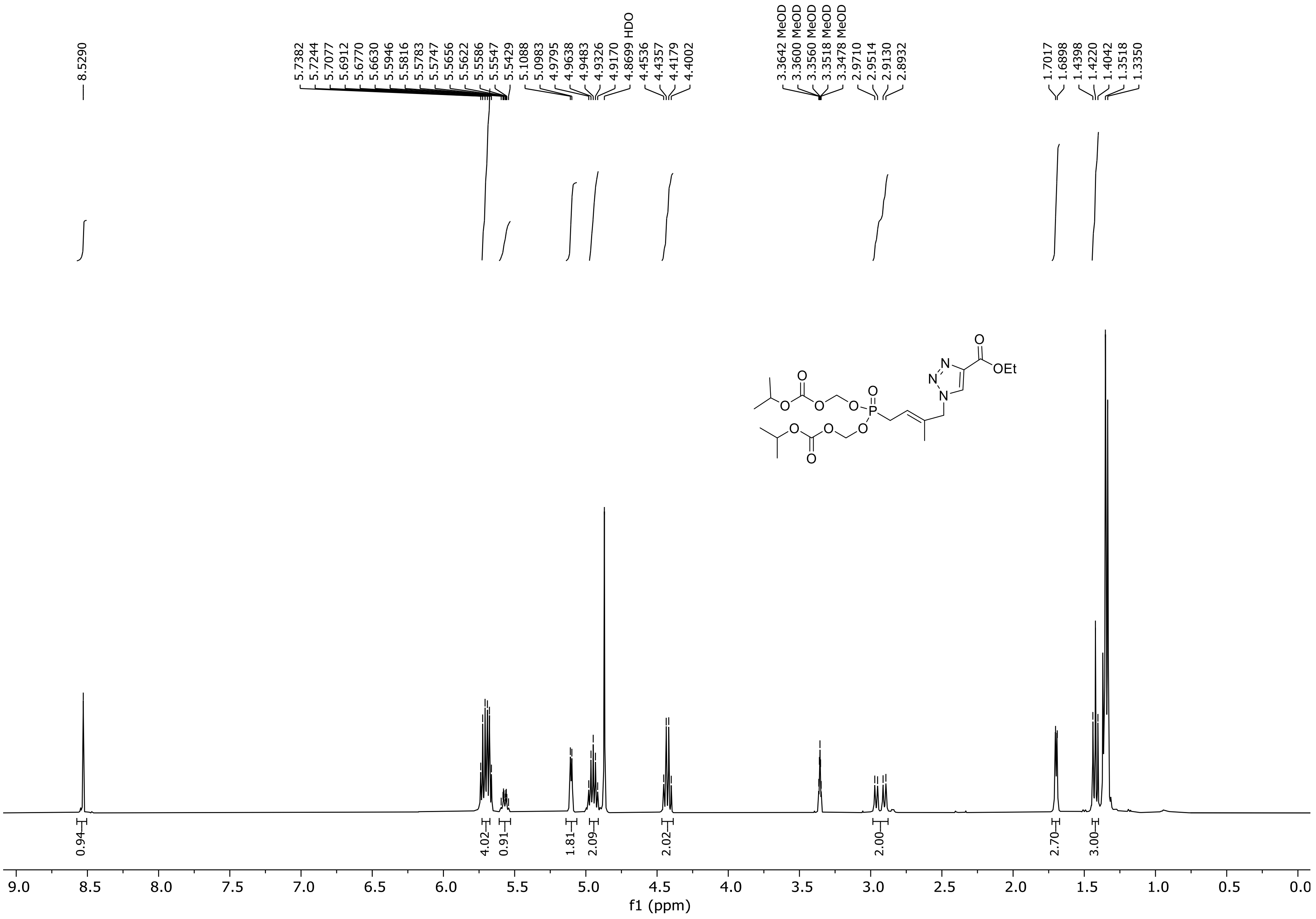

^{13}C NMR (101 MHz, MeOD) Analysis of Compound 11o

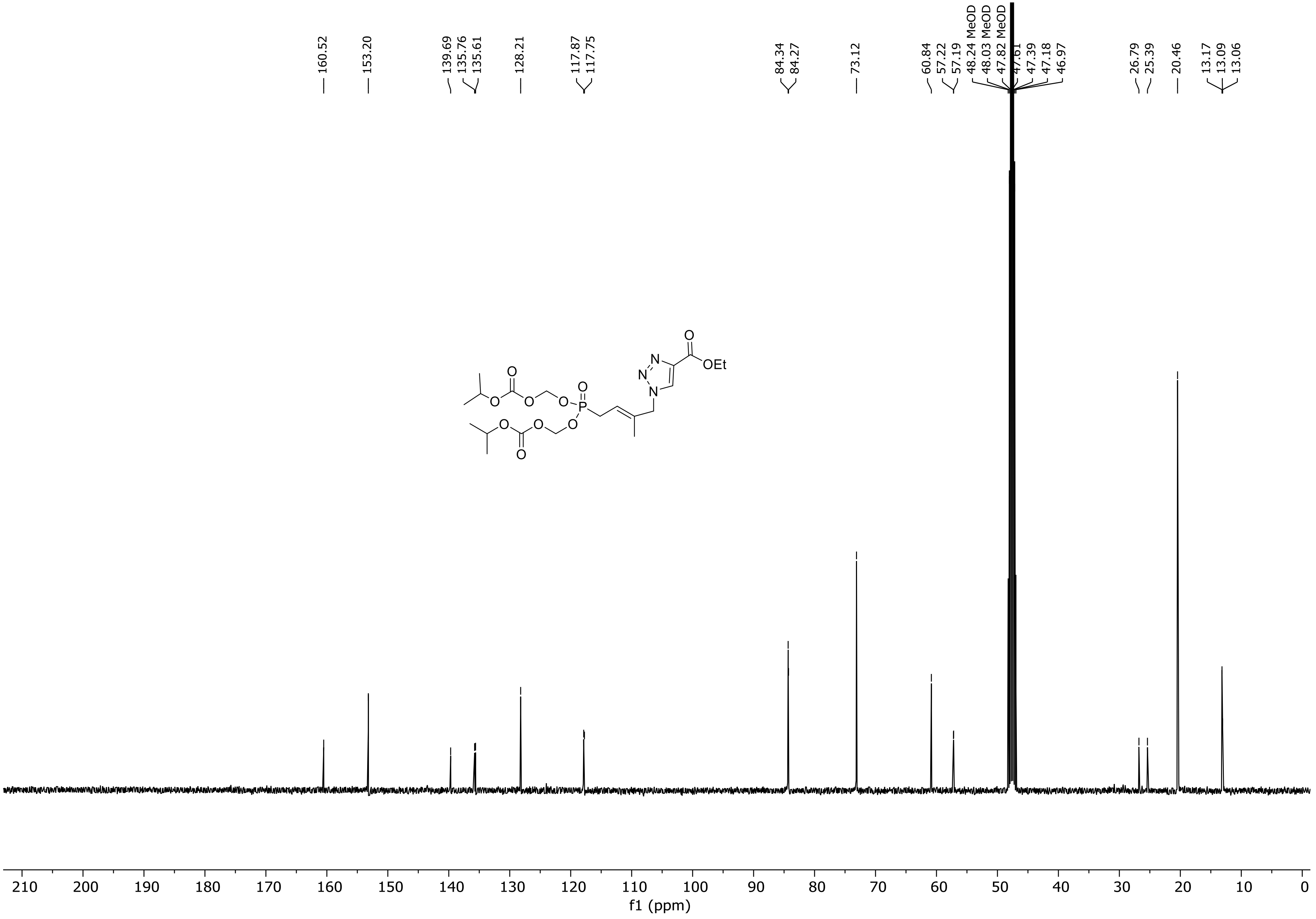

^{31}P NMR (161 MHz, MeOD) Analysis of Compound 11o

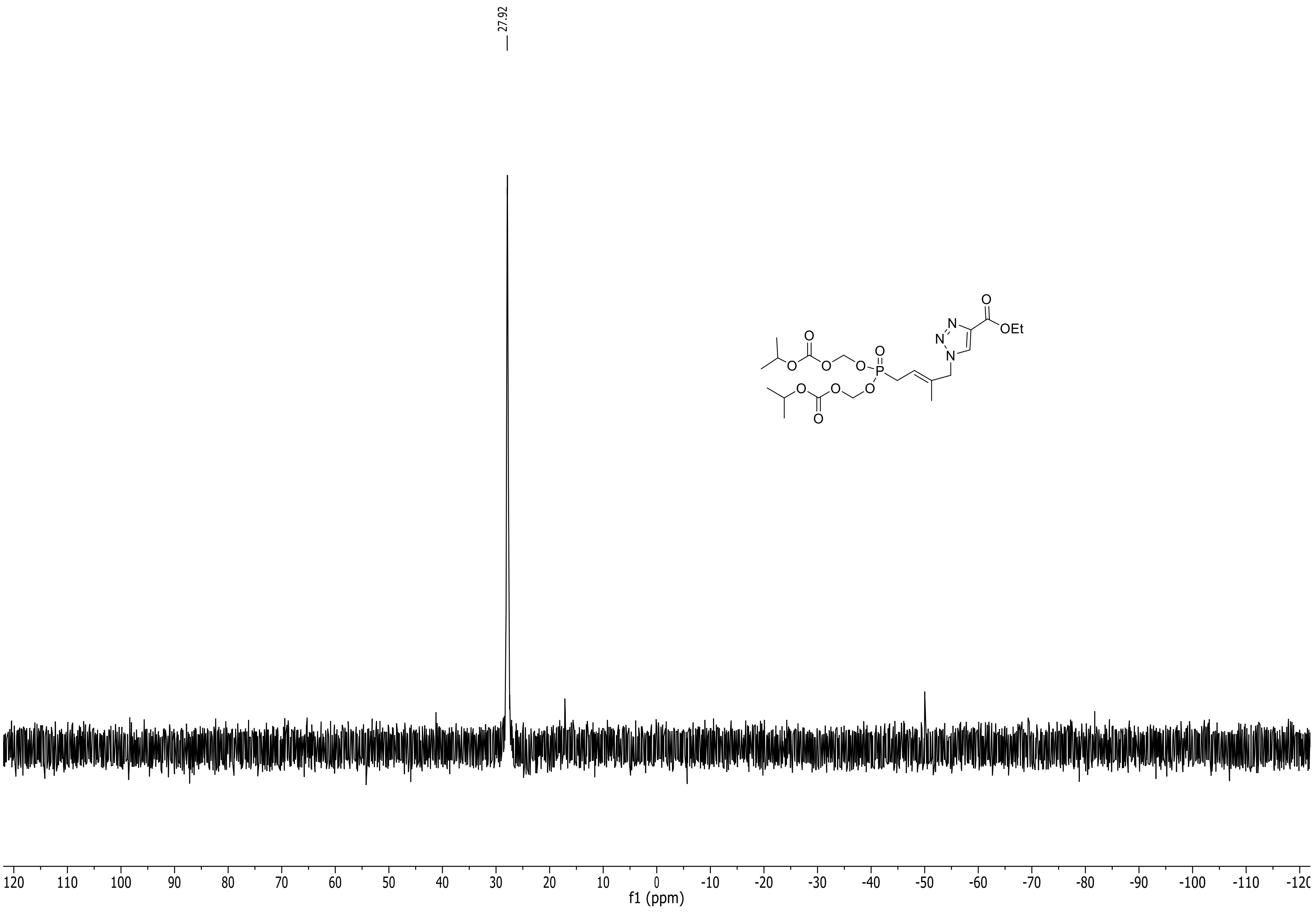

^{1}H NMR (400 MHz, MeOD) Analysis of Compound 11p

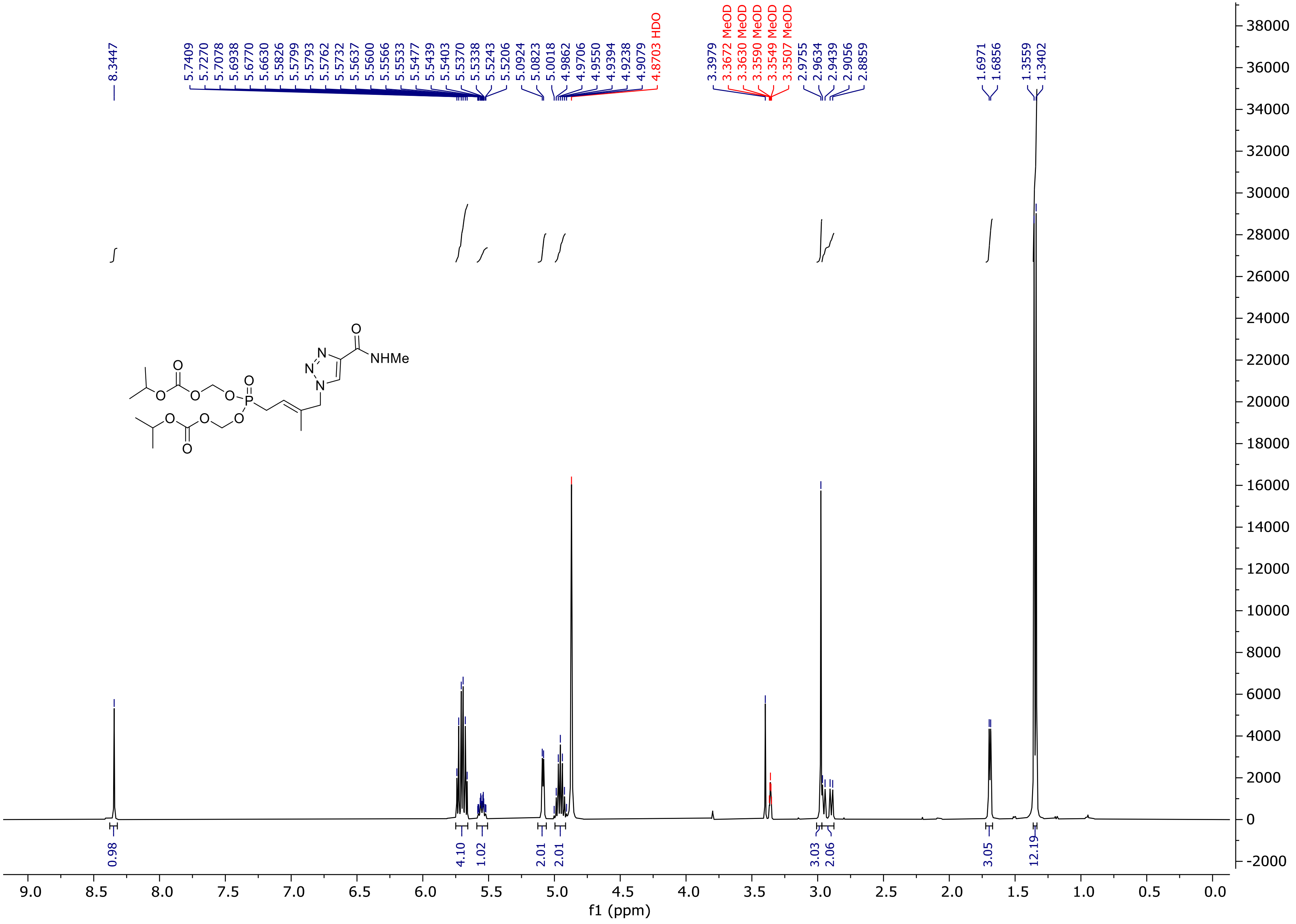

^{13}C NMR (101 MHz, MeOD) Analysis of Compound 11p

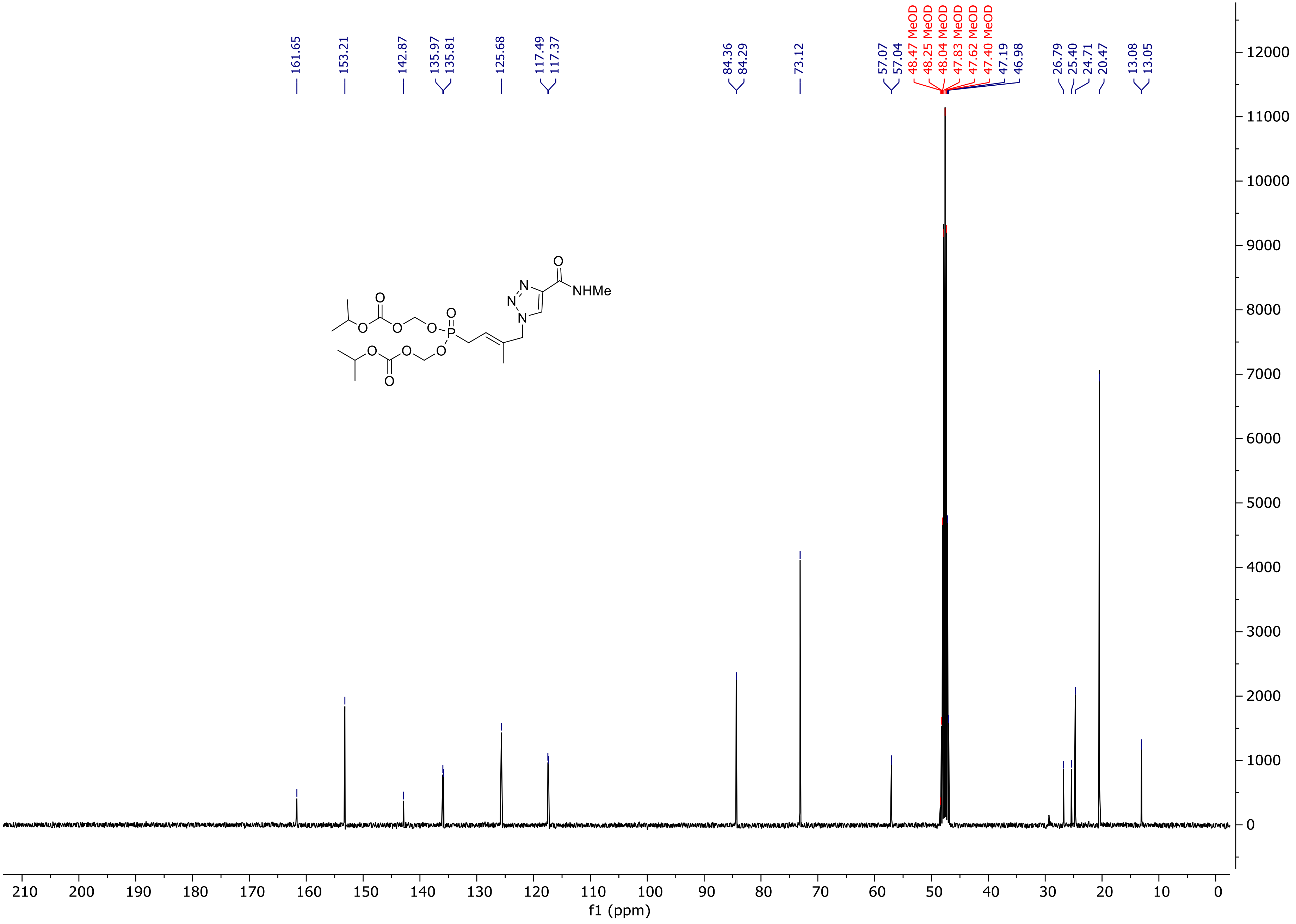

^{31}P NMR (161 MHz, MeOD) Analysis of Compound 11p

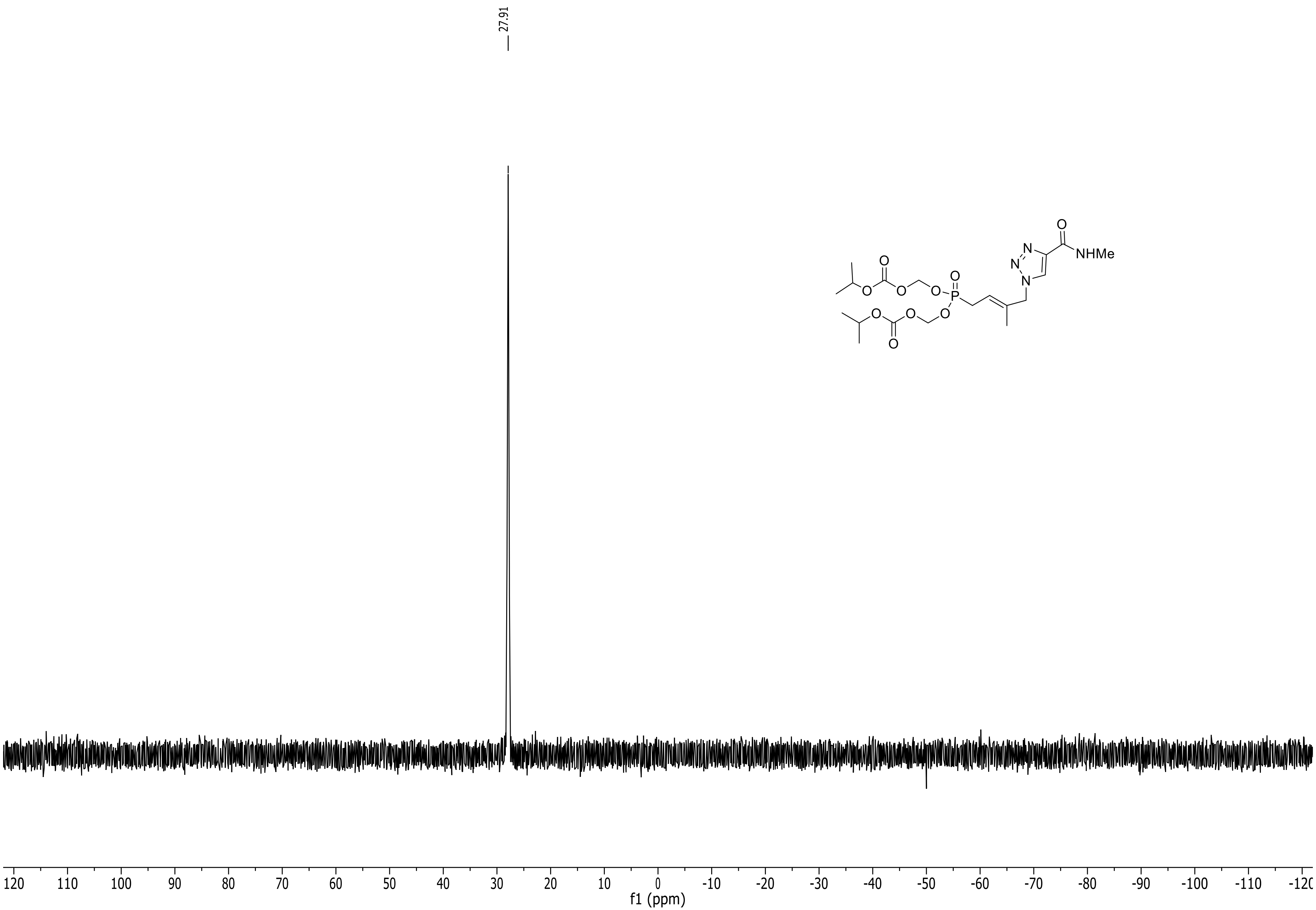

^{1}H NMR (400 MHz, MeOD) Analysis of Compound 11q

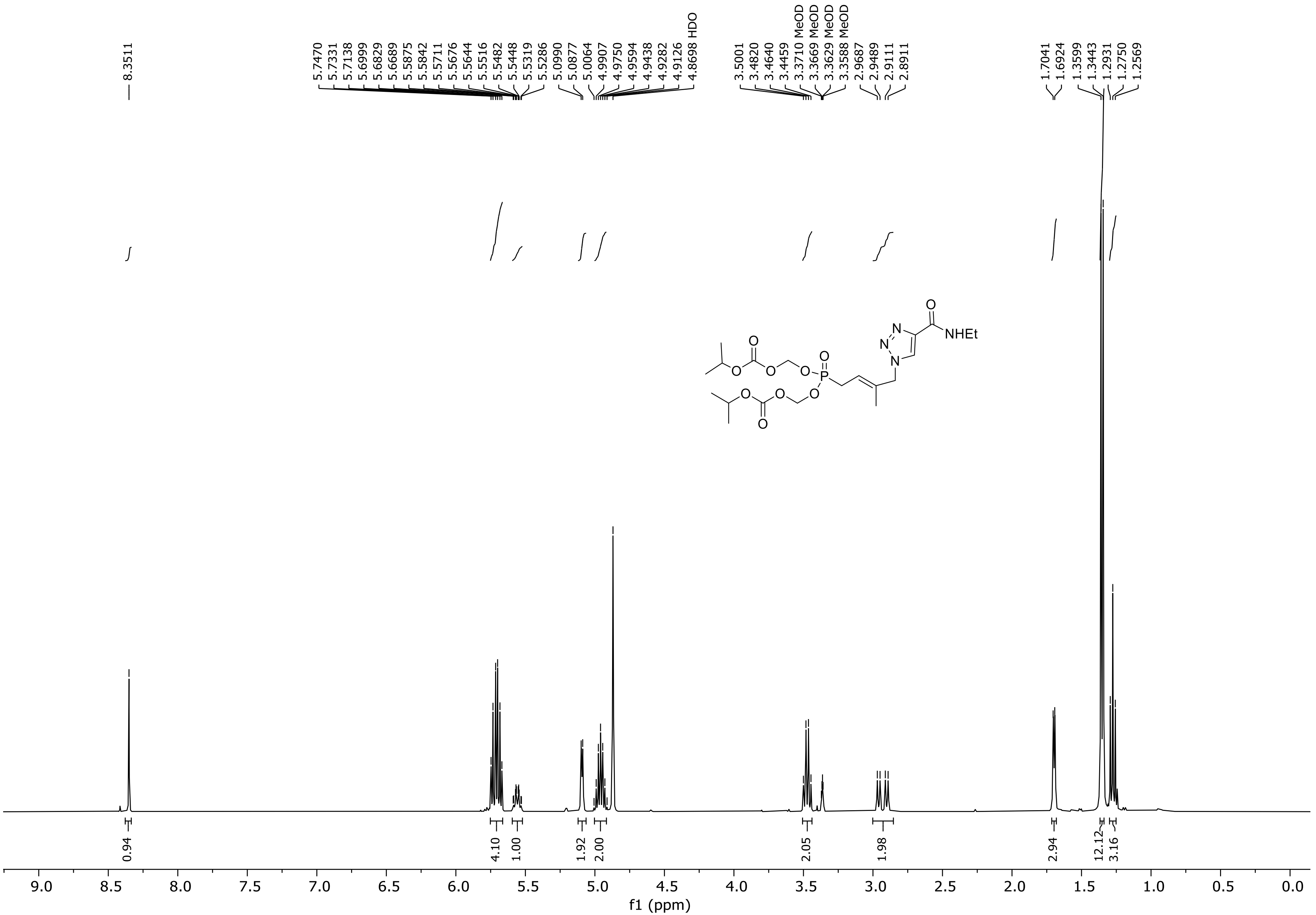

^{13}C NMR (101 MHz, MeOD) Analysis of Compound 11q

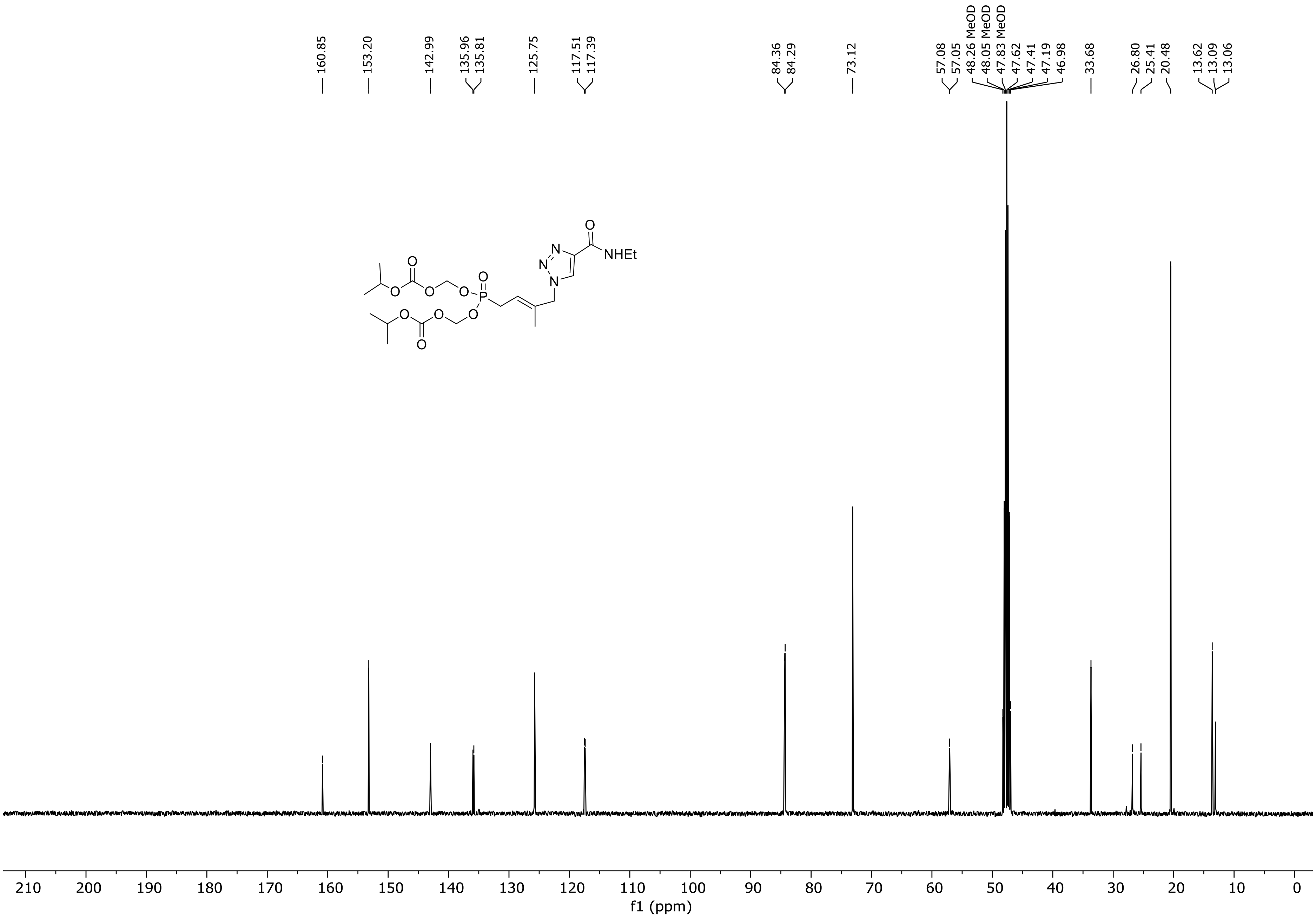

^{31}P NMR (161 MHz, MeOD) Analysis of Compound 11q

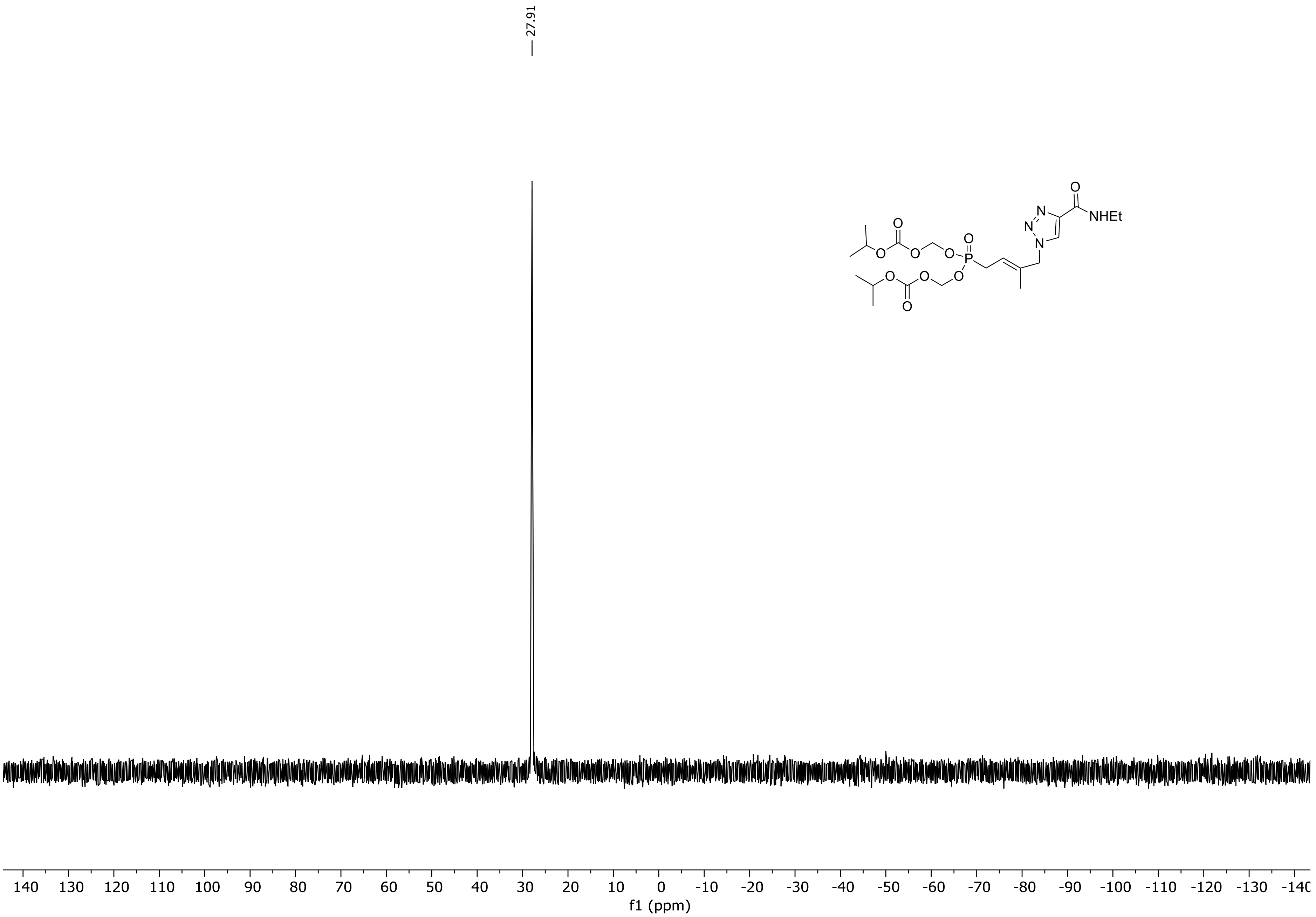

^{1}H NMR (400 MHz, $CDCl_3$) Analysis of Compound 13

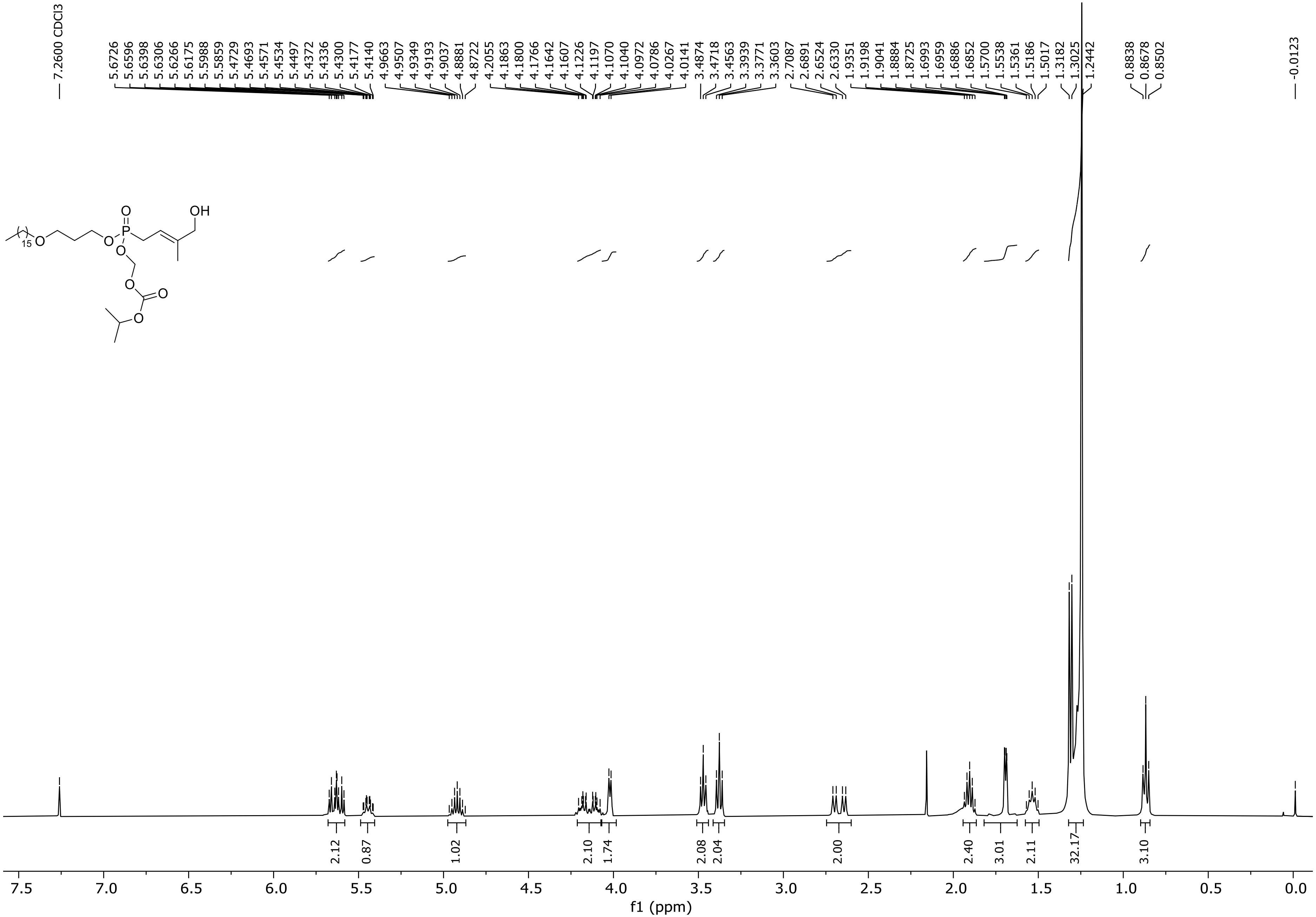

^{13}C NMR (101 MHz, CDCl$_3$) Analysis of Compound 13

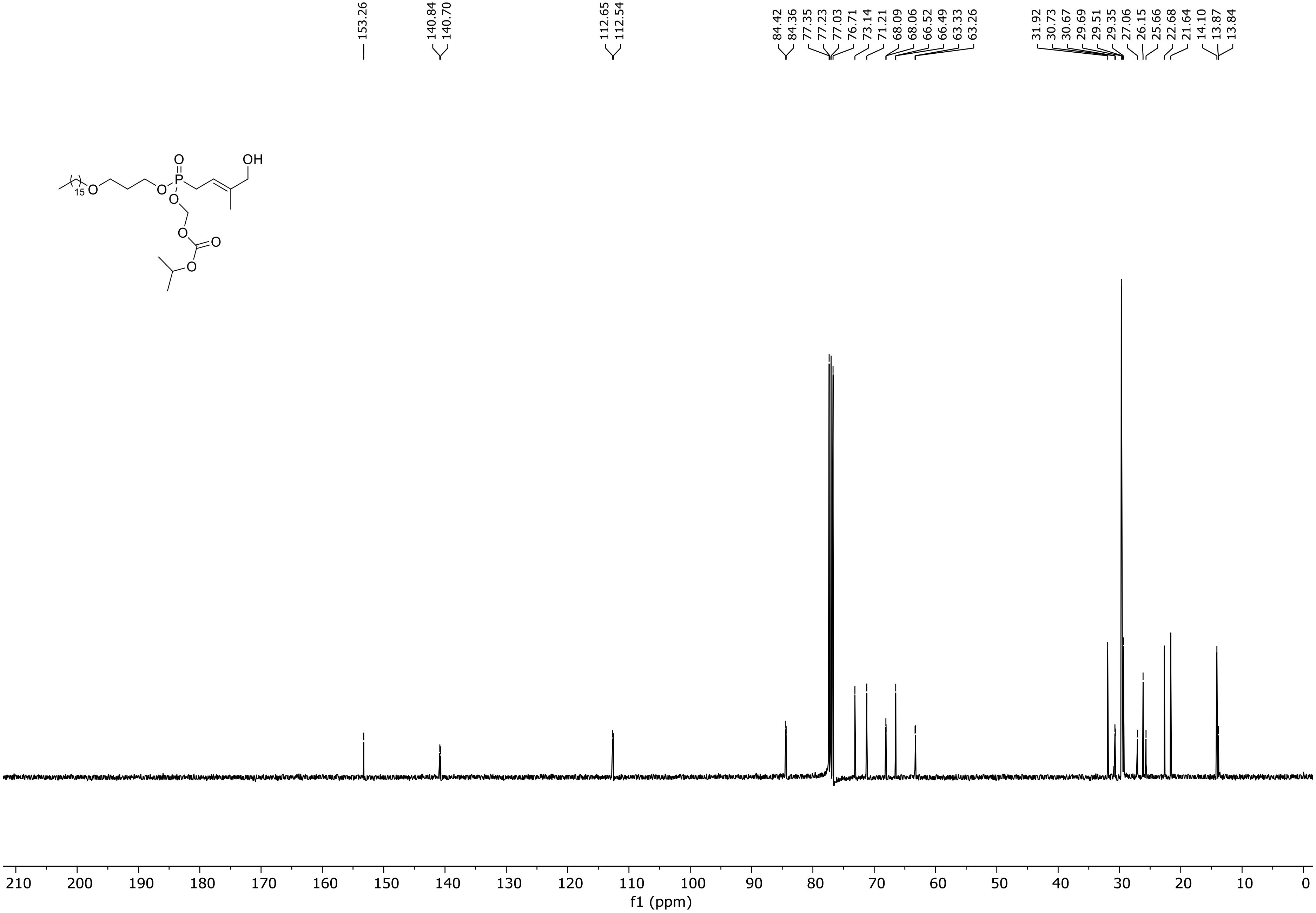

^{1}H NMR (400 MHz, $CDCl_3$) Analysis of Compound 14

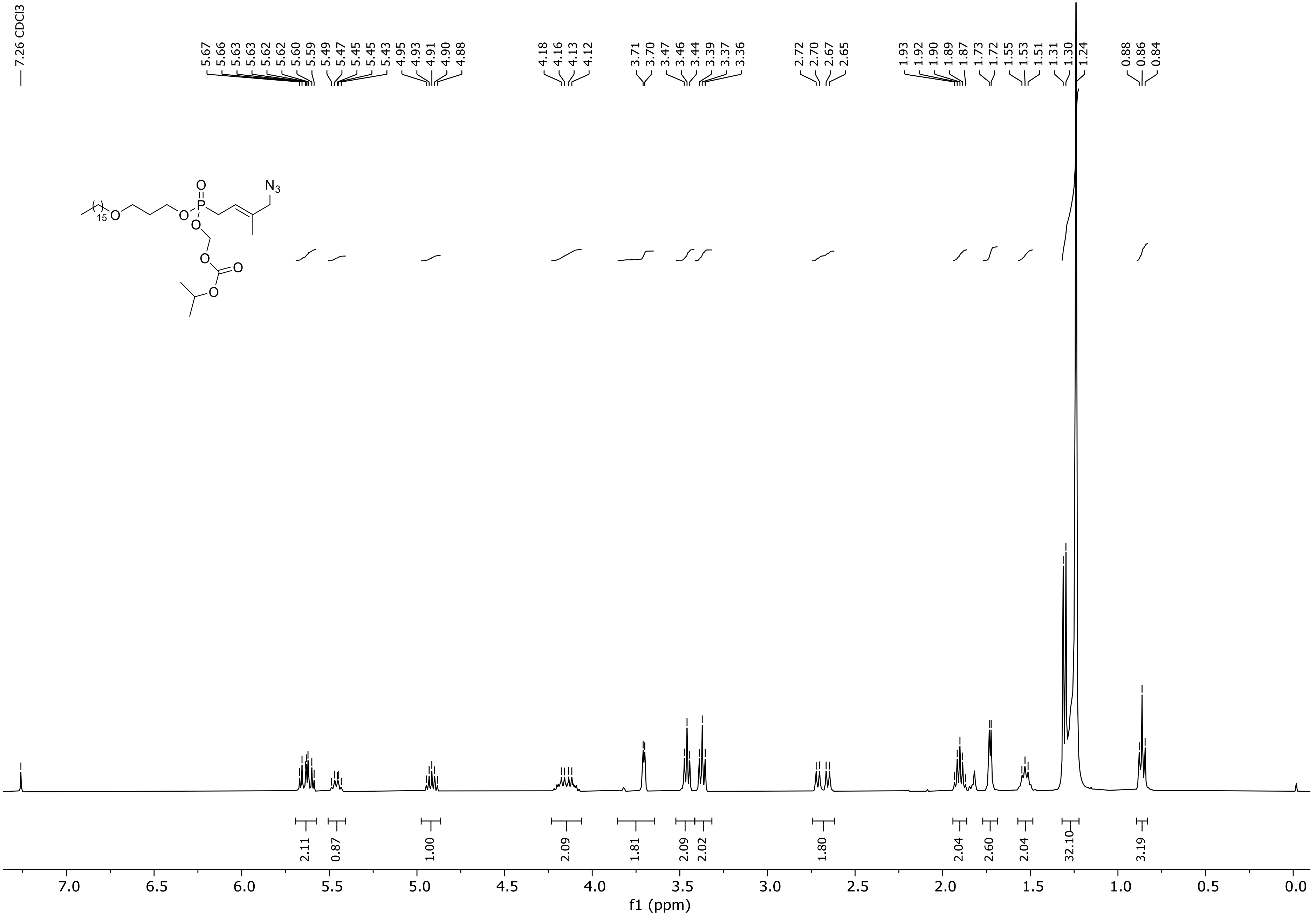

^{13}C NMR (101 MHz, CDCl$_3$) Analysis of Compound 14

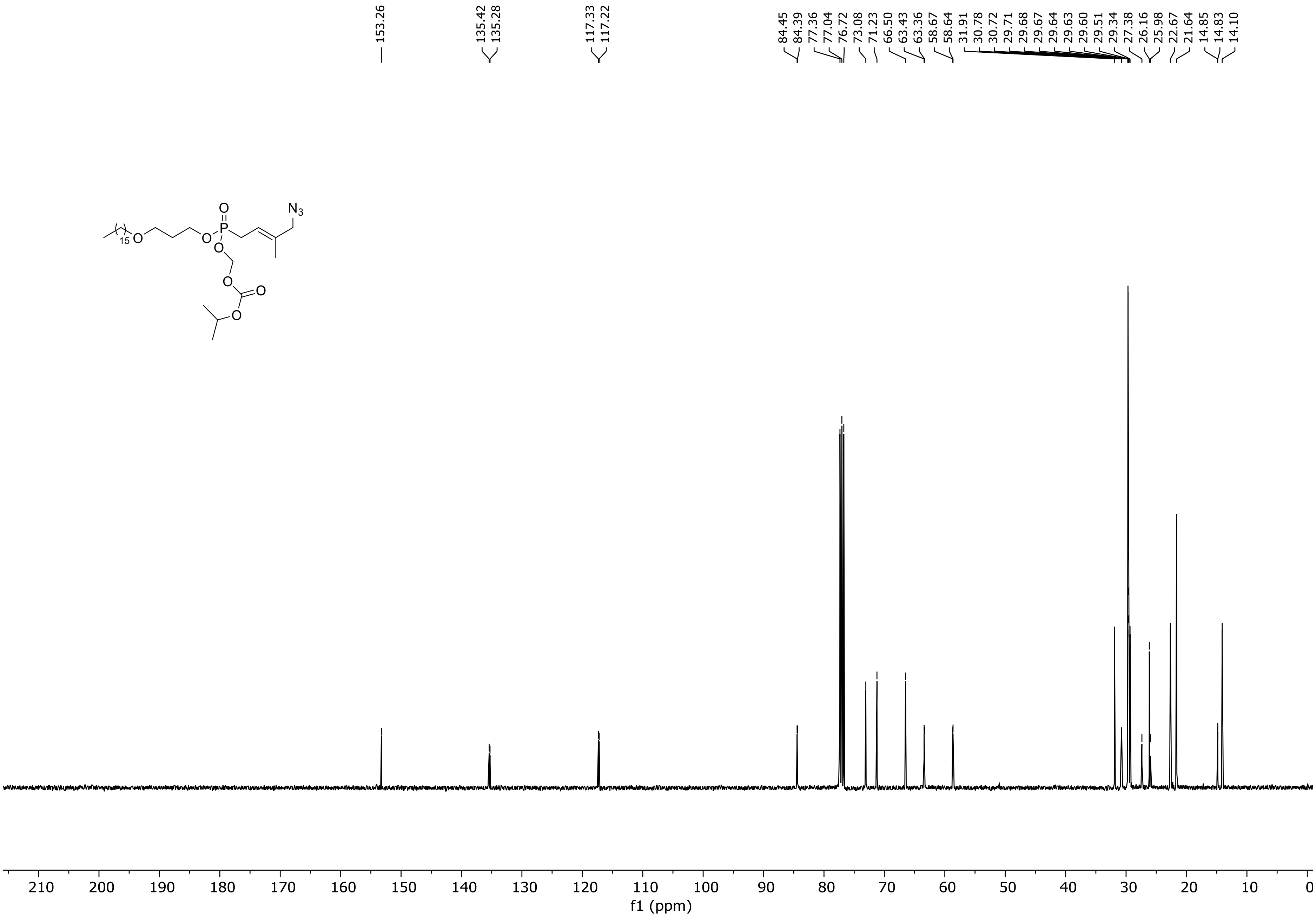

^{31}P NMR (161 MHz, $CDCl_3$) Analysis of Compound 14

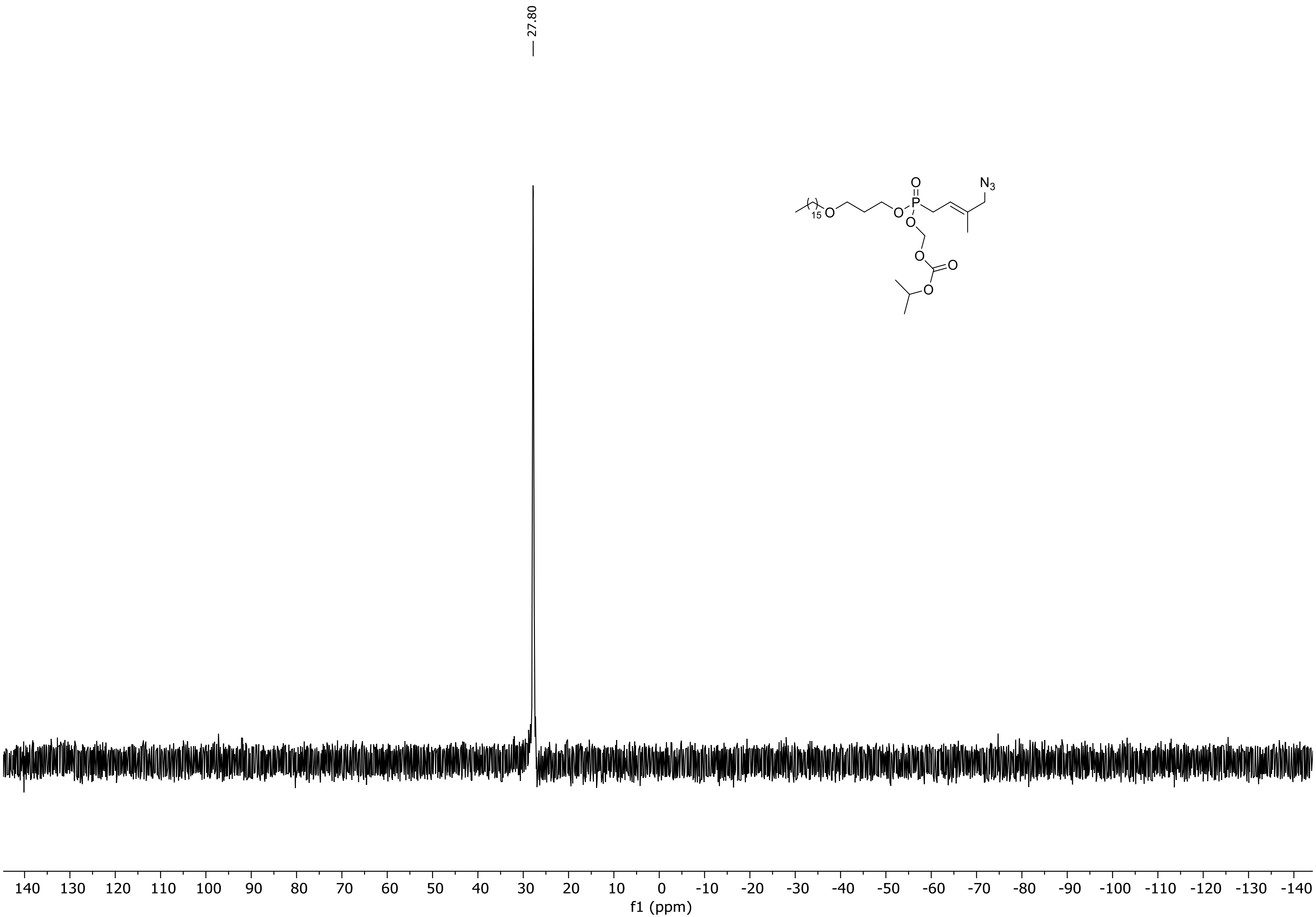

NOESY of Compound 14

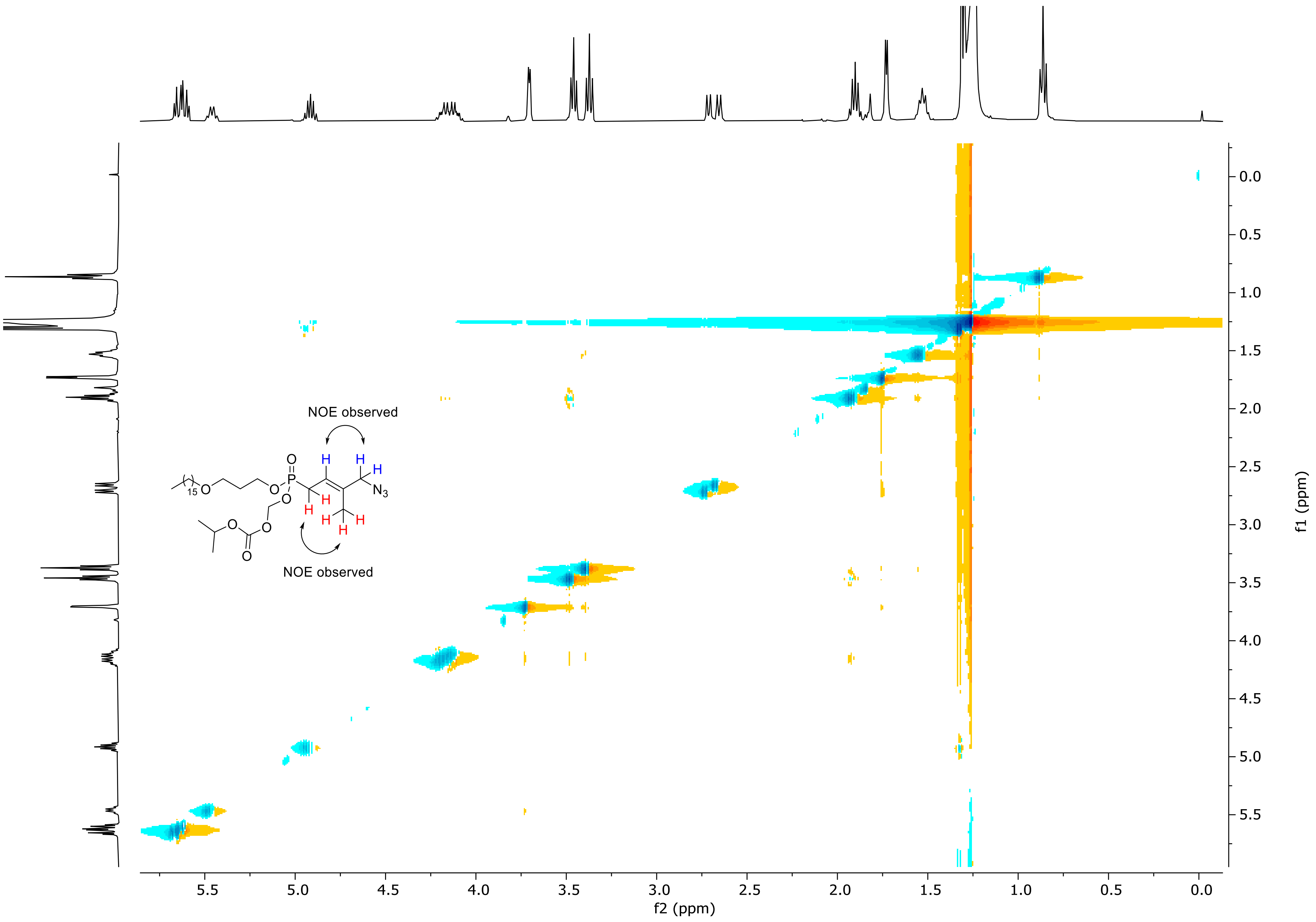

^{1}H NMR (400 MHz, $CDCl_3$) Analysis of Compound 15a

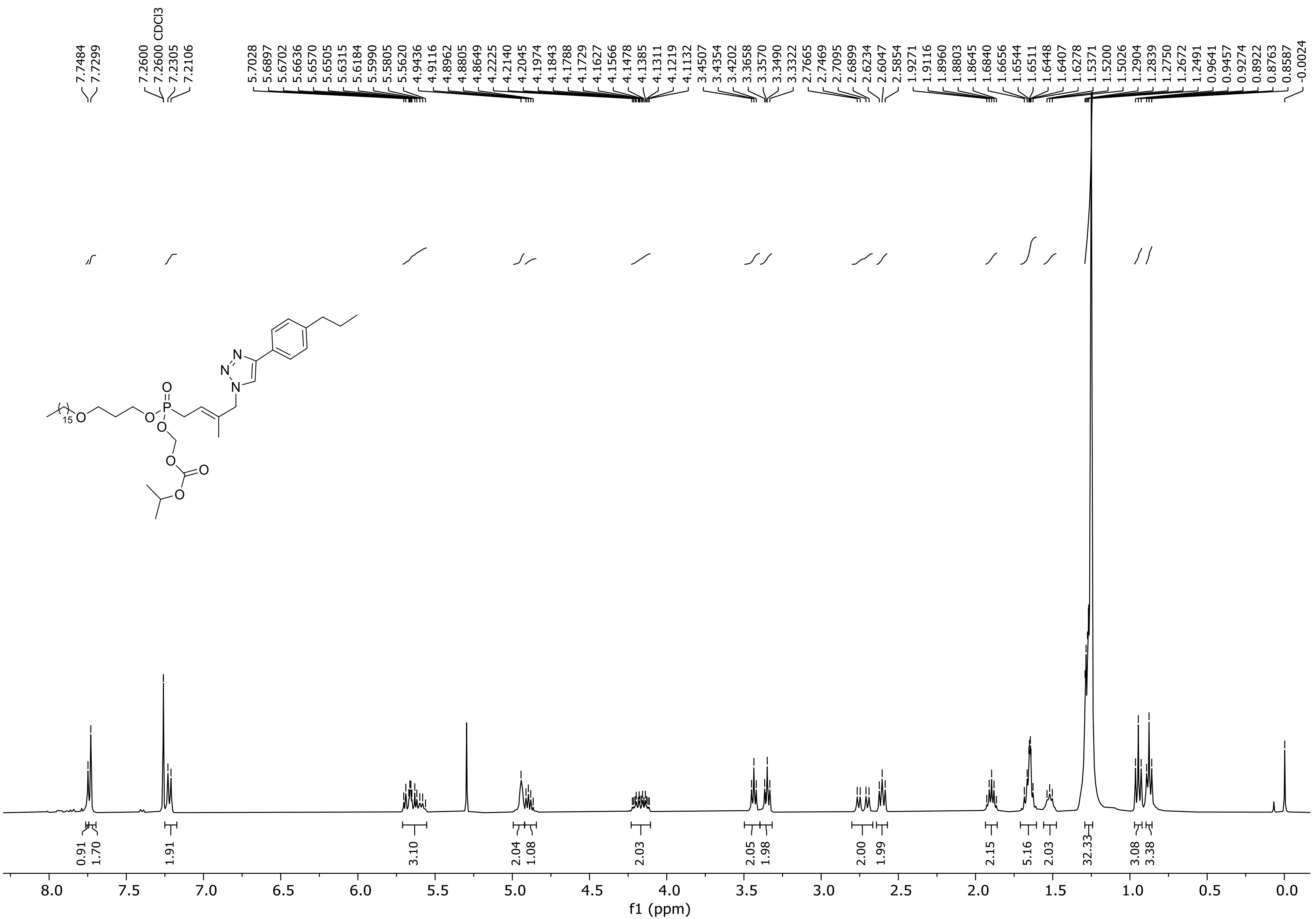

^{13}C NMR (101 MHz, $CDCl_3$) Analysis of Compound 15a

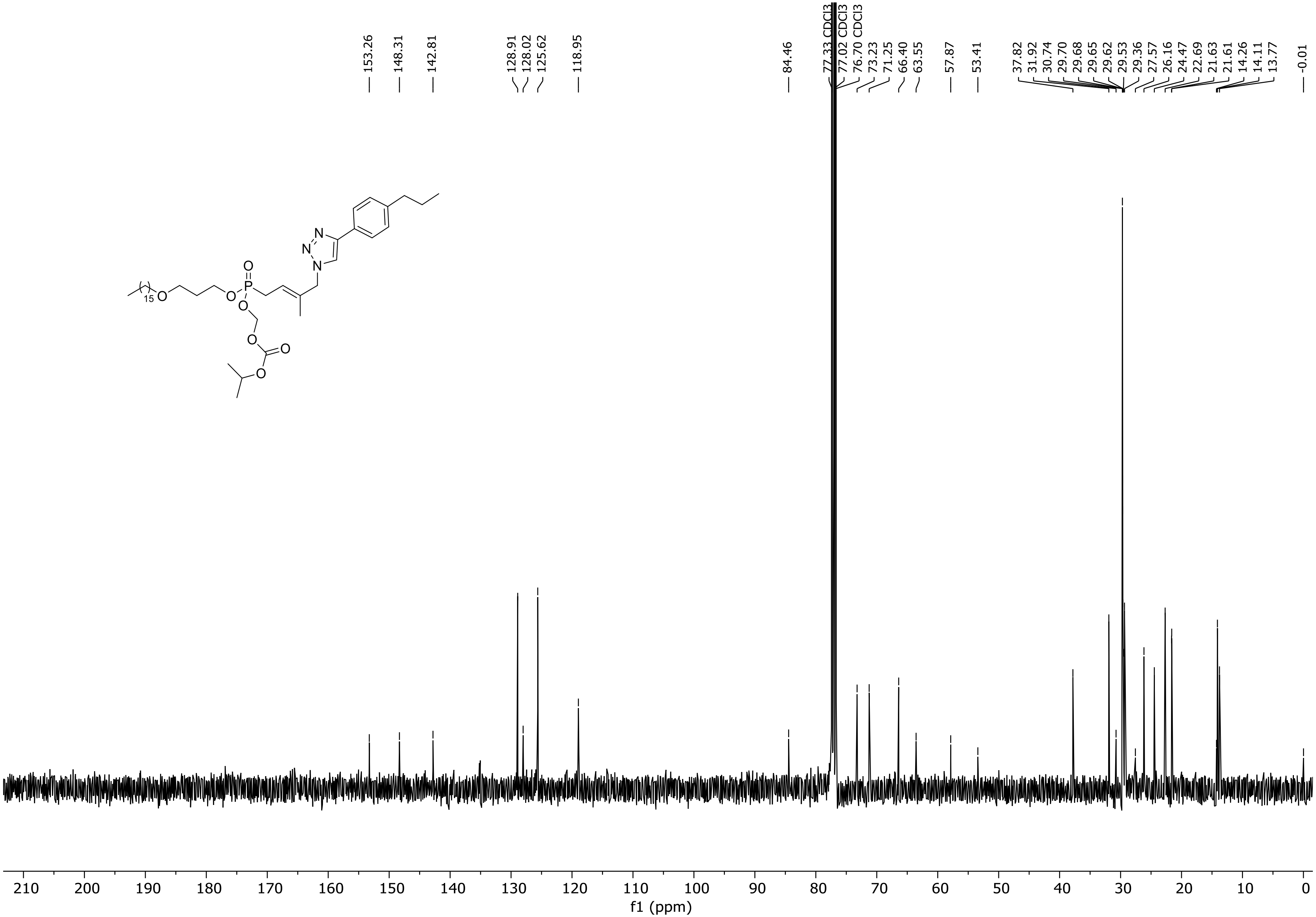

^{31}P NMR (161 MHz, $CDCl_3$) Analysis of Compound 15a

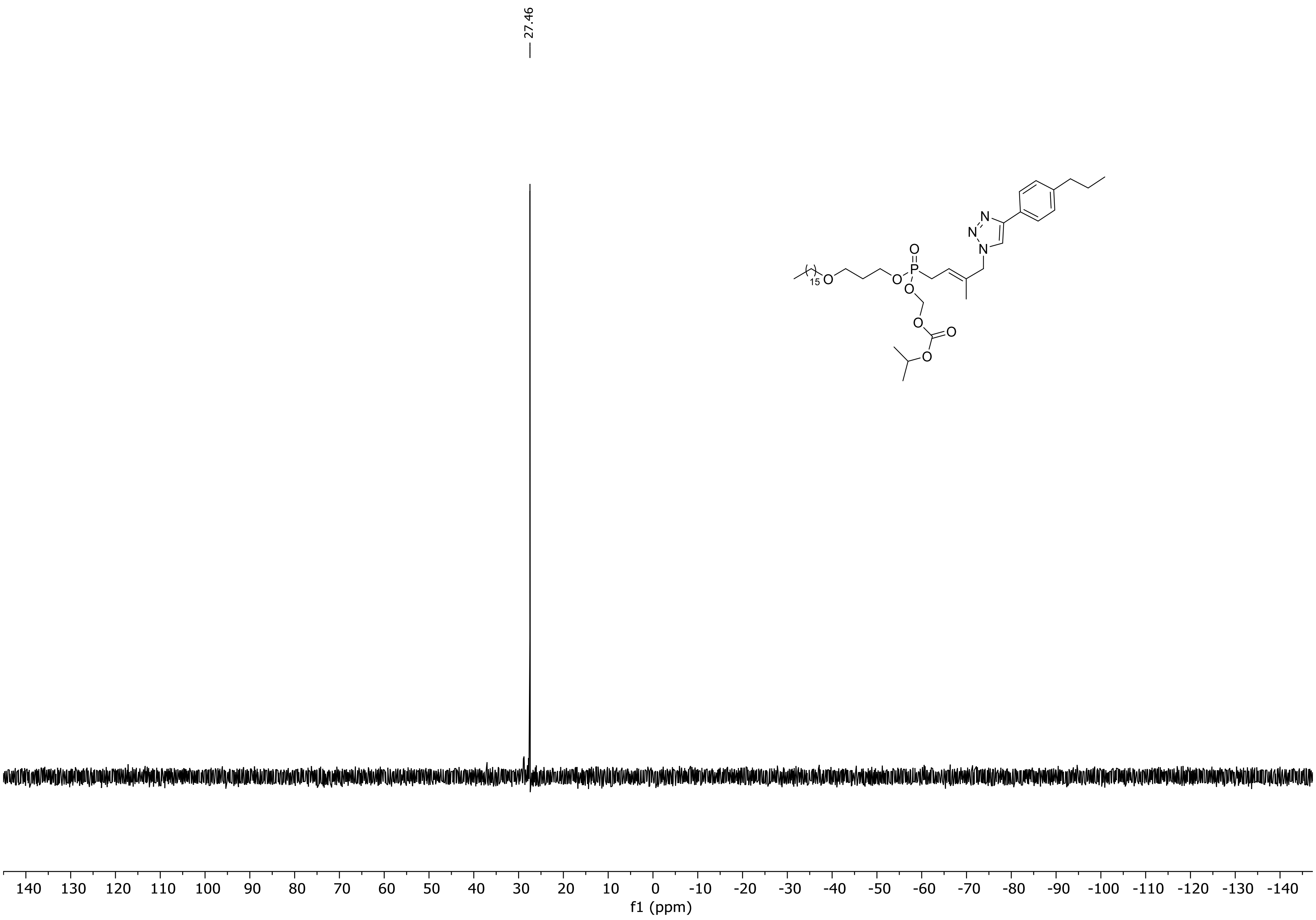

^1H NMR (400 MHz, $CDCl_3$) Analysis of Compound 15c

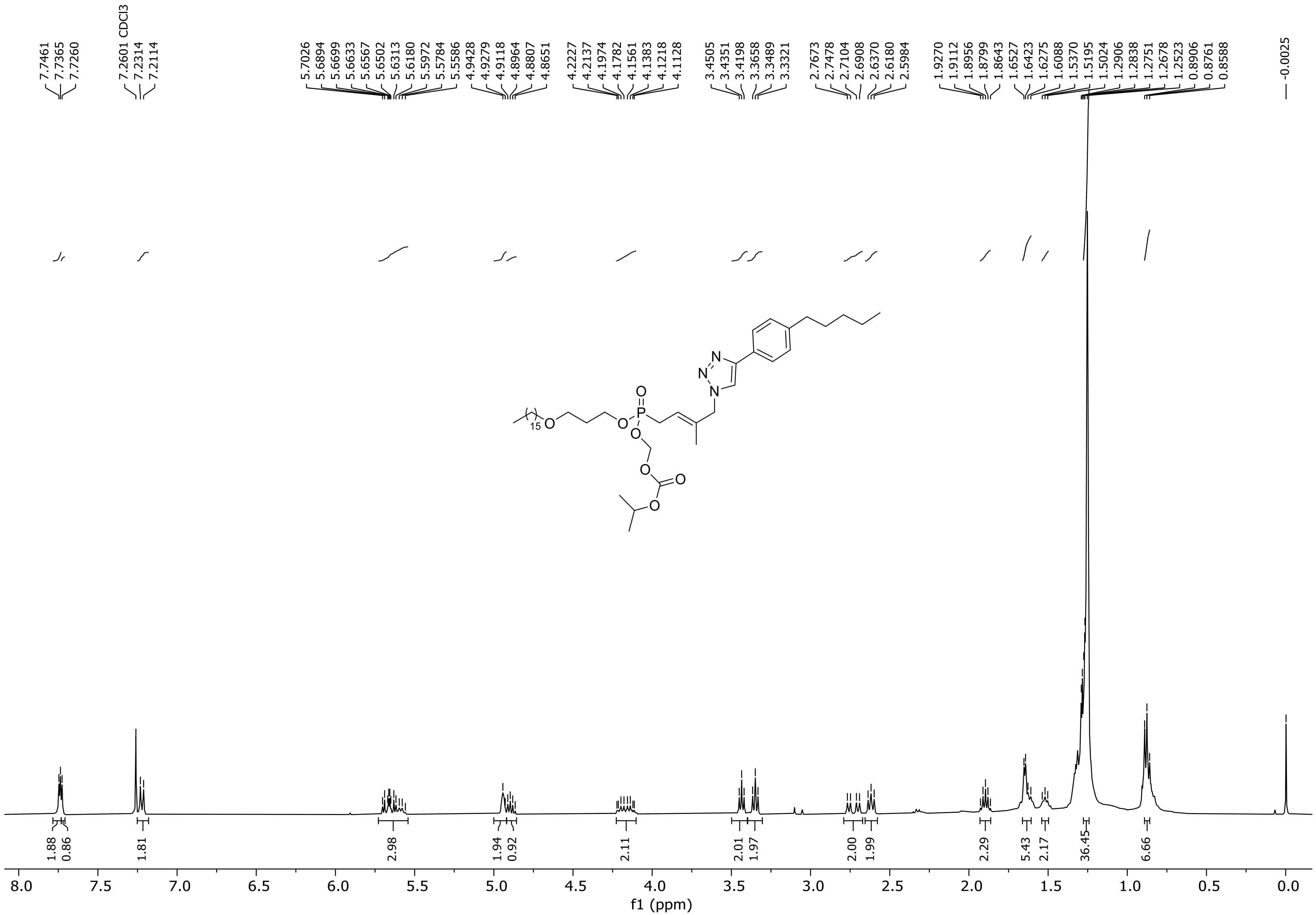

^{13}C NMR (101 MHz, $CDCl_3$) Analysis of Compound 15c

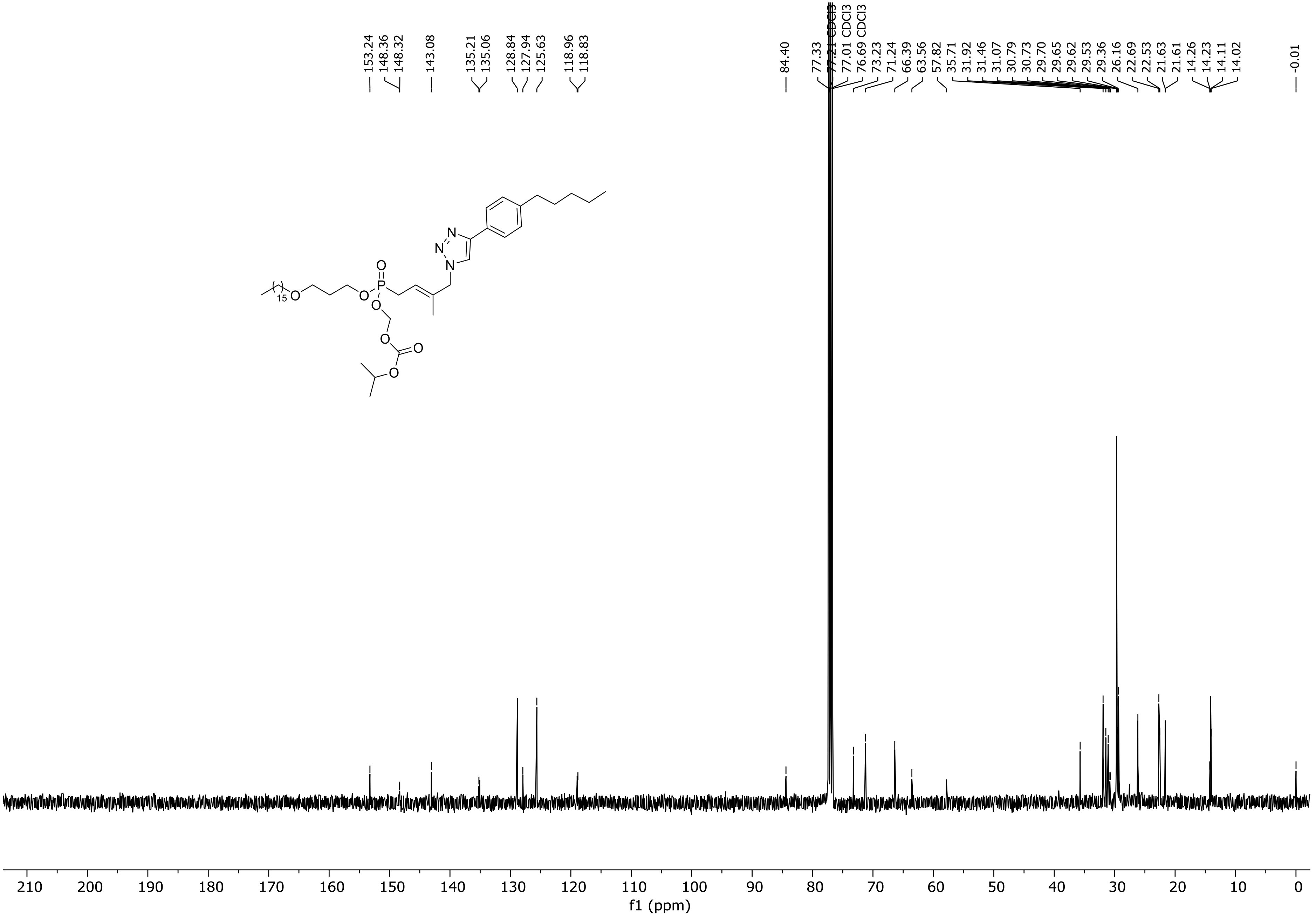

^{31}P NMR (161 MHz, $CDCl_3$) Analysis of Compound 15c

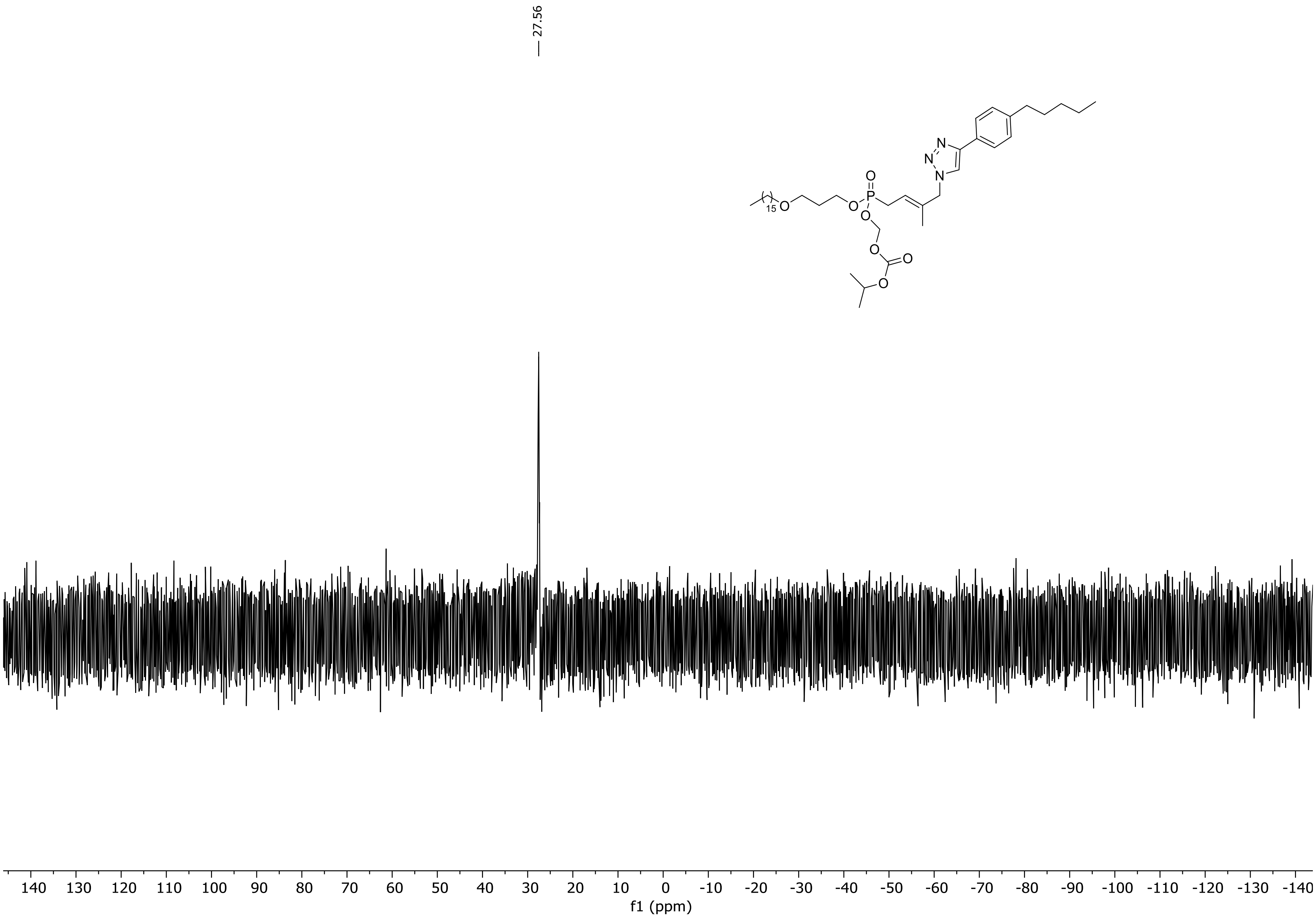

^{1}H NMR (400 MHz, MeOD) Analysis of Compound 15e

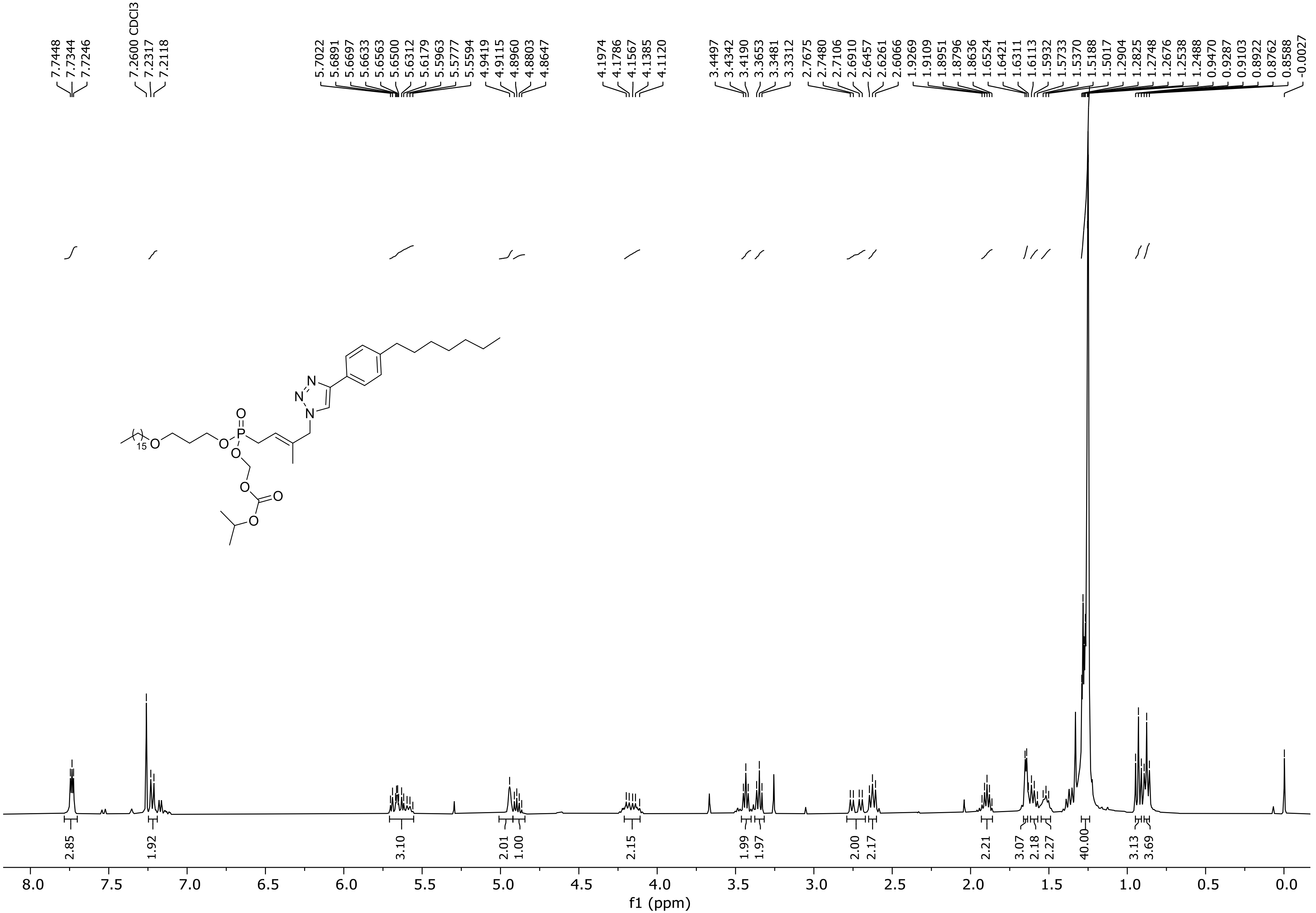

^{13}C NMR (101 MHz, $CDCl_3$) Analysis of Compound 15e

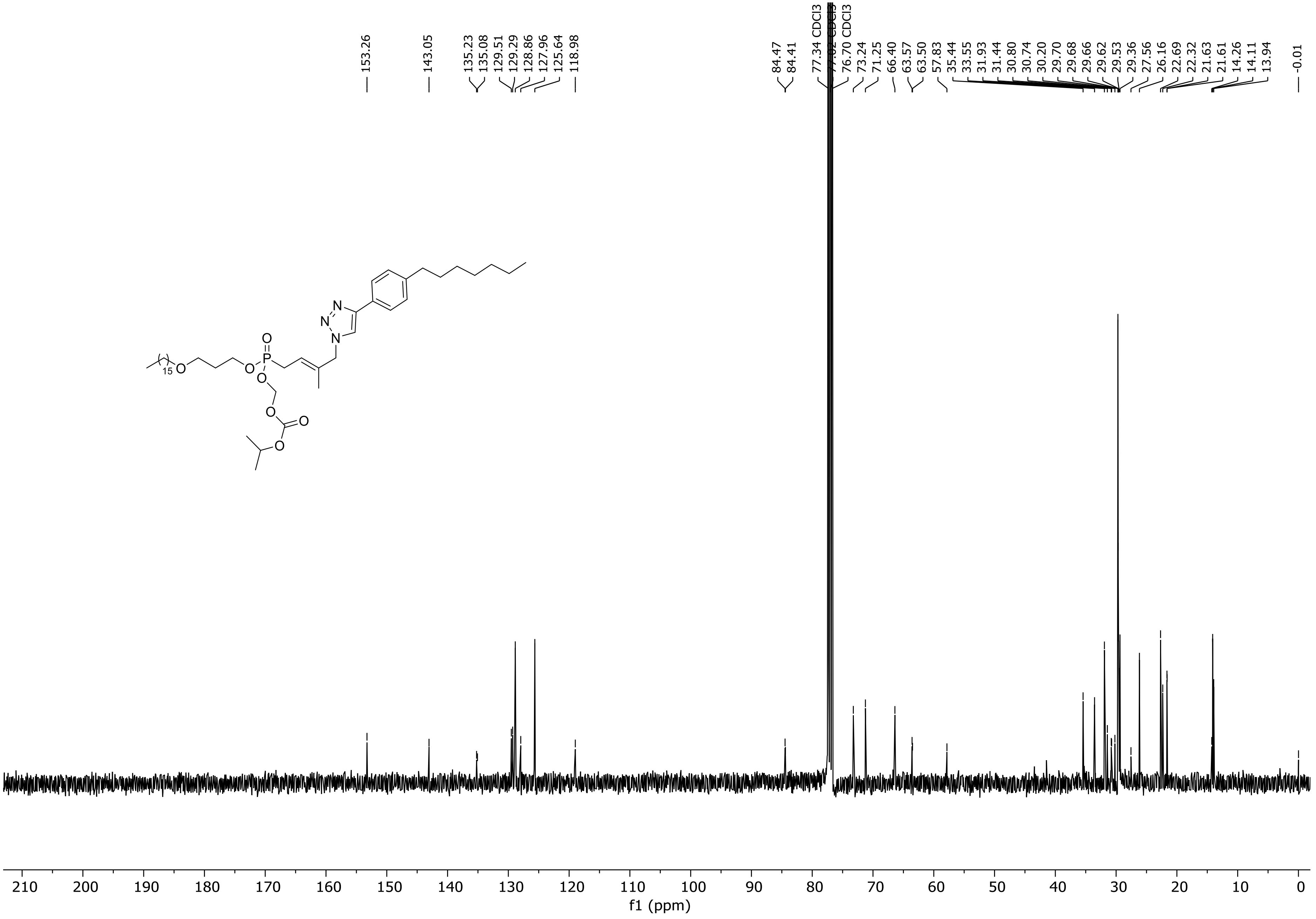

^{31}P NMR (161 MHz, $CDCl_3$) Analysis of Compound 15e

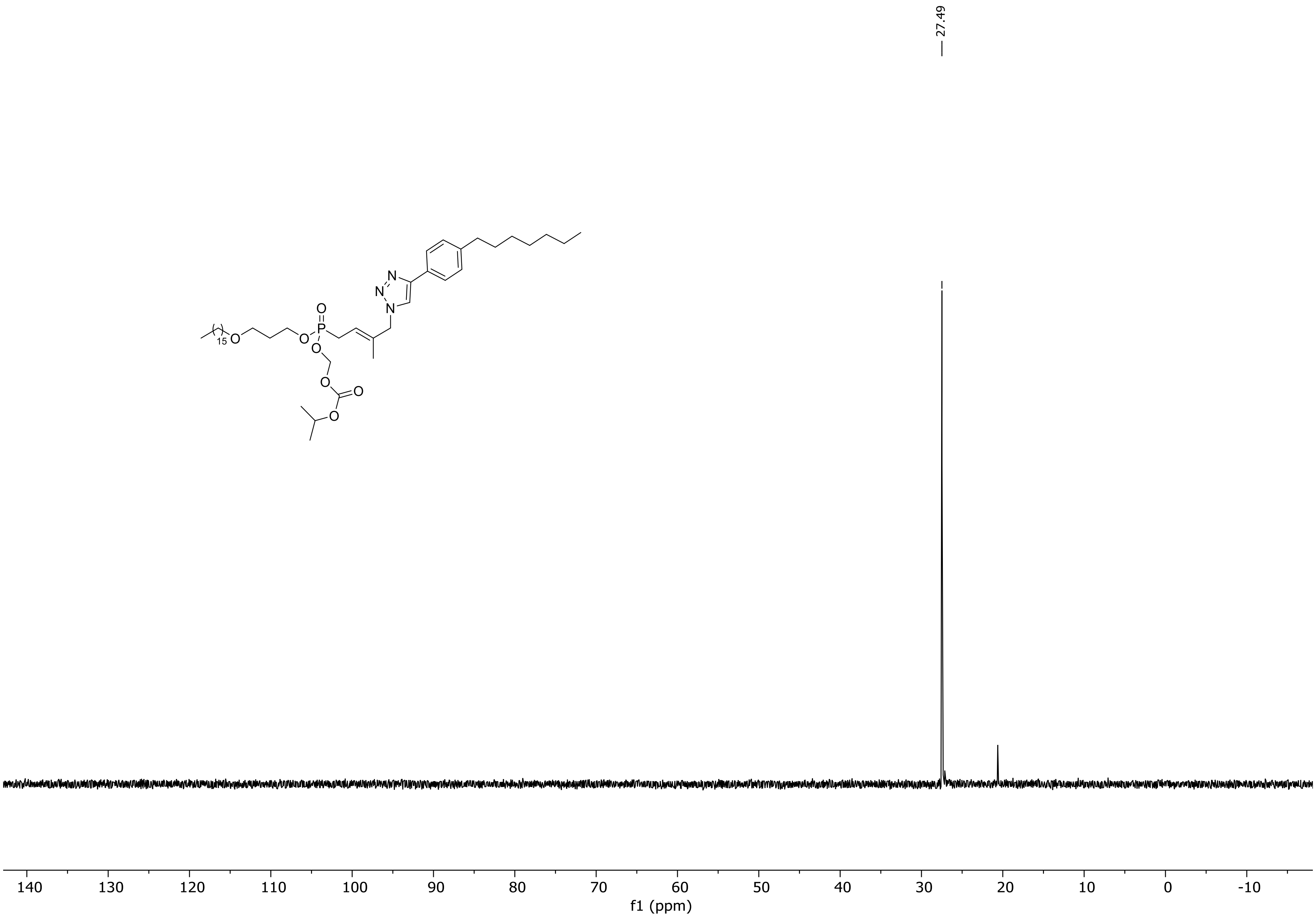

^{1}H NMR (400 MHz, $CDCl_3$) Analysis of Compound 15j

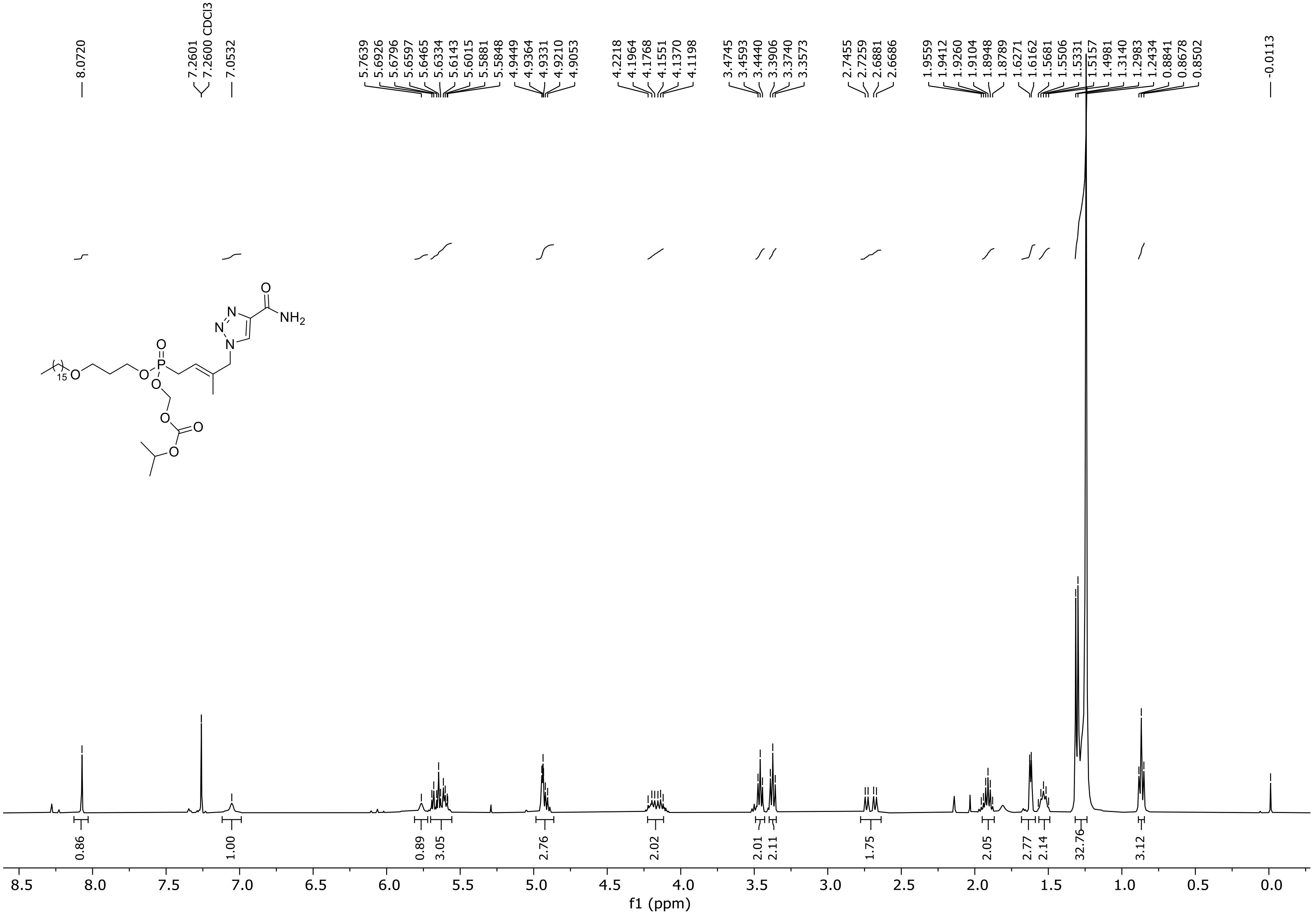

^{13}C NMR (101 MHz, $CDCl_3$) Analysis of Compound 15j

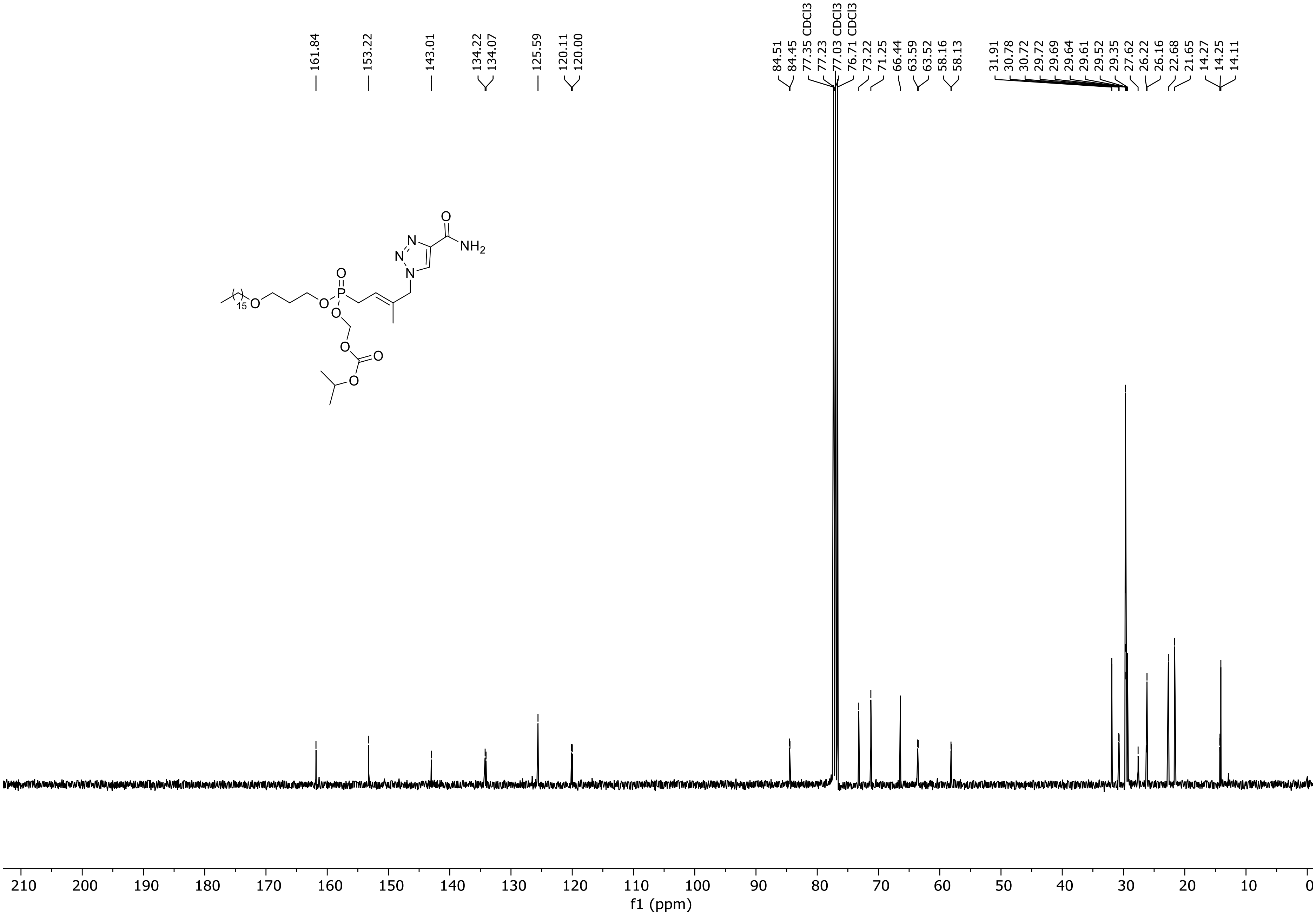

^{31}P NMR (161 MHz, $CDCl_3$) Analysis of Compound 15j

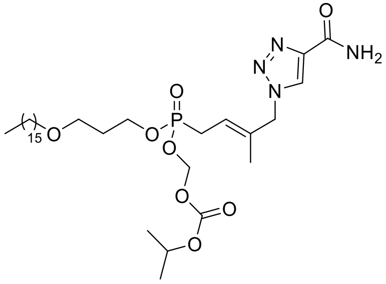

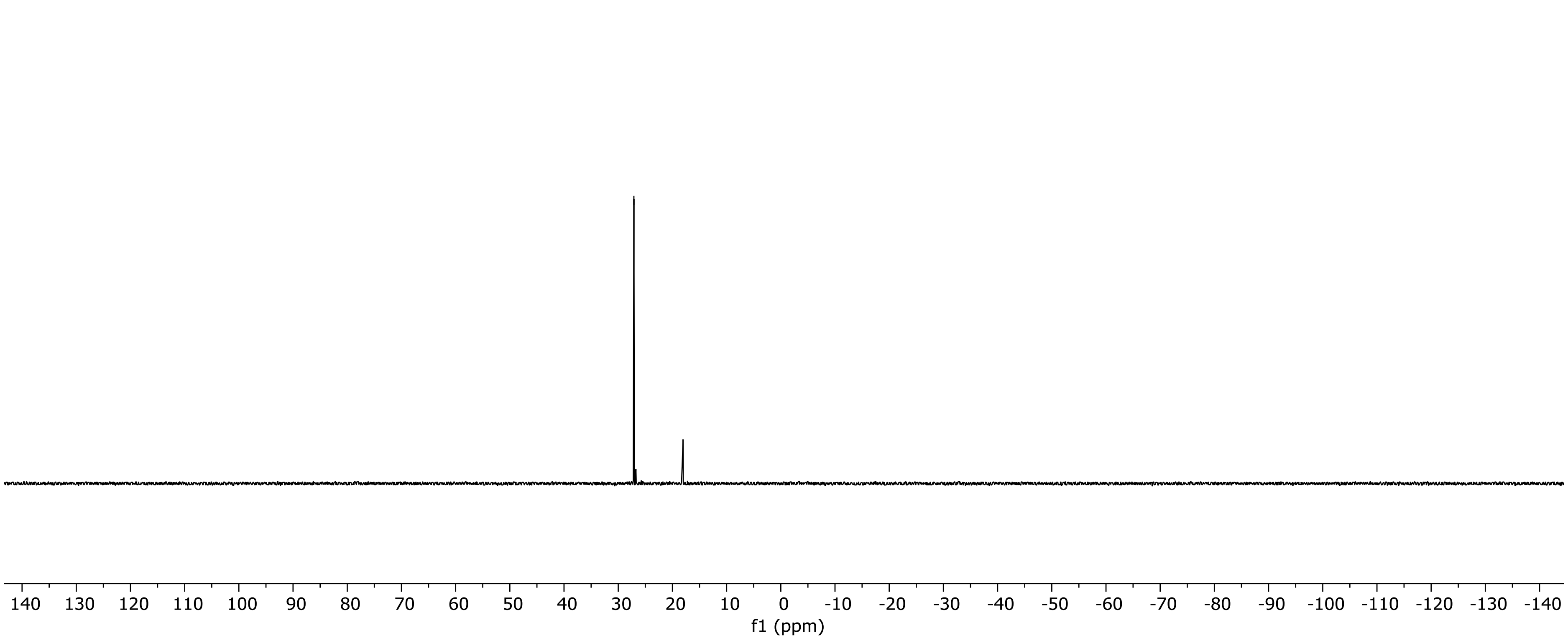

^1H NMR (400 MHz, $CDCl_3$) Analysis of Compound 15l

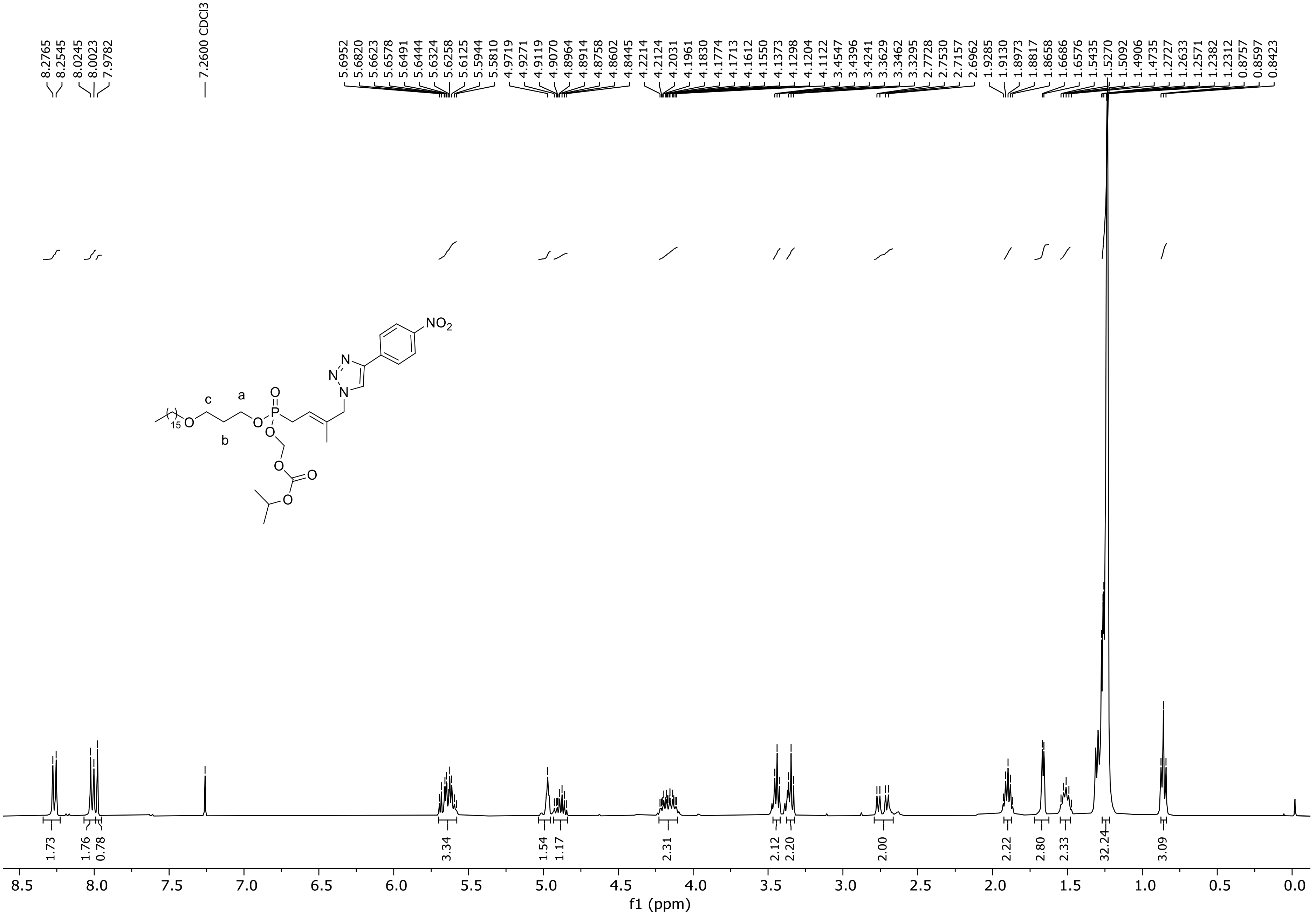

^{13}C NMR (101 MHz, $CDCl_3$) Analysis of Compound 15l

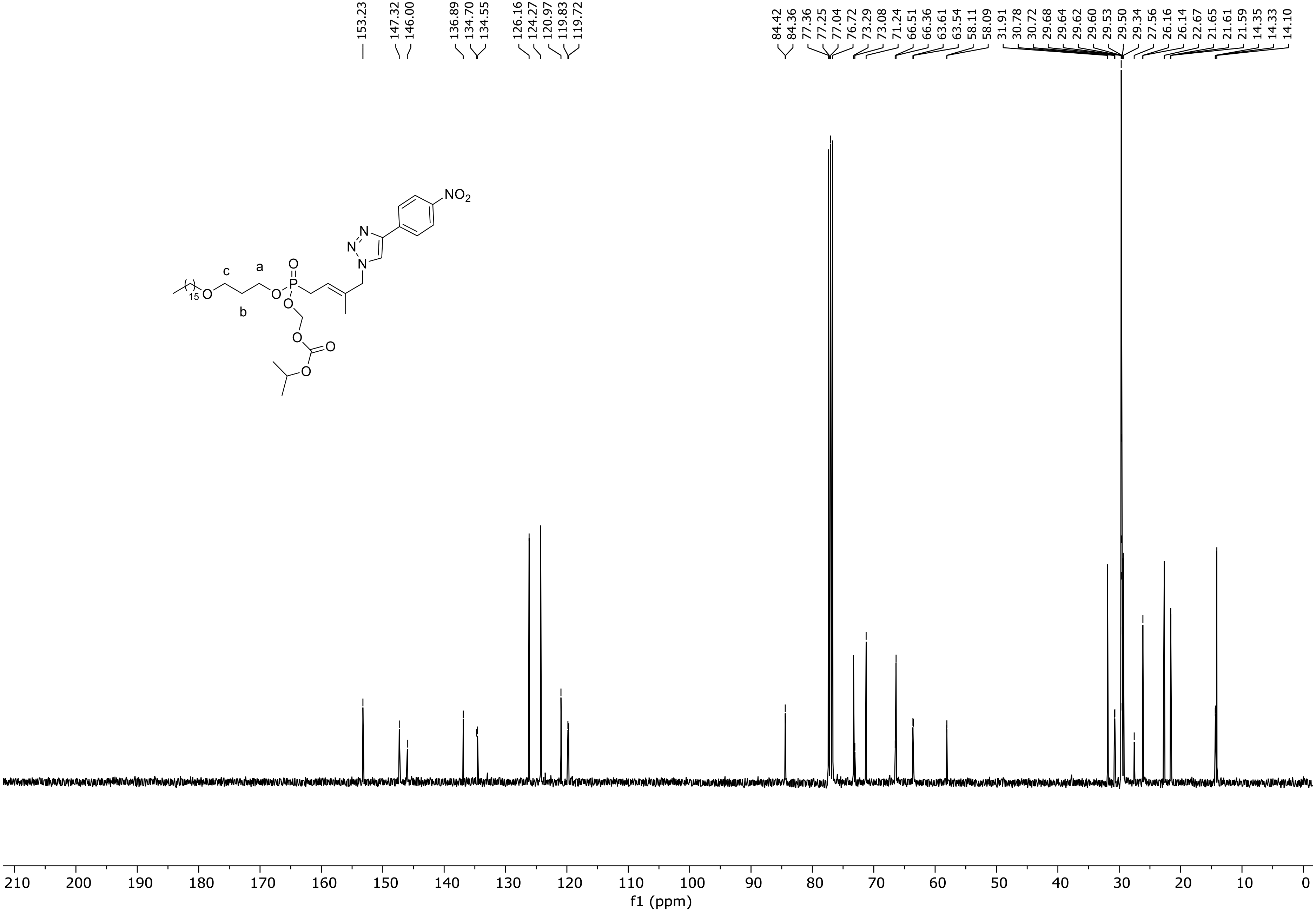

^{31}P NMR (161 MHz, $CDCl_3$) Analysis of Compound 15l

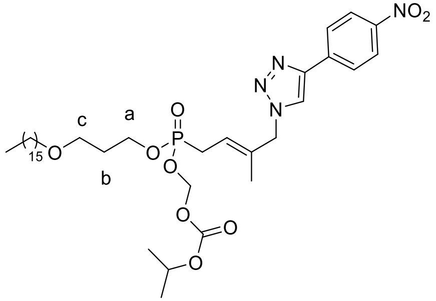

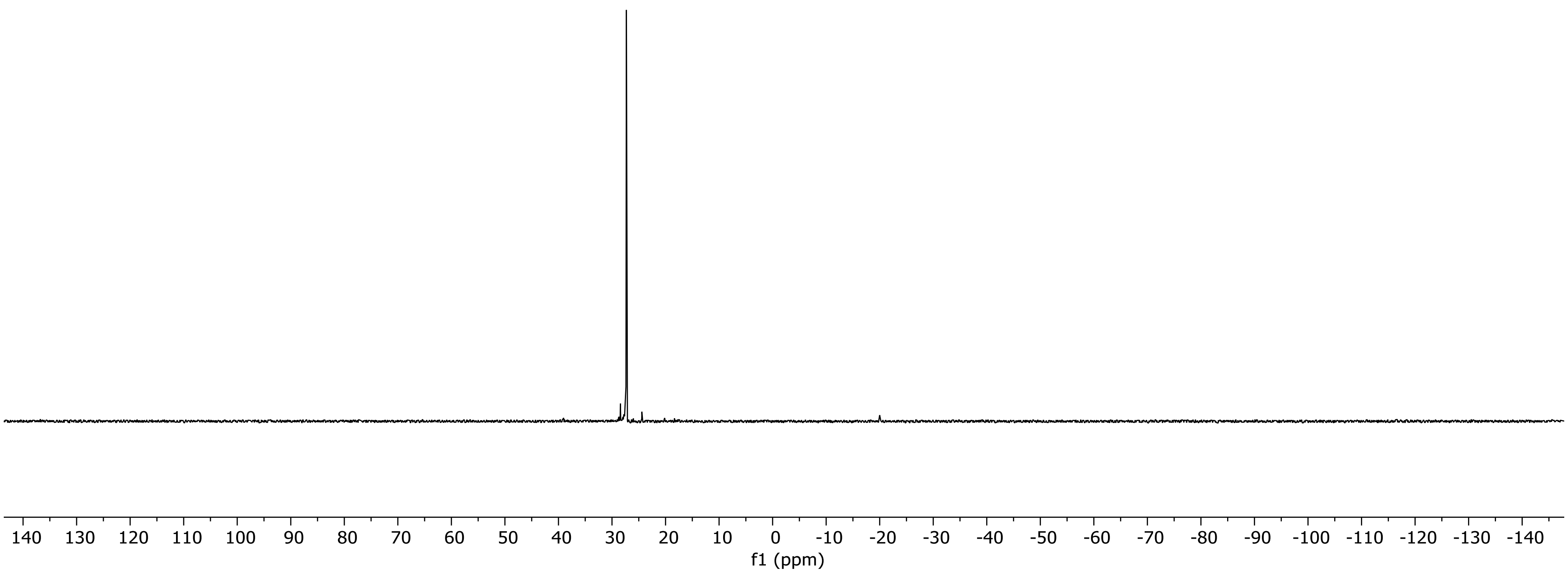

Supplement: Supplementary file 1 [file molecules-26-01493-s001.pdf]
